# Smart symbiotic lithium–sulfur batteries under extremely low-temperature conditions

**DOI:** 10.1093/nsr/nwag217

**Published:** 2026-04-09

**Authors:** Runyue Mao, Mengfan Pei, Xin Jin, Dejian Qu, Jiangpu Yang, Chang Su, Shuo Zhuo, Naiwen Hu, Cijian Zhang, Dongming Liu, Shengming Li, Xigao Jian, Fangyuan Hu

**Affiliations:** School of Materials Science and Engineering, State Key Laboratory of Fine Chemicals, Frontiers Science Center for Smart Materials Oriented Chemical Engineering, Technology Innovation Center of High Performance Resin Materials (Liaoning Province), Dalian University of Technology, Dalian 116024, China; School of Materials Science and Engineering, State Key Laboratory of Fine Chemicals, Frontiers Science Center for Smart Materials Oriented Chemical Engineering, Technology Innovation Center of High Performance Resin Materials (Liaoning Province), Dalian University of Technology, Dalian 116024, China; School of Materials Science and Engineering, State Key Laboratory of Fine Chemicals, Frontiers Science Center for Smart Materials Oriented Chemical Engineering, Technology Innovation Center of High Performance Resin Materials (Liaoning Province), Dalian University of Technology, Dalian 116024, China; School of Control Science and Engineering, Dalian University of Technology, Daliian 116024, China; School of Materials Science and Engineering, State Key Laboratory of Fine Chemicals, Frontiers Science Center for Smart Materials Oriented Chemical Engineering, Technology Innovation Center of High Performance Resin Materials (Liaoning Province), Dalian University of Technology, Dalian 116024, China; School of Materials Science and Engineering, State Key Laboratory of Fine Chemicals, Frontiers Science Center for Smart Materials Oriented Chemical Engineering, Technology Innovation Center of High Performance Resin Materials (Liaoning Province), Dalian University of Technology, Dalian 116024, China; School of Materials Science and Engineering, State Key Laboratory of Fine Chemicals, Frontiers Science Center for Smart Materials Oriented Chemical Engineering, Technology Innovation Center of High Performance Resin Materials (Liaoning Province), Dalian University of Technology, Dalian 116024, China; School of Materials Science and Engineering, State Key Laboratory of Fine Chemicals, Frontiers Science Center for Smart Materials Oriented Chemical Engineering, Technology Innovation Center of High Performance Resin Materials (Liaoning Province), Dalian University of Technology, Dalian 116024, China; State Key Laboratory of Fine Chemicals, Frontiers Science Center for Smart Materials Oriented Chemical Engineering, School of Chemical Engineering, Technology Innovation Center of High Performance Resin Materials (Liaoning Province), Dalian University of Technology, Dalian 116024, China; School of Materials Science and Engineering, State Key Laboratory of Fine Chemicals, Frontiers Science Center for Smart Materials Oriented Chemical Engineering, Technology Innovation Center of High Performance Resin Materials (Liaoning Province), Dalian University of Technology, Dalian 116024, China; School of Control Science and Engineering, Dalian University of Technology, Daliian 116024, China; School of Materials Science and Engineering, State Key Laboratory of Fine Chemicals, Frontiers Science Center for Smart Materials Oriented Chemical Engineering, Technology Innovation Center of High Performance Resin Materials (Liaoning Province), Dalian University of Technology, Dalian 116024, China; State Key Laboratory of Fine Chemicals, Frontiers Science Center for Smart Materials Oriented Chemical Engineering, School of Chemical Engineering, Technology Innovation Center of High Performance Resin Materials (Liaoning Province), Dalian University of Technology, Dalian 116024, China; School of Materials Science and Engineering, State Key Laboratory of Fine Chemicals, Frontiers Science Center for Smart Materials Oriented Chemical Engineering, Technology Innovation Center of High Performance Resin Materials (Liaoning Province), Dalian University of Technology, Dalian 116024, China

**Keywords:** lithium–sulfur battery, wide temperature range, energy density, multifield synergy, polysulfide conversion

## Abstract

Lithium–sulfur batteries (LSBs) fail catastrophically under ultralow temperature due to frozen polysulfide conversion kinetics, with no existing technology achieving high-energy-density operation below −40°C. Here, we report a self-regulating LSB system, the ‘smart symbiosis’ cell, that activates multifield synergy at interface reaction sites to overcome kinetic barriers under low temperature. This directly modulates the transport of ions/electrons and the spin electron states of reaction sites at the quantum level, enabling wave-shaped charge/discharge profiles and achieving a ratio of 3.11 between the first plateau and the second plateau (theoretical value 3.0). The ultratheoretical capacity mechanism is revealed—magnetic field-induced enhancement of kinetics and interfacial reactions. The pouch cell achieves an energy density of 454.5 Wh kg^−1^ (based on total system mass) and 219.1 Wh kg^−1^ (with device consumption) at −80°C. This technology could increase the capacity of batteries by 9.5 times at low temperatures with an energy consumption of ∼0.091% °C^−1^ of the battery energy, while the conversion retention rate remains as high as 87% after 200 cycles (∼2800 h), and breaks the lowest temperature record. This new battery system opens the door to extremely wide temperature applications for LSBs and could be extended to other batteries.

## Teaser

By introducing a multi-physics field at the cathode polysulfide conversion interface, the authors improved the low-temperature capacity by over 9.5 times and set a new record for the lowest operating temperature of Li-S batteries.

## INTRODUCTION

The exploration of extreme-temperature environments such as stratosphere platforms (−90°C–60°C), low altitude (−30°C–40°C), polar regions (−95°C–32°C) and even Mars (−133°C–30°C) is producing a lot of industries with unlimited potential. For instance, Morgan Stanley predicts that the market scale of stratosphere platforms industry will reach 50 billion dollars. Therefore, there is an urgent need for batteries with high energy density under ultrawide temperature ranges [1–5]. Although lithium–sulfur batteries (LSBs) offer an ultrahigh theoretical energy density and specific capacity, their practical performance is limited by sluggish dynamics under lean electrolyte conditions [6–10]. More importantly, different from the conversion process of lithium polysulfides (LiPSs) at room temperature, the agglomeration phenomenon, and slow ion transport at low temperature may affect the conversion kinetics of sulfur species [11–13]. This is an important reason for the low-temperature failure of LSBs.

To extend the temperature range of LSBs, most studies have focused on materials engineering such as cathode materials design, separator modification, and electrolyte formulation [14–16]. For cathodes, strategies aim to enhance low-temperature performance by introducing adsorption and catalytic sites, thereby promoting the three-phase interface reaction kinetics [17–19]. Similarly, separator modifications incorporate conductive networks and catalytic sites to suppress polysulfide shuttling while accelerating conversion kinetics [20]. In addition, a critical bottleneck remains the sluggish Li^+^ transport and high desolvation barriers at low temperatures. Therefore, researchers have regulated the solvation structure to improve the desolvation kinetics through electrolyte design [21–23]. Although the above strategies are effective from 0°C to –60°C, they falter below −60°C. Currently, the lowest operating temperature for LSBs is −60°C [24,25] and only –20°C in pouch cells [23,26]. This failure originates from near-stagnant polysulfide conversion under ultralow temperatures, where merely lowering reaction energy barriers through materials optimization fails to counteract kinetic paralysis.

Besides materials engineering, battery structure innovation offers a promising yet underexplored avenue for LSBs. Although structure designs from other battery systems provide some reference, they still face critical challenges. For instance, external positive temperature coefficient (PTC) heaters—typically aluminum sheets coated with PTC materials—preheat battery packs at a heating rate of ∼4°C min^−1^ but suffer substantial thermal losses (>35%) due to inefficient heat transfer from the exterior to the interior. Besides, internal self-heating methods employ nickel foils as auxiliary electrodes to generate localized heat through electron flow, achieving faster heating rates (60°C min^−1^ at −40°C) with reduced energy dissipation [27–29]. However, introducing foreign conductive components like nickel foils compromises cell safety, which is also crucial for high-energy-density LSBs, by elevating short-circuit risks. The external heating sacrifices efficiency, while internal heating jeopardizes safety. In addition, neither approach could generate new capacity to recover the net capacity loss that comes from heat generation.

**Figure 1.**
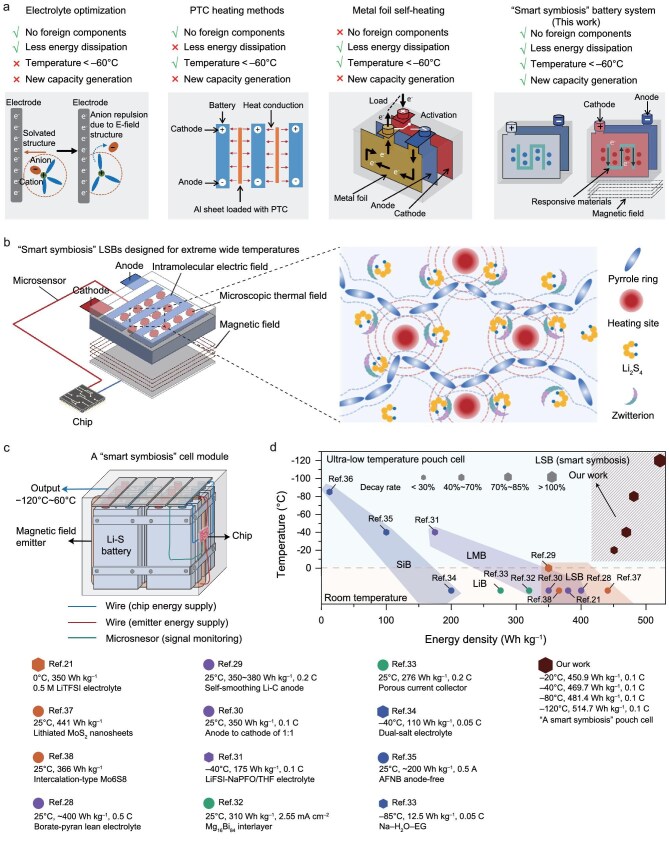
Theoretical schematic and specific advantages of ‘smart symbiosis’ cell. (a) Comparison of the advantages and disadvantages of electrolyte optimization (representing material optimization methods), PTC heating methods, metal foil self-heating, and this work (‘smart symbiosis’ LSBs). (b) Schematic of the MFSN mechanism in ‘smart symbiosis’ LSBs for extremely wide-temperature conditions. (c) Schematic of the ‘smart symbiosis’ cell module consisting of eight pouch cells, temperature sensors, chips, and magnetic field emitters. (d) A summary of reported LSBs, LMBs, LIBs, and SIBs in terms of energy density and operating temperature. Data are from references [23,30–40].

Here, we propose a ‘smart symbiosis’ LSB system through the synergistic design of cathode materials and cell structure, which enables the batteries to meet the application challenges of extremely wide-temperature environments (−120°C–60°C). In contrast to the currently reported schemes, it offers four advantages: (1) no foreign components in the cell, which avoids internal short-circuit risks; (2) less energy dissipation, which improves energy utilization efficiency; (3) overcoming kinetic paralysis, which leads to an operating temperature below −60°C; and (4) new capacity generation, which recovers energy loss (Fig. 1a). The cell structure introduces a tunable magnetic field, while the cathode materials respond by generating thermal fields and rapid electron/ion transport. Theoretical simulations confirm that the magnetic field enhances the overlap of electron orbitals to promote interfacial transfer kinetics. Meanwhile, the degeneracy of surface electron energy levels is lifted, thereby sharpening the density of states and improving electron transfer efficiency. Additionally, polarons and energy gap defect states are formed at Fe1 sites: the local charge of polarons could facilitate chemical bond cleavage, while the energy gap defect states act as ‘low-cost’ electron transfer springboards. Through the quantum-scale modulation of the interfacial electronic states, the conversion kinetics (especially Li_2_S_4_ to Li_2_S) is significantly accelerated, which leads to the ratio (*Q*_2_/*Q*_1_) exceeding the theoretical value. The capacity corresponding to the part beyond the theoretical ratio mainly originates from the magnetic field-induced promotion of anode interfacial reactions and stabilization of solid electrolyte interphase (SEI) formation. This enables the pouch cell to achieve a conversion retention rate that remains as high as 87% after 200 cycles (∼2800 h). It has been verified that this technology could increase the capacity of LSBs by 9.5 times, with an energy consumption of ∼0.091% °C^−1^ of the battery energy. This effect has also been verified in 20 Ah-level pouch cells (the energy density including module mass remains as high as 649.3 Wh kg^−1^ at −80°C and still remains at 314.9 Wh kg^−1^ after considering device energy consumption). This represents a significant milestone achievement in the performance of low-temperature LSBs.

## RESULTS AND DISCUSSION

### Design and construction of ‘smart symbiosis’ battery system

A ‘smart symbiosis’ cell that can provide real-time feedback, conduct self-monitoring, and perform self-regulation has been developed for extremely wide-temperature applications (see Note S1 in the Supplementary Materials). The structure model is shown in Fig. 1b. Meanwhile, the cathode materials that are responsive to the magnetic field are designed to replace the original sulfur host materials. A microsensor monitors the internal temperature of the pouch cell, while the external chip processes data and regulates the magnetic field in real time. Subsequently, the internal energy supply and external output of the device are reflected in the cell module (Fig. 1c). A module consists of more than 4–8 pouch cells (∼20 Ah), and the magnetic field emitter is located in the center. The internal temperature of each cell is monitored by microsensors and the relevant information is given to the chip. The chip and the emitter are supplied by the cells through wires. This beneficial symbiotic relationship could avoid low-temperature capacity decay while stimulating the potential to achieve higher capacity. Additionally, we summarize the electrochemical performance of reported advanced LSBs, lithium metal batteries (LMBs), lithium-ion batteries (LIBs), and sodium-ion batteries (SIBs) at different temperatures (Fig. 1d and Table S1) [23,30–40], and compare these results with this work. It could be found that LMBs, LIBs, and SIBs could discharge stably at −80°C, but it is difficult to maintain high energy density due to capacity decay (>70%). Although LSBs deliver high energy densities above −20°C, their capacity drops by ∼80% or even fails below −40°C due to stagnation of polysulfide reactions. In contrast, this work could achieve an energy density of 514.7 Wh kg^−1^ at −120°C. Even when quality and energy consumption are taken into account, the pouch cells still achieve the highest reported energy density for pouch cells at −20°C, −40°C, −80°C, and −120°C. Both energy density and operating temperature are expected to be suitable for ‘all-climate’ and extremely wide-temperature environments.

### MFSN in different reaction processes

The effects of multifield synergy (MFSN) on the reaction process were characterized by ‘smart symbiosis’ cells, and by plotting the curves in different charge and discharge stages (cell parameters shown in Table S2). As shown in Fig. 2a, charge–discharge curves of the ‘smart symbiosis’ cell (the cell with magnetic field) exhibit unique fluctuation compared to the lithium–sulfur (Li–S) cell (the cell without magnetic field). The ratio of the second plateau to the first (*Q*_2_/*Q*_1_) at −120°C reaches 3.11, which exceeds the theoretical value (3.0) and is significantly higher than that at 25°C (2.39). This proves that MFSN could significantly improve the low-temperature reaction kinetics. As shown in Fig. 2b, the curves are almost coincident in the process from S_8_ to Li_2_S_4-8_, indicating that the fields have little effect on the stage. The local diagram (Fig. 2c) further proves this conclusion. This is because the process mainly depends on electrolyte infiltration and sulfur loading homogeneity. However, regular oscillations similar to ‘sea wave’ appear during the second discharge plateau (Fig. 2d–g), indicating that the conversion of Li_2_S_4_ to Li_2_S was significantly affected by MFSN. Initially, when the magnetic field is activated, the conversion of Li_2_S_4_ to Li_2_S is accelerated instantaneously, and the voltage increases linearly before plateauing in the later stage as the polysulfide reaction kinetics approach its limit (Fig. 2e). Notably, the increase of voltage is due to the decrease of polarization, which is related to the improvement of dynamics. When the acceleration of MFSN to the conversion gradually reaches a critical point, the polarization voltage difference (dPV_1_) is 0.023 V. Subsequently, with the advent of magnetic field rest period, this effect is rapidly weakened. A similar ‘sea wave’ phenomenon is observed during the discharge stage from Li_2_S_2_ to Li_2_S (Fig. 2f and g). During this response period, the conversion rate of Li_2_S_2_ to Li_2_S accelerates and then plateaus, similar to the phenomenon above. When the magnetic field rests, dPV_2_ reaches 0.059 V, significantly higher than dPV_1_, indicating that MFSN has a more substantial effect on the final solid–solid reactions during discharge. This may be because the solid–solid reactions’ path and the fields generated by the response of materials under the magnetic field are both located on the electrode. Furthermore, charging curves just like inverted ‘sea wave’ (Fig. 2h and i) confirm that MFSN also accelerates the conversion of Li_2_S and Li_2_S_2_ to Li_2_S_4_, leading to a linear decrease in voltage. At this stage, the decrease of voltage is due to the decrease of polarization. Similarly, the acceleration slows down in the second half of the magnetic field activation. The minor absolute value difference between dPV_1_ from Li_2_S_4_ to Li_2_S and dPV_3_ (−0.021V) from Li_2_S to Li_2_S_4_ indicates that MFSN improves reaction kinetics in both charging and discharging, and the process is reversible. The kinetic promotion of MFSN is not only due to the promotion of ion/electron transport by thermal fields and the magnetic field, but also the change of spin electrons’ state.

**Figure:**
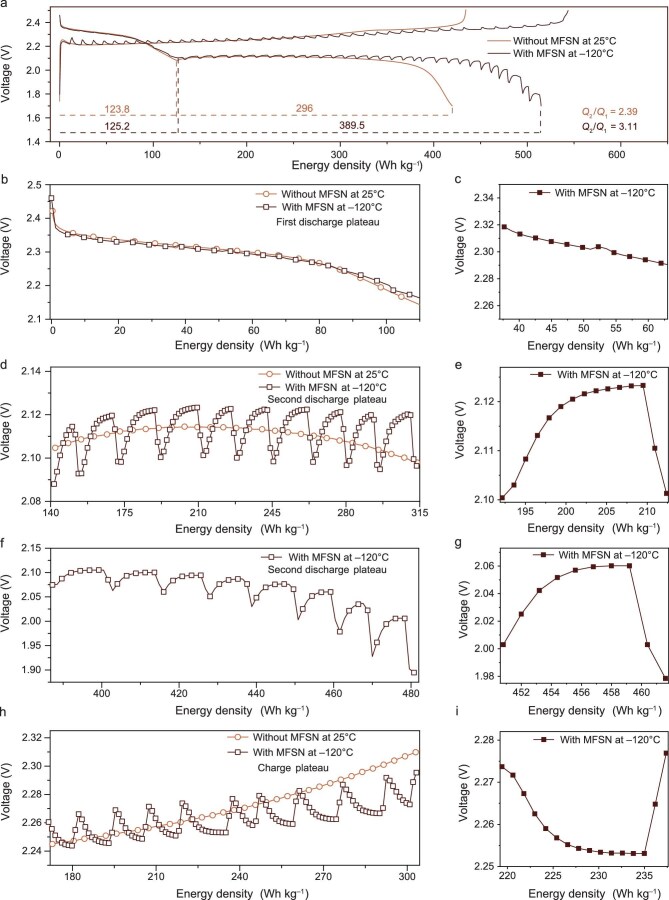
Analysis of MFSN mechanism on different polysulfide conversion processes in ‘smart symbiosis’ cell. (a) Charge and discharge curves (0.1 C) of the ‘smart symbiosis’ cells at −120°C (the cell with magnetic field) and Li–S cell at 25°C (the cell without magnetic field). (b) Discharge curves (0.1 C) during the first discharge plateau at −120°C and 25°C. (c) Magnified view of first discharge plateau curve of the ‘smart symbiosis’ cells at −120°C. (d) Discharge curves (0.1 C) at the early second discharge plateau at −120°C and 25°C. (e) Magnified view of discharge curve at −120°C during the early second discharge plateau. dPV represents the difference in the improvement of the polarization voltage from the beginning of the magnetic field to the limit of the reaction during the reaction process in this charge and discharge. (f) Discharge curves (0.1 C) at the last part of the second discharge plateau at −120°C and 25°C. (g) Magnified view of discharge curve at −120°C during the last part of the second discharge plateau. (h) Charge curves (0.1 C) during the charge plateau at −120°C and 25°C. (i) Magnified view of charge curve at −120°C during the charge plateau.

### Materials performance and ion diffusion analysis

Cathode materials require not only excellent electrochemical properties but also a response to magnetic field through molecular structures. Here, the materials we synthesized (the detailed structure is shown in Fig. S1) are selected as representative cathode materials for ‘smart symbiosis’ batteries. Diffuse reflectance Fourier-transform infrared spectroscopy, transmission electron microscopy, scanning electron microscopy, X-ray photoelectron spectroscopy, Fourier transform infrared spectroscopy (FT-IR), and nitrogen adsorption–desorption curves confirmed the successful synthesis of the material (the detailed analysis is shown in Figs S2–S7). Electrostatic potential calculations show that zwitterions pair with polysulfides to generate ion pairs (sulfonate[−]–Li^+^[+] and S_*x*_^2^^−^[−]–imidazole ring[+]) (Fig. S8), as detailed in our previous work [41]. Following Lewis acid–base theory, negative regions (red) couple with positive regions (blue). Interaction region indicator calculations further confirm the anion–cation pairing interaction between Li^+^ and SO_3_^−^, which is stronger than van der Waals forces (Fig. S4). The conjugated chain segment ensures the electron transport at the conversion site, and the ionic environment could be adjusted by zwitterionic structure.

**Figure 3.**
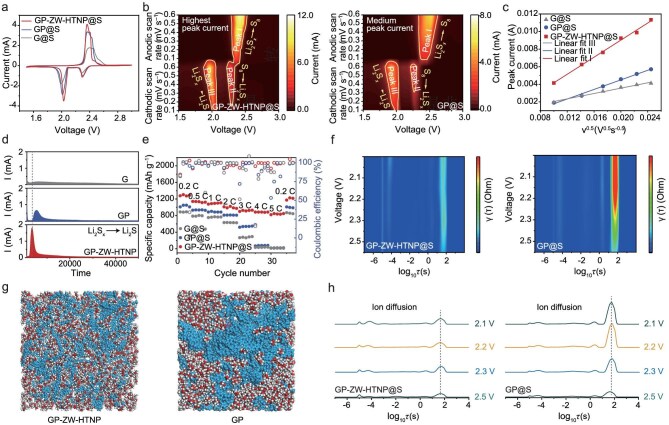
Materials performance and transport mechanisms of ions. (a) CV curves of GP-ZW-HTNP@S, GP@S (the material obtained by uniformly loading sulfur onto GP), and G@S (the material obtained by uniformly loading sulfur onto G). (b) Contour plots of CV patterns for GP-ZW-HTNP@S and GP@S at various scan rates. (c) Corresponding Randles–Sevcik plots of peak current versus square root of scan rate, used to derive *D*_Li_^+^ values. (d) Potentiostatic discharge profiles at 2.08 V in Li_2_S nucleation tests. (e) Rate performances of GP-ZW-HTNP@S, GP@S, and G@S. (f, h) DRT analysis of EIS tests of GP-ZW-HTNP@S and G@S at different discharge potentials. (g) MD simulation of GP-ZW-HTNP and GP.

To verify the fundamental electrochemical properties of GP-ZW-HTNP (the composite material prepared by combining zwitterion-modified polypyrrole supported on graphene with modified nano-Fe_3_O_4_), the GP-ZW-HTNP@S (the material obtained by uniformly loading sulfur onto GP-ZW-HTNP) composite materials were prepared and assembled into coin cells with lithium metal anodes. First, cyclic voltammetry (CV) was conducted to assess the dynamics improvement of the designed structure at room temperature. GP-ZW-HTNP@S shows the highest current response (Fig. 3a). Figure 3b further confirms that GP-ZW-HTNP improves the multi-electron reaction of polysulfides at different scanning rates. Based on the CV results, the Li^+^ ion diffusion coefficient (*D*_Li_^+^) is calculated using the Randles–Sevcik equation (Fig. 3c and Fig. S9), where the slope of fitted line reflects mobility of Li^+^ in the electrode. GP-ZW-HTNP@S exhibits significantly higher *D*_Li^+^_ than GP (graphene loaded with polypyrrole) and G (graphene) at all redox states (A, B, and C), reflecting improved Li^+^ diffusion. This is supported by Tafel slopes of reduction and oxidation processes (Fig. S10). Symmetric CV tests at different scan rates further confirm the superiority of this design. GP-ZW-HTNP shows stable cycling performance even at the high scan rate of 200 mV s^−1^ in CV tests (Fig. S11). To examine the surface liquid–solid kinetics, Li_2_S precipitation tests were conducted. GP-ZW-HTNP exhibits a response peak current of 1.78 mA (178.3 mAh g^−1^ and 3351 s), outperforming GP (0.67 mA, 128.7 mAh g^−1^, and 4897 s) and G (0.17 mA, 83.1 mAh g^−1^, and 6729 s) (Fig. 3d), reflecting enhanced Li_2_S nucleation dynamics. Additionally, GP-ZW-HTNP@S cells display satisfactory rate performance, with a discharge capacity of 845 mAh g^−1^ at 5 C and a stable cycle (Fig. 3e and Fig. S12).

To demonstrate the material’s practical superiority under poor electrolyte conditions, *in situ* electrochemical impedance spectroscopy (EIS) tests were conducted with low E/S ratio (electrolyte-to-sulfur ratio). Distribution of relaxation times (DRT) analysis of the EIS results (Li^+^ diffusion regions: ${\mathrm{log}}_{10}^{\tau ( s )}$ = 0–2) shows that as voltage decreases, Li^+^ diffusion impedance increases with the increase of polysulfide concentration (Fig. 3f–h). However, GP-ZW-HTNP resists the rising impedance caused by high polysulfide concentrations under lean electrolyte conditions, showing a slower increase trend of Li^+^ diffusion impedance. Molecular dynamics (MD) snapshots (Fig. 3g and Fig. S13) further reveal the special Li^+^ transfer mechanism in the GP-ZW-HTNP electrode, where an expanded calculation model was used to better distinguish Li^+^ transfer within the electrode. Results show that the Li^+^ diffusion coefficient in GP-ZW-HTNP is 0.0178 × 10^–5^ cm^2^ s^−1^, much higher than that in GP (0.001019 × 10^–5^ cm^2^ s^−1^), which is due to its abundant lithium-philic sites. These results confirm that the transport of Li^+^ through chains swinging rather than channel infiltration could reduce electrolyte consumption. The interfacial diffusion capacities of GP-ZW-HTNP and GP were further analysed and quantified. The theoretical calculation results show that the interfacial diffusion capacity of GP-ZW-HTNP (5.1394 × 10^–5^ m^2^ s^−1^) is significantly higher than that of GP (6.7989 × 10^–6^ m^2^ s^−1^) (Fig. S14). To verify the application potential under real conditions, we assembled one-piece pouch cells. The first-cycle specific capacity reaches up to 1351.9 mAh g^−1^, with a stable cycle over 90 cycles at 8.3 mg cm^–2^ and E/S = 2.5 at 0.2 C (Fig. S15). These results show the superior electrochemical performance of the designed materials.

### Li_2_S growth under MFSN

A few ‘smart symbiosis’ Li–S pouch cells were manufactured to verify the feasibility of their future applications (Fig. 4a). To monitor the real-time Li_2_S nucleation process in the ‘smart symbiosis’ pouch cell, we embedded a fiber-optic sensor in the cathode to capture real-time microstress within the electrode and real-time electrochemical testing data (Fig. 4b).

**Figure:**
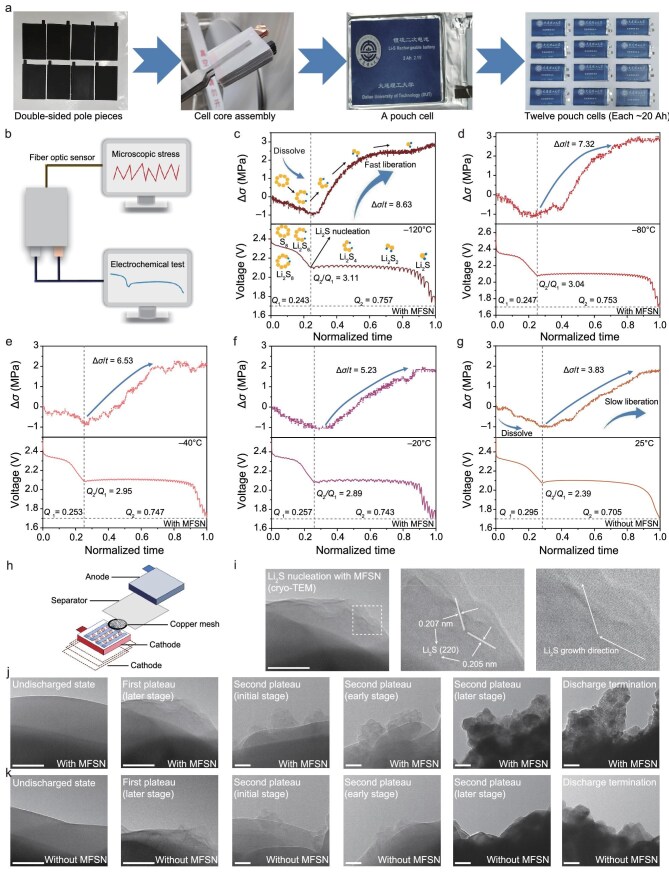
Li_2_S growth in ‘smart symbiosis’ pouch cell. (a) A schematic diagram of assembling high-Ah Li–S pouch cells. (b) Theoretical schematic of the operando microstress testing setup. (c) Real-time *operando* microstress–electrochemistry signal curves of the ‘smart symbiosis’ pouch cell (the cell with MFSN) at −120°C. (d–g) Real-time *operando* microstress–electrochemistry signal curves at (d) −80°C, (e) −40°C, (f) −20°C, and (g) 25°C. (h) Schematic illustration of cryo-TEM test of the cell assembly. (i) Cryogenic transmission electron microscopy (Cryo-TEM) characterization of Li_2_S with MFSN. From left to right are further magnified images of the white-boxed region. (j) Cryo-TEM characterization of Li_2_S growth at different discharge stages with MFSN. (k) Cryo-TEM characterization of Li_2_S growth at different discharge stages without MFSN.

During the initial discharge, stress release occurs at the cathode due to dissolution and contraction of long-chain LiPSs. As shown in Fig. 4c–g and Fig. S17, it can be found that as the ambient temperature decreases, both the stress elevation ratio and conversion ratio during the second discharge stage progressively increase, even exceeding theoretical values. The measured values are 3.83 and 2.39 at 25°C, 5.23 and 2.89 at −20°C, 6.53 and 2.95 at −40°C, 7.32 and 3.04 at −80°C, and 8.63 and 3.11 at −120°C. The enhancement in both conversion rate and stress elevation rate is primarily attributed to the accelerated deposition rate of Li_2_S. The decreased temperature leads to faster Li_2_S nucleation (conversion from Li_2_S_4_ to Li_2_S), mainly because the longer activation of the magnetic field, thereby extending the duration of MFSN. This confirms the catalytic effect of the MFSN on reaction kinetics.

Through cryogenic high-resolution transmission electron microscopy (HRTEM), we directly visualized the Li_2_S growth process throughout discharge under MFSN (Fig. 4h). At the edge region of the pure copper grid, early-stage Li_2_S exhibited clear lattice fringes with spacings of 0.207 and 0.205 nm, corresponding to the (220) plane (Fig. 4i). Under MFSN, Li_2_S exhibits abundant and uniform growth with multidirectional patterns as discharge progresses. In contrast, without an applied magnetic field, Li_2_S nucleation displays smaller size and lower density (Fig. 4j and k). By the end of discharge, enhanced kinetics promoted the formation of irregular tower-like Li_2_S protrusions, effectively preventing the insulating Li_2_S surface layer that causes charging difficulties. Radial Wiener-filtered HRTEM images directly revealed the atomic-scale morphology of Li_2_S under MFSN, corresponding to the (200) and (111) planes along the [011] zone axis, and the (202) and (022) planes along the [111] zone axis (Fig. S18).

### Mechanism of interfacial electronic states

**Figure 5.**
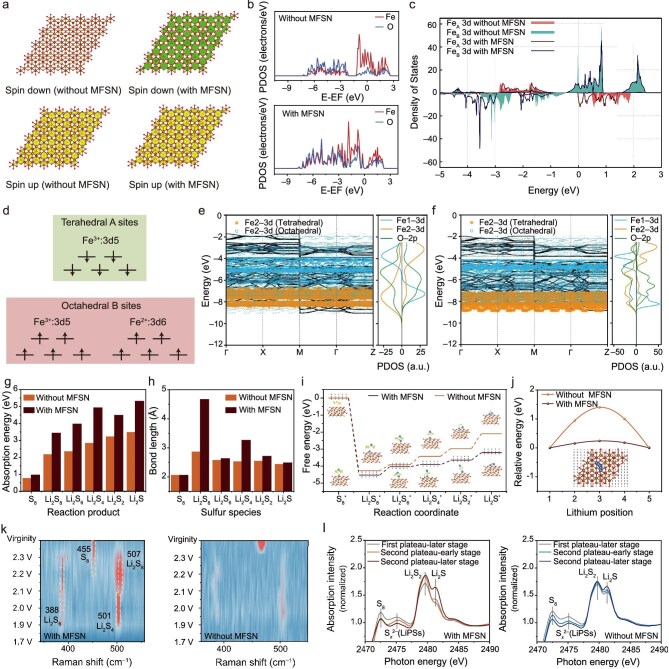
Spin polarization of Fe_3_O_4_ for LiPS conversion and ion transfer ability. (a) Spin density of Fe_3_O_4_ with/without a magnetic field. (b) PDOS of Fe 3d and O 2p in Fe_3_O_4_ with/without a magnetic field. (c) Comparison of Fe_3_O_4_ density of states with/without a magnetic field. (d) Spin configurations in Fe_3_O_4_. (e) Fe_3_O_4_ surface electronic structure with a magnetic field. (f) Fe_3_O_4_ surface electronic structure without a magnetic field. (g) Absorption energy of Fe_3_O_4_ (100) surface to different sulfur species with/without a magnetic field. (h) Bond lengths of Fe_3_O_4_ to different sulfur species with/without a magnetic field. (i) Gibbs free energy profiles and adsorption conformation of LiPSs on Fe_3_O_4_ with/without a magnetic field. (j) Relative energy barriers of Li^+^ diffusion with/without a magnetic field. (k) Raman spectroscopy of the cathode during discharge with/without MFSN. (l) Synchrotron radiation characterization of the cathode during discharge with/without MFSN.

As shown in Fig. 5a and Fig. S16, Fe atoms in Fe_3_O_4_ exhibit uniformly distributed spin density in all directions without a magnetic field. In contrast, magnetic field application induces aligned electron spin orientation [42,43]. Spin polarization enhances the magnetic moment of ligand holes [44]. The orbitals of the catalyst yield nearly degenerate spin-polarized metallic states, which optimize the wavefunctions by weakening interatomic electron–electron repulsion. Spin polarization enhances the overlap between Fe-3d and O-2p orbitals, strengthening the resulting 3d-2p hybrid orbitals (Fig. 5b). The intensified ligand hole-related 3d-2p hybridization facilitates charge transfer kinetics at interfaces. Due to spin angular momentum conservation, catalyst-adsorbed sulfur species interactions exhibit reduced interatomic electron–electron repulsion. This increases conductivity, lowers reaction barriers, and improves polysulfide reaction dynamics. Projected density of states (PDOS) analysis in Fig. 5c demonstrates that under external magnetic fields (i) spin-down states exhibit energy lowering due to the Zeeman effect, manifested as PDOS peak shift toward lower energies (leftward) and (ii) spin-up states display energy elevation, corresponding to PDOS peak shift toward higher energies (rightward). This magnetic field-induced energy level splitting and electronic state redistribution define a magnetically excited state, which interacts with polysulfides and accelerates the reaction kinetics more efficiently. Meanwhile, the detailed simulations were conducted to elucidate the evolution of the electronic structure on the Fe_3_O_4_ surface under magnetic field conditions (Fig. 5e and f). Under a magnetic field, the degeneracy of electron energy levels on the Fe_3_O_4_ surface is lifted, inducing spin splitting and sharpening the density of states. This effect enhances electron transfer efficiency while reducing reaction energy barriers. Concurrently, charge localization occurs at Fe1 sites, generating polarons that introduce new defect states within the energy gap. The localized charge of these polarons enhances adsorption and polarization, facilitating chemical bond cleavage. Furthermore, the in-gap defect states serve as ‘low-cost’ electron transfer mediators, effectively lowering both adsorption and activation energy barriers.

The adsorption energy calculations with/without magnetic field reveal that magnetic fields strengthen the adsorption of Fe_3_O_4_ toward various sulfur species, particularly for Li_2_S_6_ and Li_2_S_4_ (Fig. 5g). Further analysis of bond lengths upon Fe_3_O_4_–LiPSs interactions revealed significantly enlarged structural parameters with a magnetic field compared to that without a magnetic field (Fig. 5h). This facilitates Li–S bond weakening and subsequent cleavage, thus improving reaction kinetics. As illustrated in Fig. 5i, Gibbs free energy barriers at each reaction stage are reduced under a magnetic field compared with that without it. This reduction is particularly evident for reactions involving Li_2_S_4_ and subsequent steps—the key processes governing second discharge plateau capacity. These results confirm that the reduction reaction of LiPSs becomes thermodynamically more favorable under the magnetic field. Calculations of Li^+^ diffusion barriers reveal a barrier height of 0.241 eV under the magnetic field, substantially lower than the 1.401 eV without it (Fig. 5j). This optimized Li⁺ diffusion capability facilitates polysulfide conversion dynamics. The Amyloid Intracellular Domain computational model was used to simulate the electron transport under a magnetic field in conjugated structure. The π-electrons in the conjugated pyrrole rings become highly delocalized and move along the rings. This also contributes to the improvement of electron transfer (Figs S19 and S20).

Raman spectroscopy measurements were performed on cathodes discharged to different voltages (Fig. 5k). Characteristic peaks were observed at 388 cm^−1^ (Li_2_S_6_), 455 cm^−1^ (S_8_), 501 cm^−1^ (Li_2_S_4_), and 507 cm^−1^ (Li_2_S_8_) [45]. The results demonstrate significantly enhanced signal intensities of various polysulfides at different conversion stages under MFSN, confirming its role in improving conversion kinetics. Synchrotron radiation measurements at different discharge stages reveal that S_8_ and S*_x_*^2^^−^ (4 ≤ *x* ≤ 8) signals progressively decrease, while Li_2_S and Li_2_S_2_ signals increase as discharge progresses (Fig. 5l). Notably, under MFSN conditions, the signal enhancement of Li_2_S and Li_2_S_2_ is more significant.

### Performance of ‘smart symbiosis’ cells at −120°C–60°C

**Figure 6.**
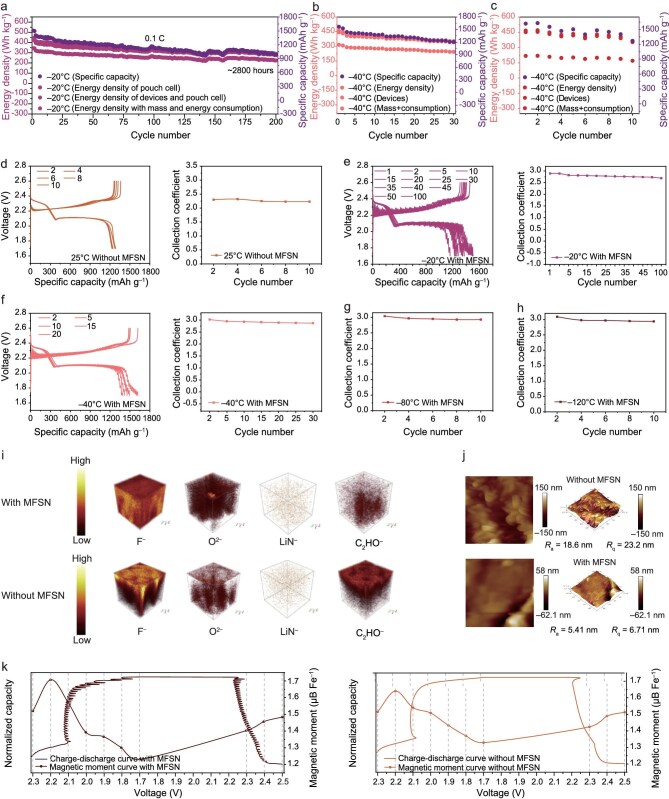
Electrochemical performance of pouch cells and analysis of cathode magnetization. (a) Cycling performance of pouch cells at −20°C (specific capacity, cell-level energy density, device-level energy density including total mass, and system-level energy density considering both energy consumption and mass). (b) Cycling performance of pouch cells at −40°C and 0.1 C (specific capacity, cell-level energy density, device-level energy density including total mass, and system-level energy density considering both energy consumption and mass). (c) Cycling performance of pouch cells at −80°C and 0.1 C (specific capacity, cell-level energy density, device-level energy density including total mass, and system-level energy density considering both energy consumption and mass). (d) Charge–discharge curves and conversion ratio at different cycle numbers of pouch cells at 25°C without MFSN. (e) Charge–discharge curves and conversion ratio at different cycle numbers of pouch cells at −20°C with MFSN. (f) Charge–discharge curves and conversion ratio at different cycle numbers of pouch cells at −40°C with MFSN. (g, h) Conversion ratio at different cycle numbers of pouch cells with MFSN at −80°C (g) and −120°C (h). (i) Time-of-flight secondary ion mass spectrometry three-dimensional imaging of the cycled Li-metal electrodes with/without MFSN. (j) Atomic force microscopy (AFM) image analysis of the lithium anode surface with and without a magnetic field. (k) Charge–discharge profiles and magnetization curves with/without MFSN.

Electrochemical performance tests of pouch cells were systematically conducted at various temperatures. As shown in Fig. 6a, the pouch cell demonstrates a specific capacity of 1505.31 mAh g^−1^ at −20°C and 0.1 C, with an energy density of 425.8 Wh kg^−1^ (based on total system mass) and 344.4 Wh kg^−1^ (with device consumption). In contrast, the pouch cell delivers an energy density of 396.43 Wh kg^−1^ (1200.23 mAh g^−1^) at 25°C (Figs S21 and S22). It also exhibits significant advantages in comparison with G@S (The material obtained by uniformly loading sulfur onto G) and GP@S (The material obtained by uniformly loading sulfur onto GP) under the same conditions (Fig. S23). After 200 cycles (∼2800 h), the capacity remains at ∼1000 mAh g^−1^, corresponding to an average per-cycle capacity fading rate of 0.17%. When accounting for the total system mass (calculated based on the actual mass proportion after module assembly) and energy consumption (∼0.065% °C^−1^ of the battery energy), the overall energy density reaches 344.4 Wh kg^−1^ (Table S2). Comparison with reported studies further validates the superior performance of this work (Fig. S24). After 100 cycles, the conversion retention rate stays at ∼93%, demonstrating the excellent reversibility of MFSN in improving reaction kinetics (Fig. 6e). At 0.1 C and −40°C, the pouch cell delivers a specific capacity of 1567.69 mAh g^−1^ and an energy density of 443.7 Wh kg^−1^ (based on total system mass) and 315.6 Wh kg^−1^ (with device consumption), and a specific capacity of 1567.69 mAh g^−1^, with stable cycling for over 30 cycles (Fig. 6b). When accounting for energy consumption (∼0.091% °C^−1^ of the battery energy) and total mass (calculated based on the actual mass proportion after module assembly), the energy density remains 315.6 Wh kg^−1^ (Table S3). The ratio between the first plateau and the second plateau reaches 3.02, and after 30 cycles, the retention rate remains as high as ∼98% (Fig. 6f). At −80°C and 0.1 C, the initial specific capacity of this pouch cell was 1627.6 mAh g^−1^, with a ratio (the second plateau to the first plateau) of 3.04 (Fig. 6g). The initial energy density reached 454.5 Wh kg^−1^ (total system mass proportion) and 219.1 Wh kg^−1^ (with device consumption), which is even 115% of its room-temperature energy density (Fig. 6c). Even when accounting for device mass (calculated based on the actual mass proportion after module assembly) and energy consumption (∼0.203% °C^−1^ of the battery energy), the energy density is still the highest value reported to date for pouch cells operating at −80°C (Table S4). Moreover, the pouch cell could operate stably at an ultralow temperature of −120°C, delivering a specific capacity of 1653.2 mAh g^−1^ (1.8 V cutoff, LiNO_3_ excluded) at 0.1 C (Fig. S26 and Table S5), which is generally sufficient to support its operation at this temperature. Even at −120°C and −80°C, the ratio between the first plateau and the second plateau remains stable with increasing cycle numbers (Fig. 6g and h). Comparison of the discharge curves at 25°C, −40°C, −120°C, and 60°C shows that even at 60°C and 0.05 C, the ratio (2.55) is much lower than that of the −120°C (3.11) and the −40°C (3.02), which suggests that it is not just purely temperature that improve the kinetics (Figs S25–S27). We also compared the discharge curves with and without MFSN at −20°C and −40°C (the battery failed at −80°C and −120°C without MFSN) (Figs S28 and S29). At −20°C without MFSN, the specific capacity was only 751.44 mAh g^−1^ (compared to 1505.31 mAh g^−1^ with MFSN), and the conversion ratio was merely 1.23 (versus 2.89 with MFSN). At −40°C without MFSN, the specific capacity dropped sharply to 164.19 mAh g⁻^1^, whereas with MFSN, it reached 1567.69 mAh g^−1^—9.548 times higher than the condition without MFSN. In addition, the third-party test report shows that the energy density of the 20 Ah pouch cell reaches 527 Wh kg^−1^ (Figs S30–S33). In addition, the MFSN also exhibited a significant improvement in the kinetics at both room temperature (20°C) and high temperature (50°C) (Figs S34 and S35). The energy density of the 20 Ah pouch cell is 566.4 Wh kg^−1^ (based on total system mass) at −20°C (after accounting for device mass and power consumption: 456.9 Wh kg^−1^) (Table S6). At −80°C, the energy density of the 20 Ah pouch cell is 649.3 Wh kg^−1^ based on total system mass (after accounting for device mass and power consumption: 314.9 Wh kg^−1^) (Table S8). We compared the variation laws of voltage–time curves and temperature–time curves with respect to magnetic field intensity and magnetic field duration at −20°C, −40°C, −80°C, and −120°C. It was found that the enhancement effect on kinetics gradually intensifies with the increase in magnetic field intensity and duration (Figs S36–S43 and Tables S10–S13). The intensity, frequency, waveform, spatial distribution, its settings at different temperatures, and calibration methods are provided in Figs S44 and S55 and Note S3. The superior electrochemistry performance is mainly due to the promotion of polysulfide conversion at the interface by MFSN and the magnetic field-induced defect-enriched Li^+^.

The capacity exceeding the theoretical value primarily originates from two aspects: (i) the enhancement of conversion kinetics by the magnetic field (accounting for the majority of the capacity) and (ii) the reaction of the electrolyte additive at the cathode (accounting for the portion exceeding the theoretical value). Discharge curves of sulfur-free pure Fe_3_O_4_ cathodes at low temperatures show a clear capacity plateau between 1.7 and 1.8 V (Fig. S56), confirming the occurrence of LiNO_3_-related reactions at the cathode interface. In addition, composition analysis of the cycled lithium anode reveals an SEI layer uniformly rich in Li_3_N, LiF, and Li_2_O (Figs S57 and S58 and Fig. 6j), which helps suppress Li-polysulfide side reactions and lithium dendrite growth. This could improve the coulombic efficiency of the battery (Fig. S59). Comparison with the nonmagnetic case shows that LiNO_3_ decomposition still occurs (Fig. S60), but the resulting Li_3_N content is lower, indicating that the magnetic field promotes a more stable SEI formation. Consequently, under the magnetic field, a uniform and stable SEI layer enables long-term cycling stability (∼2800 h) even at E/S = 2.5 (Fig. S61). Meanwhile, the time-of-flight secondary ion mass spectrometry (TOF-SIMS) test results of the lithium anode after cycling show that a uniform and stable SEI is more likely to form under a magnetic field (Fig. 6i).

Magnetic measurements on cathodes at different discharge and charge stages (Fig. 6k and Figs S62 and S63) confirmed the rapid conversion process of polysulfides on the Fe_3_O_4_ surface under the influence of the magnetic field. Initially, as S_8_ dissolves into the electrolyte, the previously masked Fe_3_O_4_ nanoparticles are exposed, resulting in an increase in magnetization. Subsequently, the magnetization gradually decreases. In the absence of a magnetic field, this decrease occurs gradually, whereas the magnetic field significantly accelerates this process. This is attributed to the massive nucleation of Li_2_S on and around the surfaces of Fe_3_O_4_ nanoparticles induced by the magnetic field.

### ‘Smart symbiosis’ cell driving UAV at dry ice temperature

**Figure 7.**
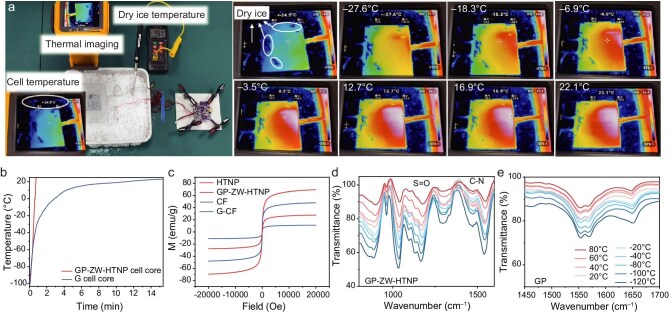
The ‘smart symbiosis’ cell drives drone operation under dry ice conditions. (a) Real-time operation diagram of the pouch cell driving the UAV in dry ice, with thermal imaging of the cell surface temperature. (b) Magnetic hysteresis loops of different materials. (c) Different cell core heating profiles under a magnetic field. (d) *In situ* FT-IR of GP-ZW-HTNP from −120°C to 80°C. (e) *In situ* FT-IR of GP from −120°C to 80°C.

To assess ‘smart symbiosis’ cells under real conditions, we tested unmanned aerial vehicle (UAV) rotation under dry ice conditions (−75.7°C). Figure 7a shows a portion of the whole warming and UAV operation process (see Movie S1 for details). The real-time infrared thermal monitoring lens is in the lower left, dry ice temperature display is in the upper right, and the UAV is in the lower right. Due to practical constraints, thermal imaging could only monitor the surface temperature of the cell. The ‘smart symbiosis’ structure is connected when the cell reaches dry ice temperature, with the surface of the cell covered in frost and the UAV rotating slowly. After 2 min, the temperature of the surface rises to −8.8°C, and the UAV rotates faster. At 2.5 min, the temperature reaches 26°C, and the frost on the cell surface disappears as heat conducts from the inside of the cell to the outside, allowing the UAV to operate more powerfully. MFSN significantly enhances polysulfide transformation dynamics, enabling the cell to efficiently power the UAV in the low-temperature environment of dry ice.

The heating performance of the materials, a key factor for efficient utilization of microscopic thermal fields, was evaluated by exposing cells to a magnetic field (see details in the experimental section). As shown in Fig. 7b, the cell core containing GP-ZW-HTNP shows the best heating performance, raising the temperature from −100°C to 20°C in 0.76 min at an average rate of 161°C min^−1^. In contrast, the core containing G has a heating rate of only 8.06°C min^−1^. The superior magnetocaloric performance is also evident in refrigerator tests (Fig. S64). GP-ZW-HTNP could maintain steady temperature increases, while it is difficult for others to achieve this. This is due to the inhibition of agglomeration, which improves the magnetothermal effects via Neel, Brownian relaxation, etc. [46]. Magnetic hysteresis measurements confirm that GP-ZW-HTNP shows higher saturation magnetization, favoring magnetocaloric applications (Fig. 7c). *In situ* FT-IR spectroscopy was used to investigate lithium bond chemistry in LSBs under microscopic thermal fields. As shown in Fig. 7d, the S=O peaks at 1048 and 1180 cm^−1^ shift with temperature from −120°C to 80°C, suggesting that the thermal field strengthens the chains swing. Polymer segment activity increases with temperature, and lithium-affinity groups such as sulfonate serve as Li^+^ transport sites, further enhancing transmission efficiency.

## CONCLUSION

In summary, this work pioneers a ‘smart symbiosis’ battery system, which significantly improves the operating temperature range and energy density of LSBs. The ‘smart symbiosis’ battery was successfully constructed through the design of cathode materials and integration of pouch cells, temperature sensors, chips, and magnetic field emitters. This design not only inhibits thermal energy dissipation but also eliminates risks such as short circuits. The magnetic field and thermal fields generated by the response of cathode materials could effectively improve ion/electron transport, creating a favorable environment for polysulfide conversion. We discovered that the magnetic field could modulate the spin electron states of magnetic nanoparticles surface at the quantum level. It promotes interfacial transfer kinetics by enhancing electron orbital overlap and sharpens the density of states to improve electron transfer efficiency. Furthermore, polarons (facilitating chemical bond cleavage) and energy gap defect states (acting as low-cost electron transfer springboards) form at Fe1 sites, collectively reducing the activation energy barrier. The growth of Li_2_S under MFSN exhibits abundant and uniform growth with multidirectional patterns, confirming a significant improvement in kinetics. As a result, the conversion ratio breaks through the theoretical value, and the capacity of pouch cell at −40°C is 9.583 times higher than that without MFSN, with an ∼0.091% °C^−1^ of battery energy consumption. The pouch cell still maintains a conversion retention rate as high as ∼87% after 200 cycles (∼2800 h) at 0.1 C and low temperature. It is mainly due to the remarkable promotion of polysulfide kinetics and the stable formation of the interfacial SEI under magnetic field induction. Even in 20 Ah-level pouch cells, the energy density remains as high as 649.3 Wh kg^−1^ at −80°C (after accounting for device mass and power consumption: 314.9 Wh kg^−1^). Meanwhile, the pouch cell could operate at −120°C, exceeding the minimum operating temperature of LSBs by more than twice. This work pioneers a new battery system through the co-design of electrode materials and cell structure, establishing interfacial MFSN that orchestrates quantum-level control over polysulfide reaction pathways and kinetics. This work paves the way for extreme wide-temperature and sustainable high-energy-density battery development, marking a landmark development of battery systems.

## MATERIALS AND METHODS

### Materials preparation

Typically, 3.85 g NH_4_Ac, 1.35 g FeCl_3_·6H_2_O, and 0.40 g trisodium citrate were dissolved in 70 mL ethylene glycol. The mixture was stirred vigorously at 170°C for 1 h, which was then transferred to a stainless-steel autoclave. Then, it was heated at 200°C for 24 h followed by cooling to room temperature. The obtained Fe_3_O_4_ was collected through a magnet, washed four times with ethanol (30 mL), and vacuum dried at 70°C. HTNP (zwitterion-modified Fe_3_O_4_ nanoparticles) was obtained by adding 12.5 mL deionized water, 1.5 mL ammonia, 400 mg Fe_3_O_4_, and 0.8 mL 3-(dimethyl(3-(trimethoxysilyl)propyl)ammonio)propane-1-sulfonate to 50 mL ethanol, stirring vigorously at 60°C for 24 h. The resulting product was separated and washed four times with ethanol and water. Then, HTNP was dried in a vacuum oven at 50°C, and 600 mg G was added to 50 mL NMP (*N*-methyl-2-pyrrolidone) and sonicated for 30 min to form a uniform suspension. At the same time, 300 μL pyrrole and 686 mg APS (ammonium persulfate) were dissolved in 100 and 50 mL deionized water as raw materials, respectively. Then, the obtained G suspension was mixed with pyrrole solution and stored at 4°C. Under continuous stirring, the APS solution was added dropwise to the above mixture of G and pyrrole. The obtained mixture was continuously stirred at 4°C for 36 h. After filtration, GP was obtained by washing with water for three times, washing with ethanol for three times, and drying at 60°C overnight. Then, 30.8 mg 4-mercaptobenzoic acid was dissolved in 5 mL DMF (*N,N*-dimethylformamide), added with 21.6 μL SOCl_2_, and sonicated for 60 min to ensure that the carboxyl group was fully converted into acidic chloride. Then, 36.5 mg 4-dimethylaminopyridine was added to the above solution and sonicated for 15 min to form a raw material solution. At the same time, 60 mg GP and 103.7 μL triethylamine were mixed with 15 mL DMF and sonicated for 90 min to form a uniform suspension. Under continuous stirring, the raw material solution was added dropwise to the GP suspension. The obtained mixture was stirred continuously at 100°C for 36 h. The mixed solution was then cooled to room temperature. After filtration, it was washed with DMF for five times under ultrasound to remove unreacted substance, and then washed with ethanol for two times. Thereafter, 100 mg of the sample, 21 mg of VIPS (3-(1-vinyl-3-imidazolio)propanesulfonate), and an appropriate amount of azobisisobutyronitrile were dissolved in methanol, and ultrasonically mixed for 30 min. The mixed solution was placed in a three-neck flask and stirred at 60°C for 3 h. The samples were washed four times with methanol and dried overnight at 70°C. 150 mg of the dry black powder was taken out, and evenly dispersed in deionized water and stirred for 12 h. It was kept in a refrigerator for 48 h in a static state. GP-ZW-HTNP was obtained after drying.

## Supplementary Material

nwag217_Supplemental_File
