# Supplementary material for: Smart symbiotic lithium–sulfur batteries under extremely low-temperature conditions
Source: Natl Sci Rev. 2026 Apr 9;13(14):nwag217. doi: 10.1093/nsr/nwag217 (PMC13397534; doi:10.1093/nsr/nwag217)
Supplement: nwag217_Supplemental_File [file nwag217_supplemental_file.pdf]

Supplementary Information

Smart symbiotic lithium-sulfur batteries under extremely low-temperature conditions

Runyue Mao[1], Mengfan Pei[1], Xin Jin[1], Dejian Qu[3], Jiangpu Yang[1], Chang Su[1], Shuo Zhuo[1], Naiwen Hu[1], Cijian Zhang[2], Doming Liu[1], Shengming Li[3], Xigao Jian[1,2], Fangyuan Hu[1,*]

[1]School of Materials Science and Engineering, State Key Laboratory of Fine Chemicals. Frontiers Science Center for Smart Materials Oriented Chemical Engineering, Technology Innovation Center of High Performance Resin Materials (Liaoning Province), Dalian University of Technology, Dalian 116024, China.

[2]State Key Laboratory of Fine Chemicals, Frontiers Science Center for Smart Materials Oriented Chemical Engineering, School of Chemical Engineering, Technology Innovation Center of High Performance Resin Materials (Liaoning Province), Dalian University of Technology, Dalian 116024, China.

[3]School of Control Science and Engineering Dalian University of Technology Dalian, China.

*Corresponding author. Email: hufangyuan@dlut.edu.cn (Fangyuan Hu)

Table of contents

Table of contents. ……………………………………………………………………………1

Section 1. Computational Methods……………………………………………………………5

Section 2. Supplementary Note…………………………………………………………………6

Note S1. Electrochemical measurement.…………………………………………………………6

Note S2. Characterizations.………………………………………………………………………8

Note S3. The construction of "smart symbiosis" cell. ……………………………………………11

Note S4. Definition and explanation of terms. ……………………………………………………13

Note S5. Device design and calibration methods. ………………………………………………14

Note S6. Supplementary assembly steps of pouch cells. …………………………………………17

Section 3. Supplementary Figures ………………………………………………………………18

Figure S1. Schematic diagram of material structure. ……………………………………...…18

Figure S2. DRIFTS spectra of different samples. …………………………………………………19

Figure S3. X-ray photoelectron spectroscopy (XPS) of various samples…………………………21

Figure S4. Scanning electron microscopy (SEM) images of various samples. ……………………22

Figure S5. Nitrogen adsorption-desorption curves of different samples. …………………………24

Figure S6. Fourier transform infrared spectroscopy of material synthesis. ………………………25

Figure S7. Transmission electron microscope of material synthesis. …….……………………...26

Figure S8. Electrostatic potential distributions of molecular.……………………………………...27

Figure S9. CV curves at different scan rates. ……………………………………………………...28

Figure S10. Comparison of Tafel curves. ……………………………………………...……29

Figure S11. The Symmetric CV curves..………………………………………………………...…30

Figure S12. The charge-discharge curves at different current densities. …….……………...….31

Figure S13. Molecular structure optimization model. ………...…………………………………32

Figure S14. Simulation of interfacial Li^+ diffusion processes for GP-ZW-HTNP and GP………33

Figure S15. Cycle performance under poor electrolyte conditions. ……………………………….34

Figure S16. Spin density of Fe_3O_4 with/without a magnetic field (Li). ……………………………35

Figure S17. π-electron distribution of different molecular structure..……………………………….36

Figure S18. Radial Wiener-filtered HRTEM of Li_2S growth with MFSN. ………………………37

Figure S19. AICD calculation results of different molecular structure. 38

Figure S20. AICD 2.0 program under magnetic fields. 39

Figure S21. The cycle performance of the pouch cell at 60 °C. 40

Figure S22. Discharge curve (capacity-voltage) of the pouch cell at 25 °C with E/S=2.5.........41

Figure S23. The cycle performance of G@S, GP@S, and GP-ZW-HTNP@S at -20 °C.........42

Figure S24. Electrochemical performance parameters of low-temperature pouch cells. 43

Figure S25. Discharge curves at 60 °C, 25 °C, –40 °C and –120 °C. 44

Figure S26. Discharge curve (capacity-voltage) of the pouch cell at –120 °C.......................45

Figure S27. Theoretical collection coefficients. 46

Figure S28. Comparison of battery performance with/without multi-field synergy at –20°C. 47

Figure S29. Comparison of battery performance with/without multi-field synergy at –40°C. 48

Figure S30. Third-party test report of pouch cell (page 1). 49

Figure S31. Third-party test report of pouch cell (page 2). 50

Figure S32. Third-party test report of pouch cell (page 1)-English version. 51

Figure S33. Third-party test report of pouch cell (page 2)-English version. 52

Figure S34. Comparison of discharge curves with and without MFSN at room temperature. 53

Figure S35. Comparison of discharge curves with and without MFSN at high temperature. 54

Figure S36. The time-voltage curve and time-temperature curve with MFSN at -20 °C. 55

Figure S37. The time-voltage and time-temperature curve (partial view) with MFSN at -20 °C. ...56

Figure S38. The time-voltage curve and time-temperature curve with MFSN at -40 °C. 57

Figure S39. The time-voltage and time-temperature curve (partial view) with MFSN at -40 °C. ...58

Figure S40. The time-voltage curve and time-temperature curve with MFSN at -80 °C............59

Figure S41. The time-voltage and time-temperature curve (partial view) with MFSN at -80 °C. ...60

Figure S42. The time-voltage curve and time-temperature curve with MFSN at -120 °C. 61

Figure S43. The time-voltage and time-temperature curve (partial view) with MFSN at -120 °C...62

Figure S44. The spatial distribution of the magnetic field at -20 °C. 63

Figure S45. The spatial distribution of the magnetic field at -40 °C. 64

Figure S46. The spatial distribution of the magnetic field at -80 °C. 65

Figure S47. The spatial distribution of the magnetic field at -120 °C. 66

Figure S48. Magnetic field intensity at -20 °C. 67

Figure S49. Magnetic field intensity at -40 °C. 68

Figure S50. Magnetic field intensity at -80 °C. 69

Figure S51. Magnetic field intensity at -120 °C. 70

Figure S52. Current waveform, frequency, and other relevant parameters at -20 °C. 71

Figure S53. Current waveform, frequency, and other relevant parameters at -40 °C. 72

Figure S54. Current waveform, frequency, and other relevant parameters at -80 °C. 73

Figure S55. Current waveform, frequency, and other relevant parameters at -120 °C. 74

Figure S56. The discharge curve of the pure Fe_3O_4 with MFSN within 2.8 V to 1.7 V. 75

Figure S57. SEI composition of the lithium metal anode with and without MFSN. 76

Figure S58. XPS (X-ray photoelectron spectroscopy) depth etching of SEI. 77

Figure S59. Li-Cu tests of the lithium anode after cycling with and without a magnetic field. 78

Figure S60. The discharge curve of the pure Fe_3O_4 without MFSN within 2.8 V to 1.7 V. 79

Figure S61. Cycling performance of pouch cells at –20 °C (~2800 hours). 80

Figure S62. Magnetic hysteresis loops of cathode materials in pouch cell with MFSN...............81

Figure S63. Magnetic hysteresis loops of cathode materials in pouch cell with MFSN...............82

Figure S64. The internal temperature rise curves. 83

Section 4. Supplementary Table..85

Table S1. Comparison of electrochemical performance in reported works. 85

Table S2. The small pouch cell detailed parameters at –20 °C.......................................86

Table S3. The small pouch cell detailed parameters at –40 °C.......................................88

Table S4. The small pouch cell detailed parameters at –80 °C.......................................90

Table S5. The small pouch cell detailed parameters at –120 °C.....................................92

Table S6. The large pouch cell detailed parameters at –20 °C.......................................94

Table S7. The large pouch cell detailed parameters at –40 °C.......................................96

Table S8. The large pouch cell detailed parameters at –80 °C.......................................98

Table S9. The large pouch cell detailed parameters at –120 °C. 100

Table S10. Magnetic field activation timeline at -20 °C. 102

Table S11. Magnetic field activation timeline at -40 °C. 107

Table S12. Magnetic field activation timeline at -80 °C. ………………………..…………………108

Table S13. Magnetic field activation timeline at -120 °C..………………………..…………………110

Table S14. The detailed parameters of the device..…………………….……………………………112

Section 5. Supplementary Movie. ………………………………………………………………113

Movie. S1. Real-time driving of the UAV under dr ice conditions. …………………………….113

Supplementary References ……………………………………………………………………114

Section 1. Computational Methods

Computational methods

Gromacs (2023.02 version) was used to perform MD simulation. According to the stoichiometric ratio and experimental density, different molecules were added in the simulation box (8×8×8 nm). In this study inter- and intramolecular interactions were described by the OPLS-AA force field. LigParGen server is used to generate automatically parameters of all molecules. The simulation began with a 2-ns NPT run at 500 K, and was subsequently followed by a 3-ns NPT annealing step where the system's temperature was progressively reduced from 330 K to ambient temperature (298 K). Then, MD simulation was carried out at 573K. A homogeneous single-phase solution was prepared in this way as the initial configuration. The pressure and temperature were controlled by Berendsen barostat and Nose-Hoover thermostat, respectively. 1.0 fs was set as the time step in all simulations. VMD is used to visualized snapshots of the MD trajectory. Gaussian 16 software is used to perform Density Functional Theory (DFT) calculations. All molecular structures were optimized at the B3LYP/6-31+G(d, p) level, and the corresponding energies were obtained at the M062X/def2TZVP level. To better approximate real experimental conditions, the self-consistent reaction field (SCRF) method based on the SMD model to describe the electrolyte solvent (DOL:DME=1:1). We obtained π-electron characteristics and IRI interactions from the Multiwfn software[1] and visualized them using VMD software[2]. DFT calculations were performed with the Gaussian16, A.03 software package. The geometry optimization calculations were performed using the B3LYP functional[3] with the Becke-Johnson damping scheme (D3BJ)[4], and the 6-31G* basis set[5] was utilized for all atoms. The magnetic induction current diagram was completed by the AICD 2.0 program[6]. Geometric optimization calculations were performed using the B3LYP functional with Becke-Johnson (D3BJ) correction term[3, 4]. All atoms use the 6-31G * basis set[5]. The magnetic induction current diagram is generated by the AICD 2.0 program[6].

Section 2. Supplementary Note

Note S1. Electrochemical measurement

CR2032 coin cell was used in some electrochemical measurements of samples. The active materials, polyvinylidene fluoride (PVDF) binder and graphene were mixed in N-methyl-2-pyrrolidone (NMP) solvent in a mass ratio of 8:1:1. The sulfur loadings range of coin cell is 1.0 and 1.5 mg cm^{-2}. A DOL/DME solution containing 2.0 wt% $LiNO_3$ and 1.0 mol L^{-1} LiTFSI was employed as the electrolyte, with celgard 2400 as separator, lithium foil as the anode, and the above sulfur electrodes as the cathode of cell. In addition, for electrochemical tests under poor electrolyte conditions and relatively high areal sulfur loadings, a electrolyte/sulfur ratio around 2.5 mL g^{-1} and a sulfur loading range of 4.0~9.0 mg cm^{-2} was used. EIS and CV tests were performed using a Biologic VMP-3 multichannel workstation with a voltage window (1.5-3 V) for CV and 10 mHz-100 kHz for EIS. The in-situ EIS tests are performed by discharging the batteries to 2.5 V, 2.3 V, 2.2 V, 2.1 V, 2.0 V, 1.9 V, 1.8 V and 1.7 V, respectively, and performing the EIS test after discharging the batteries to each of the corresponding voltages. After each EIS test, it will continue to discharge to the next voltage node and then conduct EIS test. The evaluation of Li^+ diffusion properties through the Randles-Sevick equation ($I=26900\times n^{1.5}\times A\times D^{0.5}\times C\times v^{0.5}$). The galvanostatic charge-discharge tests were carried out in the 1.7-2.8V voltage range by LAND CT2001A instruments. The sulfur and Li_2S were mixed with a molar ratio of 5:1 to prepare a 0.2 M Li_2S_6 catholyte in DOL:DME=1:1 with 1 M LiTFSI and 1% wt $LiNO_3$. Li_2S_6 catholyte was used for symmetric CV tests. The potential range is -1 to 1 V. The sulfur and Li_2S were mixed with a molar ratio of 7:1 to prepare a 0.2 M Li_2S_8 electrolyte in DOL:DME=1:1 with 1 M LiTFSI and 2% wt $LiNO_3$. The solution was stirring at 80 °C for 36 h. 0.2 M Li_2S_8 electrolyte was used for Li_2S nucleation tests. In Li_2S nucleation tests, cells were first galvanostatically discharged to 2.09 V at 0.112 mA, and then potentiostatically discharged at 2.08 V until the current gradually reduced to 0.01 mA. The testing of pouch cells at −120 °C is carried out in an ultra-low temperature refrigerator. The low-temperature tests of pouch cells are generally conducted in near-vacuum environments. When the temperature of the refrigerator is controlled at around −120 °C, the whole device is placed in the refrigerator for electrochemical performance testing. The test of soft pack battery at 60 °C is carried out in a blower oven, when the temperature of the oven is controlled at about 60 °C, the whole

device is placed in the oven for electrochemical performance test. The assembly details of the pouch cell refer to *Fabrication of pouch cell*. For pouch cells with and without MFSN, after being discharged at 0.1 C to 2.3 V, 2.2 V, 2.1 V, 2.0 V, 1.9 V, and 1.7 V respectively, the pouch cells were cut open in a glove box, and part of the positive electrode was taken out for Raman spectroscopy testing. The model of the confocal Raman microspectrometer is Invia Qontor from the United Kingdom. For pouch cells with and without MFSN, after being discharged at 0.1 C to the late stage of the first platform, the early stage of the second platform, and the late stage of the second platform respectively, the pouch cells were cut open in a glove box, and part of the positive electrode was taken out for synchrotron radiation testing. The synchrotron radiation test was performed using the synchrotron radiation instrument at the Shanghai Synchrotron Radiation Facility. For pouch cells with and without MFSN, after being discharged at 0.1 C to 2.3 V, 2.2 V, 2.1 V, 2.0 V, 1.9 V, 1.8 V, 1.7 V and then charged to 2.3 V, 2.4 V, and 2.5 V respectively, the pouch cells were cut open in a glove box, and part of the positive electrode was taken out. The positive electrode was dried and scraped off for magnetization measurements. The model of the testing instrument is LakeShore 7404 from the United States.

Note S2. Characterizations

The overall electrochemical performance of GP-ZW-HTNP-based lithium-sulfur batteries was evaluated by means of Ah-grade pouch cells. The active materials, polyvinylidene fluoride (PVDF) binder and graphene were mixed in N-methyl-2-pyrrolidone (NMP) solvent in a mass ratio of 8.5:0.75:0.75. A DOL/DME solution containing 1.0 wt% $LiNO_3$ and 0.5 mol L^{-1} LiTFSI was employed as the electrolyte, with celgard 2400 as separator. In a glove box, double-sided coated positive plates (with carbon coated aluminium foil as a collector), double-sided lithium-copper composite negative plates (with copper as a collector), and Celgard polypropylene diaphragms were stacked layer-by-layer to form the cell. The loading was around ~4.5 mg cm^{-2} on the positive side and 9 mg cm^{-2} on both sides. One side of the lug is heat-sealed at 200 °C, followed by the other two sides, leaving the last side. The last side is filled with electrolyte, vacuumed and heat sealed. The amount of electrolyte was determined by the mass of sulfur, and the E/S ratio was 2.5. The sulfur loading of the ~20Ah pouch cell is ~5.5 mg cm^{-2}, the E/S is 2, the number of positive plates is 22, and the number of negative plates is 23. The pouch cells were subjected to electrochemical tests after standing.

The fibre optic stress sensor is embedded in the electrode paste and the dried electrodes are assembled into a pouch cell. The optical fibre is connected to a fibre optic demodulator and the pouch cell is connected to a electrochemical test device. Microstress and electrical signals were recorded simultaneously when the cell was started. The pouch cell charge-discharge voltage range is 1.7-2.6 V and the current density is 0.1 C.

A specially made all-copper grid was placed on the positive electrode side of the pouch cell. During liquid injection, the cell was fixed to ensure close contact between the copper grid and the positive electrode. After the assembly of the pouch cell was completed, it was left to stand for more than 8 hours. Then, under both conditions with and without MFSN, it was discharged at a current density of 0.1 C to different discharge stages, with a final discharge voltage of 1.7 V. During the discharge process, the pouch cell was clamped with a fixture to ensure good contact. After the discharge was completed, the pouch cell was cut open and the copper grid was taken out. The copper grid was sealed in a sealed bag. The model of the cryo-transmission electron microscope is American FEI Talos F200C, and liquid nitrogen was used for cooling.

The assembly details of the pouch cell refer to *Fabrication of pouch cell*. After the pouch cell was assembled, it was left to stand for more than 8 hours. Then, under both conditions with and without MFSN, it was discharged at a current density of 0.1 C to a cut-off voltage of 1.7 V. For pouch cells with and without MFSN, they were discharged at 0.1 C to 1.7 V. The pouch cells were cut open in a glove box, and part of the positive electrode was taken out. The positive electrode was dried and scraped off, then ultrasonicated in an ultrasonic machine for 30 minutes. After ultrasonication, Fe_3O_4 in the positive electrode was collected using a magnet. The collected Fe_3O_4 was then dispersed in deionized water, and the dispersed solution was dropped onto a double-sided carbon-coated copper grid using a pipette. After drying, the copper grid was folded and fixed, and then sealed in a sealed bag. The model of the Spherical Aberration-Corrected Transmission Electron Microscope used is JEM-ARM300F2 from Japan.

The sensing part of the micro sensor is placed on the positive electrode plate to monitor the internal temperature of the positive electrode in real time. The other side of the micro sensor is connected to the chip. One end of the chip takes over the signal from the sensor, and the other end issues instructions to the magnetic field device to regulate the magnetic field power. Thus, an intelligent device for real-time feedback and regulation is constructed.

The pouch cell is placed in a dry ice tank and connected to the drone. After waiting for 20 minutes, the magnetic field is started, and the temperature change of the cell surface is monitored by a thermal imager. The whole process of driving the drone by the pouch cell is monitored in real time through the camera.

Advanced Fourier transform infrared spectrometer 6700 is used to characterize Fourier transform infrared spectroscopy (FTIR). In order to examine the structure of the samples, the transmission electron microscopy (TEM) was performed through Eindhoven. In situ FTIR spectroscopy was carried out using a Thermo Fisher Scientific Nicolet iS50. The material was placed in a −120 °C and the temperature was increased at a rate of 2 °C/min from −120 °C. The temperature was recorded at −120 °C, −100 °C, −80 °C, −40 °C, −20 °C, 20 °C, 40 °C, 60 °C and 80 °C, respectively. The FTIR spectra were recorded at −120 °C, −100 °C, −80 °C, −40 °C, −20 °C, 20 °C, 40 °C, 60 °C and 80 °C, respectively. The hysteresis return line test (VSM) was performed with LakeShore 7404 equipment in the United States. The system has a magnetic field strength of ±9 T, a scan rate of 1-

200 Gauss/s, and a magnetic field resolution of 0.02 mT.

Note S3. The details of cells-sensors-chip-fields intelligent device construction

We use a miniature sensor T-0.05, which has a filament diameter of 0.05 mm and can convert temperature signals into voltage signals in real time. Firstly, T-0.05 was laid flat on the collector, and the anode slurry was scraped on it and dried at 60 °C. This was done to more accurately monitor the real-time temperature inside the positive electrode of the battery so that the chip can be regulated according to the specific situation. The chip was developed by us in collaboration with Senior Engineer Shengming Li from the School of Future Science and Technology, Dalian University of Technology, and it can analyse the voltage signals coming out of the miniature sensors in real time. One end of the sensor is connected to the chip and the other end is buried inside the electrode. The chip is directly attached to the magnetic field device and adjusts the power of the magnetic field device by issuing commands. The magnetic field device affects the positive electrode material inside the battery by creating an alternating magnetic field. The cathode material, upon responding to the alternating magnetic field, generates microscopic thermal and intermolecular electric fields. Meanwhile, the spin electron transition at the interface reaction site can be modulated at the quantum level by the magnetic field. At this point, the thermal field signal can be sensed by the T-0.05 and further transmitted out of the battery to be analysed by the chip to further regulate the physical field inside the battery. A module consists of more than 8 pouch cells, and the magnetic field emitter is located in the center. The internal temperature of each cell is monitored by microsensors and the relevant information is given to the chip. The chip and the emitter are supplied by the cells through wires. The output energy of the module is provided by pouch cells connected in series and parallel. Thus, the in-situ response−dynamic monitoring−real-time feedback intelligent integrated battery device is successfully constructed.

According to the test results, the total energy consumption during the discharge process of a single pouch cell at –20 °C is 9.32 Wh, while the energy of the cell itself is 48.2 Wh. Based on the total module mass and subtracting the energy consumption, the energy density is calculated to be 456.9 Wh kg^{-1}. At –40 °C, the total energy consumption of a single pouch cell during discharge is 14.136 Wh, while the energy of the cell itself is 51.38 Wh. Based on the total module mass and subtracting the energy consumption, the energy density is calculated to be 438.19 Wh kg^{-1}. At –80 °C, the total energy consumption of a single pouch cell during discharge is 28.71 Wh, while the

energy of the cell itself is 55.74 Wh. Based on the total module mass and subtracting the energy consumption, the energy density is calculated to be 314.9 Wh kg^{-1}. At –120 °C, the total energy consumption of a single pouch cell during discharge is 62.83 Wh, while the energy of the cell itself is 57.8 Wh. From the perspective of large-scale application, this mainly involves two scenarios: one is the application in extreme cold environments (0 to –40 °C) in daily life on the ground, and the other is the power drive for instruments in the near-space region (–60 to –90 °C). The reason these two aspects are involved is that in the near-space region, the air is thin and the temperature is extremely low, resulting in slower heat conduction, which significantly reduces the energy consumption required for the magnetic field. For daily use on the ground, the application of this solution requires pairing it with a lightweight and simple insulation shell.

Note S4. Definition and explanation of terms

Smart symbiosis: The term "Smart symbiosis" in this work comprises two key aspects: Smart and Symbiosis. Smart: Refers to components like the chip, which enable autonomous feedback and regulation. This grants the overall structure intelligent characteristics—a system capable of self-feedback, self-regulation, and adaptation to external changes through sensing and interaction. Symbiosis: While typically biological, this term is adopted to illustrate that the assembled battery module functions like an organism thriving under extreme cold. Specifically, it denotes the symbiotic relationship between the magnetic field and the battery: The magnetic field ensures stable battery operation at ultra-low temperatures and stimulates the battery to generate significantly higher capacity than at room temperature. The battery supplies power to maintain the magnetic field and chip operation. The chip regulates magnetic field power based on sensor signals. Electrode materials respond to this synergy, significantly accelerating polysulfide kinetics and generating novel additional capacity. This additional capacity replenishes the energy consumed by the magnetic field and chip, enabling highly efficient and stable ultra-low temperature operation. Without the magnetic field, the battery is nearly inoperable at ultra-low temperatures (<–40 °C). Conversely, without the battery supplying power, the magnetic field cannot provide feedback or regulation.

Multi-field synergy: In this paper, "multi-field synergy" is defined as the dynamic enhancement facilitated by the collaboration of three primary fields—the magnetic field, the thermal field generated by magnetic field-excited nanoparticles, and the electric field arising from rapid electron transport in both magnetic field-excited nanoparticles and conductive polymers—along with the responsive "secondary fields." The thermal field produced by magnetic field-excited nanoparticles primarily ensures that the temperature at the cathode conversion sites remains within a range suitable for polysulfide conversion reactions while enabling rapid Li^+ transport. The electric field, resulting from fast electron transport in magnetic field-excited nanoparticles and conductive polymers, mainly guarantees electron supply during the multi-electron polysulfide conversion reaction. It is the synergy of these three fields that enables the polysulfide conversion ratio to exceed the theoretical value and ensures stable operation under ultralow temperatures.

Note S5. Device design and calibration methods

The magnetic field device uses a single-switch parallel resonant converter, with a fixed switching frequency of ~72 kHz. As the excitation voltage and coil load change, the transferred energy also varies accordingly, which is used for driving the magnetic field. The driving circuit of the magnetic field device is as follows:

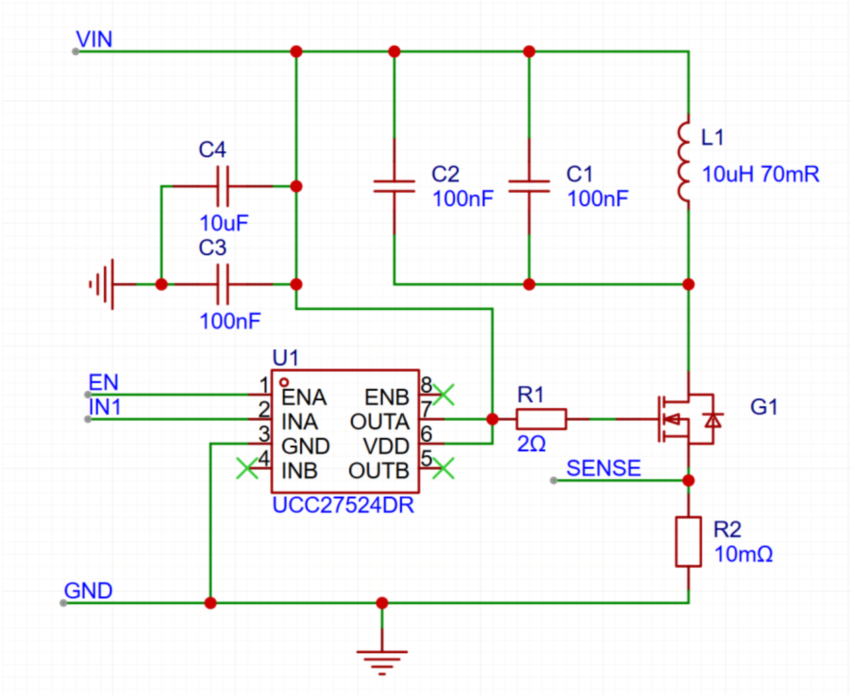

Herein, L1 is the transmitting coil, which forms a resonant circuit with C1 and C2 to amplify the voltage and current, generating a sufficient driving magnetic field.

(Input Specifications) This magnetic field generating device supports a DC voltage input ranging from 7 V to 16 V. The excitation source can be regulated by adjusting the input voltage, thereby modifying the power in the same medium to adapt to temperature control experiments under different temperatures.

(Output Specifications) The magnetic field generating device supports a maximum coil current of 35 A peak-to-peak to produce the excitation magnetic field. The actual excitation current is determined by the input voltage/current settings and the load characteristics.

(Calibration Method) During the experiments, the magnetic field generating device requires no calibration. The temperature measurement calibration is performed using a controlled and stable external ambient temperature as the reference benchmark.

The temperature acquisition circuit is as follows: it employs the MAX31856 chip, which integrates a temperature reference to enable high-precision thermocouple temperature sampling. It supports multiple thermocouple types and can work with an MCU for temperature calibration, thereby enhancing the accuracy of temperature measurements.

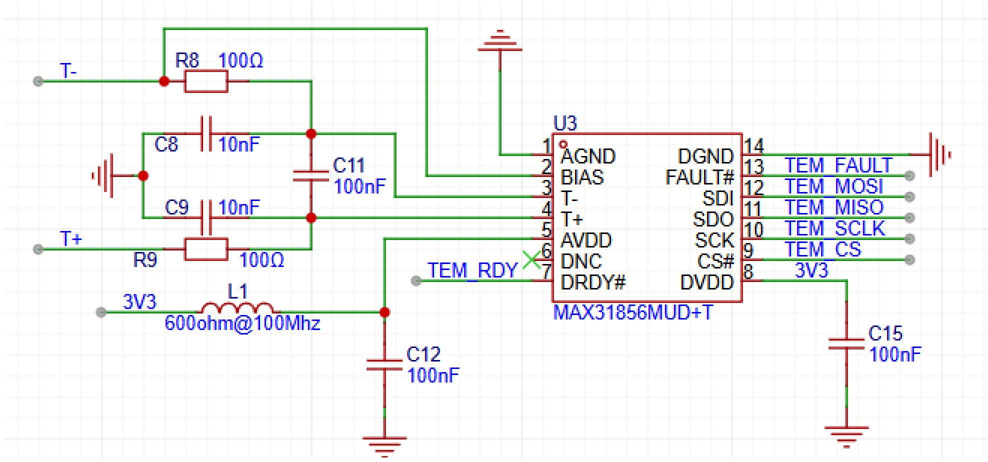

The main MCU uses the STM32G071GBU6 to drive the MAX31856 for temperature sampling, thereby controlling the magnetic field device to achieve closed-loop temperature control.

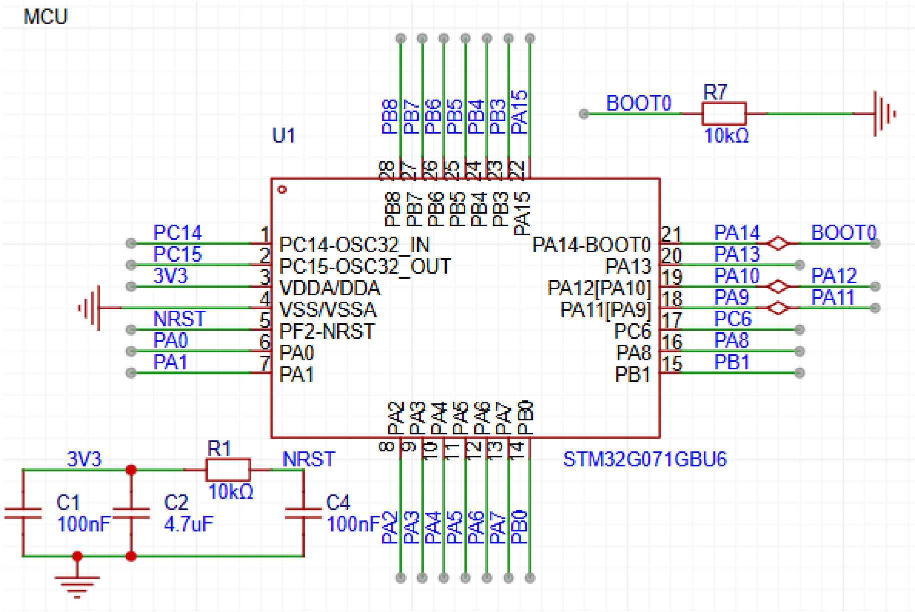

The specific parameters of the magnetic field are determined through simulation based on the input voltage and current, which correspond to the power.

Increasing the input voltage can enhance the coil excitation voltage, thereby increasing the voltage amplitude of the magnetic field generating coil.

$$V_{coil} = I_{coil} \times Z = I_{coil} \times \sqrt{{R_{eq}}^2 + (2\pi f L)^2}$$

In the same medium, the coil characteristics remain unchanged, allowing for greater coil current to be generated, which in turn produces a stronger magnetic field intensity.

V_{coil} is the voltage across the coil, I_{coil} is the coil current, and R_{eq} is the equivalent resistance of the coil. This is not the DC resistance of the coil, but rather an AC equivalent resistance incorporating the electromagnetic coupling effect, which depends on: the electromagnetic properties

of the material: Resistivity (ρ); Relative permeability (μ_r); Operating frequency (f); Degree of coupling between the coil and the material (gap); Geometric dimensions of the material. Under constant other conditions, a stronger magnetic field results in higher heating power and a more pronounced kinetic promotion effect. An increase in the alternating current in the coil generates a larger alternating magnetic field, thereby enhancing both the heating and kinetic promotion effects.

Based on the principles described above, different input voltages are set to generate magnetic fields of varying intensities, thereby accommodating experiments at different temperatures. It is worth noting that the difference between the input current and voltage and those measured in the resonant circuit is caused by the resonant circuit, primarily aimed at generating a sufficient magnetic field. The specific settings are as follows. The magnetic field frequency is 72.46 kHz at -20 °C, 73.53 kHz at -40 °C, 74.63 kHz at -80 °C, and 75.76 kHz at -120 °C.

	Voltage	Current	Magnetic field intensity	Magnetic frequency	Peak to peak current
-20	8 V	0.365 A	~93.9 mT	72.46 kHz	9.06 A
-40	8.5 V	0.438 A	~124 mT	73.53 kHz	9.99 A
-80	9.2 V	0.580 A	~166 mT	74.63 kHz	13.12 A
-120	9.9 V	1.002 A	~274 mT	75.76 kHz	17.19 A

Note S6. Supplementary assembly steps of pouch cells

In the assembly of the 20 Ah pouch cell, we used a less dense electrolyte of 0.5 M LiTFSI in DOL:DME = 1:2, while the small-capacity pouch cell employed the more common electrolyte of 1 M LiTFSI in DOL:DME = 1:1. In pursuit of higher energy density, the 20 Ah pouch cell therefore employs a copper-lithium composite strip measuring 100 μm+5 μm+100 μm. Due to the high density of copper, this results in a significant reduction in mass. Optimizations were carried out respectively from the electrolyte and lithium anode directions, thereby reducing mass to achieve higher energy density while ensuring other performance metrics, in order to meet the required specifications. Regarding the sulfur content, the cathode slurry is stirred in a large-scale stirring tank with a ratio of 8.5:0.75:0.75. Regarding the areal loading of the cathode, the large pouch cells employ automatic coating via a double-sided coater, while the small pouch cells use manual coating. Consequently, the areal loading of the cathode electrode for large pouch cells is twice that of the small pouch cells. In addition, the electrodes of the large pouch cells were processed using rolling/calendering technology, while the small pouch cells were not subjected to this process.

Section 3. Supplementary Figures

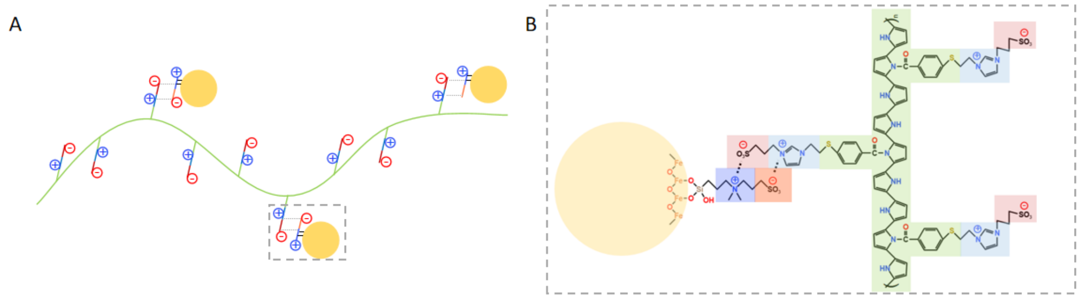

Figure S1: Schematic diagram of material structure.

Microstructure diagram (a) and molecular structure diagram

(b) of GP-ZW-HTNP material.

In Supplementary Fig. 1a, the green line is the main chain of the conjugated structure, the positive and negative sign zwitterions, and the yellow circle part represents the Fe_3O_4 nanoparticles. The Supplementary Fig. 1b is a schematic diagram of the enlarged molecular structure.

Supplemental Experimental Procedures.

Firstly, polypyrrole was integrated on graphene, and 3-(1-Vinyl-3-imidazolio) propanesulfonate (VIPS) was grafted onto polypyrrole by 4-mercaptobenzoic acid intermediate to obtain GP-ZW. Then 3-(Dimethyl(3-(trimethoxysilyl)propyl)ammonio)propane-1-sulfonate (SBS) was grafted onto Fe_3O_4 particles to obtain HTNP, and zwitterionic functional groups were uniformly distributed on the surface. Subsequently, HTNP was dispersed and homogeneously mixed with GP-ZW in solution, and finally GP-ZW-HTNP was obtained. There is a strong anion-cation interaction between VIPS and SBS zwitterions, which makes Fe_3O_4 clusters evenly distributed on the conductive segments and inhibits the agglomeration of Fe_3O_4.

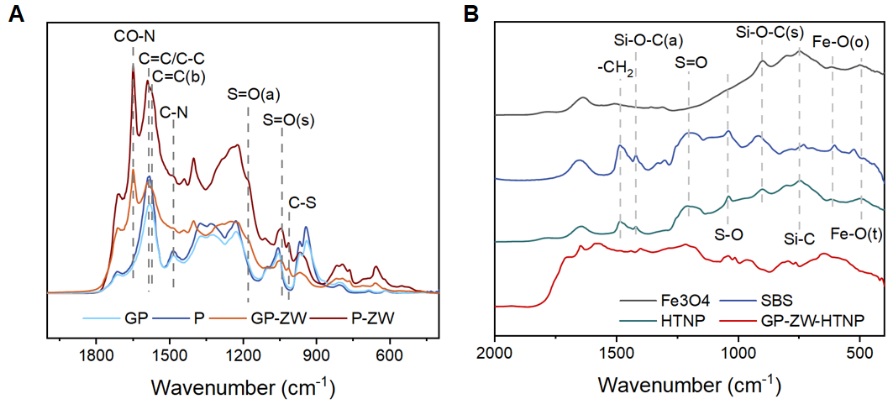

Figure S2. Diffuse reflectance fourier-transform infrared spectroscopic (DRIFTS) spectra of different samples.

(A) DRIFTS spectra of GP, P, GP-ZW and P-ZW.

(B) DRIFTS spectra of Fe_3O_4, SBS, HTNP and GP-ZW-HTNP.

Supplemental Experimental Procedures.

Since the materials generally appear black, we chose to analyze each step of the synthesis in detail using diffuse reflectance Fourier-transform infrared spectroscopy (DRIFTS). As shown in Figure S2A, the C-N peak at 1484 cm^{-1} originates from the pyrrole structure on the polypyrrole (PPy) chain. This peak remains after grafting 4-mercaptopropionic acid (MPA) and VIPS, proving that the grafting process did not disrupt the main chain structure of PPy. The appearance of the C=O-N peak at 1649 cm^{-1} is attributed to the successful reaction between the -COOH of MPA and the -NH on PPy. The peaks at 1042 cm^{-1} and 1181 cm^{-1} correspond to the symmetric and asymmetric stretching vibrations of S=O in VIPS, respectively. Meanwhile, the peaks at 1591 cm^{-1} (C=C/C-C) and 1572 cm^{-1} (C=C (b)) arise from the π-π conjugation in the PPy conjugated chain and the large π bond on the benzene ring, respectively. The disappearance of the C=C peak of VIPS at 1620 cm^{-1} and the emergence of the two S=O peaks confirm the successful grafting of VIPS onto the PPy chain via the reaction between the C=C of VIPS and the -SH of MPA. Additionally, the appearance of the C-S bond at 1012 cm^{-1} further supports this conclusion. As shown in Figure S2B, the peaks at 614 cm^{-1} and 491 cm^{-1} correspond to the Fe-O bonds in the octahedral and tetrahedral positions of Fe_3O_4, respectively. The peak at 1042 cm^{-1} corresponds to Si-O-C in SBS, while the peak at 1418 cm^{-1} represents the characteristic N^+-CH_3 vibration of SBS. In GP-ZW-HTNP, this peak shifts to a higher wavenumber due to the formation of cation-anion pairing interactions between the two zwitterionic

groups. The peak at 1483 cm^{-1} corresponds to the C-H bending vibration of O-CH_3 in SBS. After repeated washing to remove unreacted SBS, HTNP still exhibits distinct Si-O-C and N^+-CH_3 peaks, confirming the successful grafting of SBS onto the surface of Fe_3O_4.

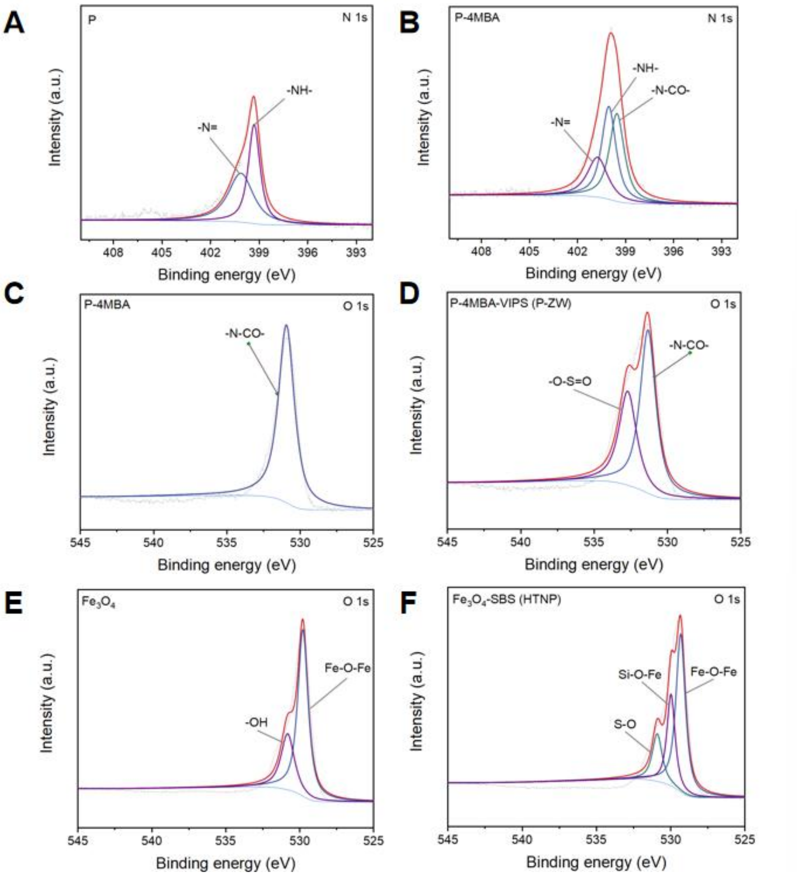

Figure S3. X-ray photoelectron spectroscopy (XPS) of various samples

(A) and (B) XPS spectra (N 1s) of P and P-4MBA.

(C) and (D) XPS spectra (O 1s) of P-4MBA and P-4MBA-VIPS (P-ZW).

(E) and (F) XPS spectra (O 1s) of Fe_3O_4 and Fe_3O_4-SBS (HTNP).

Supplemental Experimental Procedures.

As shown in the Figure S3, Figure S3A-B display the SEM images of Fe_3O_4, while Figure S3C shows the SEM image of HTNP. Figures C-E present the SEM images of GP-ZW-HTNP. It can be observed that the particles are dispersed on polypyrrole (PPy) and graphene through cation-anion pairing interactions between zwitterionic groups.

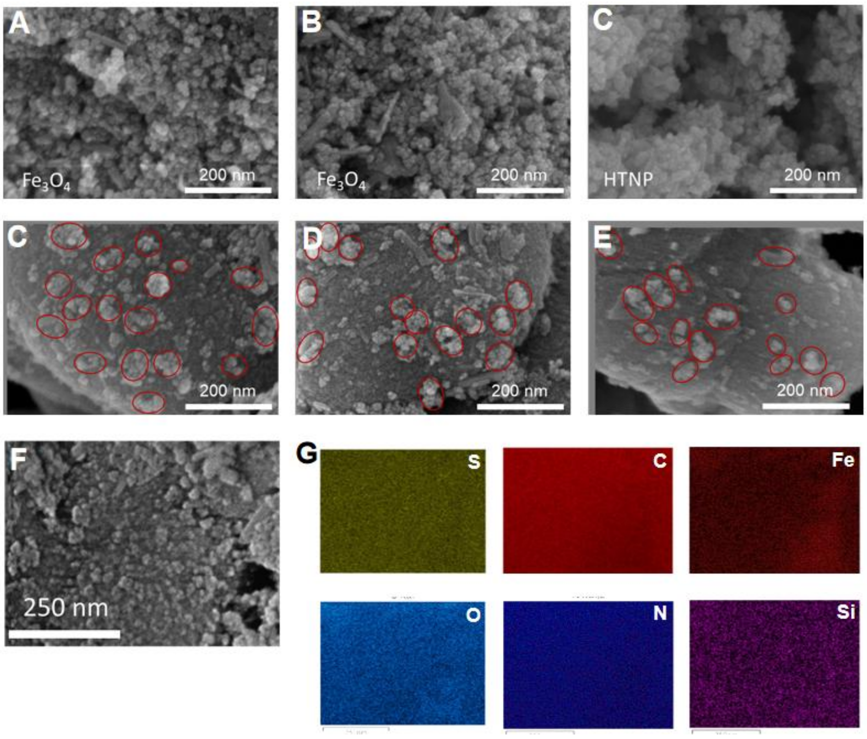

Figure S4. Scanning electron microscopy (SEM) images of various samples.

(A-B) SEM images of Fe_3O_4.

(C) SEM images of HTNP.

(D-E) SEM images of GP-ZW-HTNP.

(F)and (G) EDS images of GP-ZW-HTNP.

Supplemental Experimental Procedures.

The nitrogen adsorption-desorption curves are shown in the Figure S4. The specific surface area of Fe_3O_4 nanoparticles is 121.1269845 cm^3 g^{-1} (STP), while that of HTNP is 177.7336 cm^3 g^{-1} (STP), and GP-ZW-HTNP exhibits a higher specific surface area of 246.73 cm^3 g^{-1} (STP). The increased surface area of HTNP compared to Fe_3O_4 is attributed to the grafting of zwitterionic SBS on its surface, which helps suppress the agglomeration of Fe_3O_4 nanoparticles. GP-ZW-HTNP shows an even higher surface area than HTNP because polypyrrole (PPy) grows uniformly on graphene, and the zwitterionic VIPS grafted onto PPy interacts with SBS through cation-anion pairing, facilitating the dispersion of Fe_3O_4 nanoparticles on the graphene and PPy surfaces. As shown in Figure B, the specific surface area of P (pristine polypyrrole) is 11.59 cm^3 g^{-1} (STP), while P-ZW (zwitterion-modified polypyrrole) reaches 79.62 cm^3 g^{-1} (STP), and GP-ZW (graphene-supported zwitterionic polypyrrole) achieves 224.85 cm^3 g^{-1} (STP). Although polypyrrole and its modified forms alone

exhibit low surface areas, coating them onto graphene significantly enhances the material's specific surface area. These results further confirm the success of the synthesis.

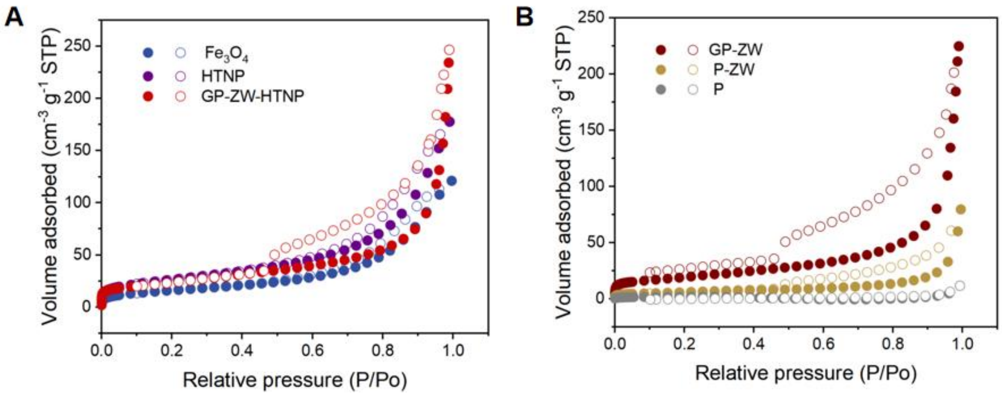

Figure S5. Nitrogen adsorption-desorption curves of different samples.

(A) Nitrogen adsorption-desorption curves of Fe_3O_4, HTNP, GP-ZW-HTNP.

(B) Nitrogen adsorption-desorption curves of GP-ZW, P-ZW, P.

Supplemental Experimental Procedures.

The nitrogen adsorption-desorption curves are shown in the Figure S5. The specific surface area of Fe_3O_4 nanoparticles is 121.1269845 $cm^3\ g^{-1}$ (STP), while that of HTNP is 177.7336 $cm^3\ g^{-1}$ (STP), and GP-ZW-HTNP exhibits a higher specific surface area of 246.73 $cm^3\ g^{-1}$ (STP). The increased surface area of HTNP compared to Fe_3O_4 is attributed to the grafting of zwitterionic SBS on its surface, which helps suppress the agglomeration of Fe_3O_4 nanoparticles. GP-ZW-HTNP shows an even higher surface area than HTNP because polypyrrole (PPy) grows uniformly on graphene, and the zwitterionic VIPS grafted onto PPy interacts with SBS through cation-anion pairing, facilitating the dispersion of Fe_3O_4 nanoparticles on the graphene and PPy surfaces. As shown in Figure B, the specific surface area of P (pristine polypyrrole) is 11.59 $cm^3\ g^{-1}$ (STP), while P-ZW (zwitterion-modified polypyrrole) reaches 79.62 $cm^3\ g^{-1}$ (STP), and GP-ZW (graphene-supported zwitterionic polypyrrole) achieves 224.85 $cm^3\ g^{-1}$ (STP). Although polypyrrole and its modified forms alone exhibit low surface areas, coating them onto graphene significantly enhances the material's specific surface area. These results further confirm the success of the synthesis.

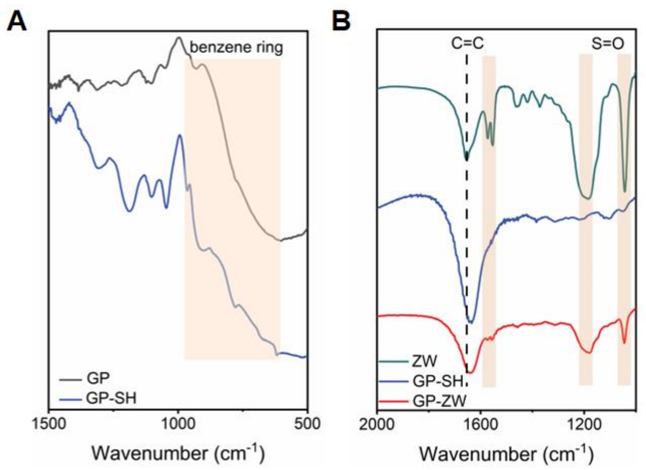

Figure S6. Fourier transform infrared spectroscopy spectra of material synthesis.

(a) Fourier transform infrared spectroscopy (FT-IR) of GP and GP-SH.

(b) FT-IR of GP-ZW, GP-SH and ZW. GP is a composite material of graphene and polypyrrole. GP-SH was obtained by modifying 4-mercaptobenzoic acid on conjugated segment.

Supplemental Experimental Procedures.

The four peaks appearing in the 500 to 1000 cm^{-1} interval originate from the four C-H on the benzene ring. ZW is the zwitterion—VIPS. The disappearance of C=C in ZW is due to the reaction of -SH with C=C, grafting VIPS onto GP-ZW. The peaks around 1080 cm^{-1} and 1190 cm^{-1} are assigned to S=O symmetric and S=O asymmetric stretching adsorption peaks, respectively. The appearance of these peaks in GP-ZW further confirms the successful grafting of VIPS.

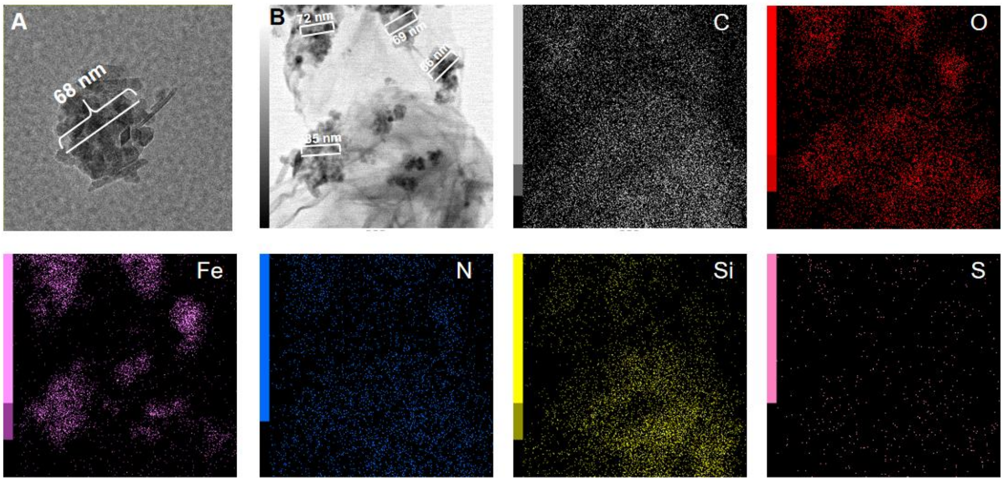

Figure S7. Transmission electron microscope of material synthesis.

(a) Transmission electron microscope (TEM) images of a single HTNP cluster.

(b) TEM images of GP-ZW-HTNP and the corresponding Energy dispersive spectrometer (EDS).

Supplemental Experimental Procedures.

The C element is mainly derived from graphene. The O element is mainly derived from SO_3^- and HTNP of VIPS. Fe is mainly derived from HTNP. N mainly comes from the conjugated backbone. Si mainly comes from SBS. S mainly comes from SO_3^-. The results confirm uniform Fe_3O_4 distribution on graphene and the polymer, which was due to the anion-cation interaction between VIPS and SB.

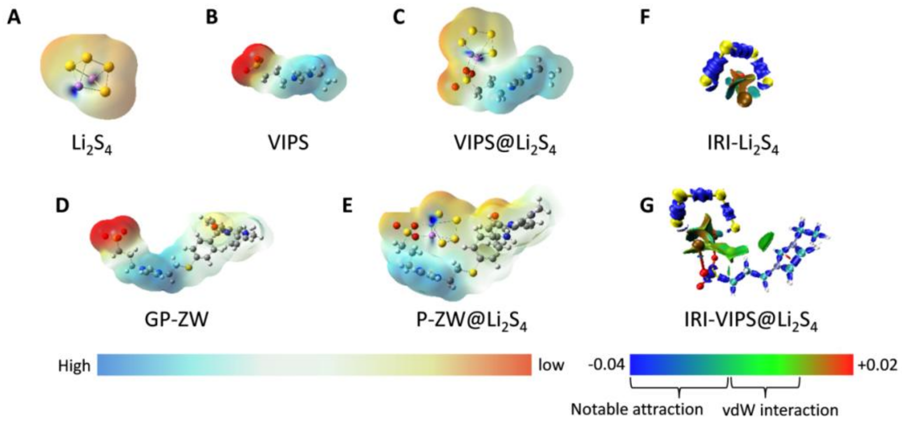

Figure S8. Electrostatic potential distributions of molecular. Electrostatic potential distributions of (a) Li_2S_4, (b) VIPS, (d) P-ZW and (e) P-ZW@Li_2S_4 configuration. Interaction region indicator (IRI) calculations of (f) IRI-Li_2S_4 and (g) IRI-VIPS@Li_2S_4.

Supplemental Experimental Procedures.

The blue part is the positive potential part and the red part is the negative potential part. S_4^{2-} is the negative potential part and Li^+ is the positive potential part in Li_2S_4. Imidazole ring is the positive potential part and SO_3^- is the negative potential part in VIPS. After conformational optimisation, Li_2S_4 combines with VIPS to form two anionic pairs, Li^+--SO_3^- and S_4^{2-}--imidazole ring, respectively. This binding is caused by the pairing of the positive potential part and the negative potential part with each other.The same pairing configuration in IRI calculations also confirms the existence of electrostatic interactions between S_4^{2-} and imidazolium ring that are stronger than the normal van der Waals forces.

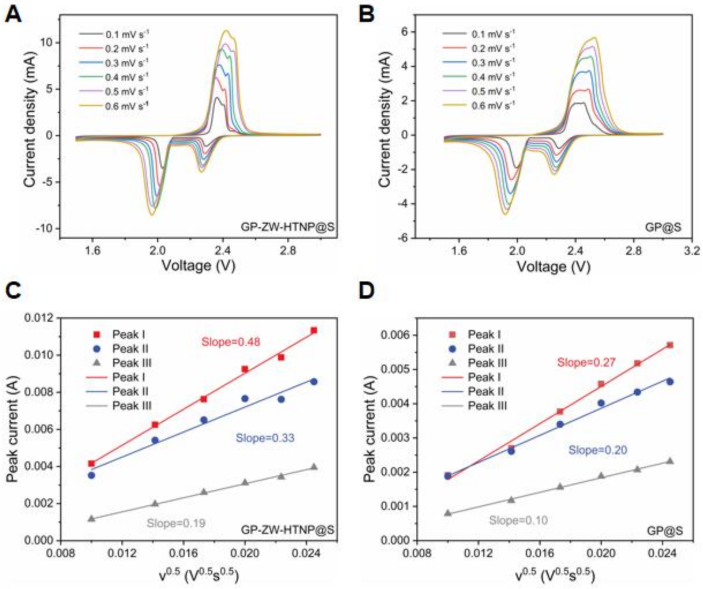

Figure S9. CV curves at different scan rates.

(a) CV of GP-ZW-HTNP@S and

(b) CV of GP@S at different scanning rates (0.1 mV s^{-1}, 0.2 mV s^{-1}, 0.3 mV s^{-1}, 0.4 mV s^{-1}, 0.5 mV $s^{-1}$and 0.6 mV s^{-1}), and corresponding Randles–Sevcik plot of peak current versus square root of scan rate (c and d).

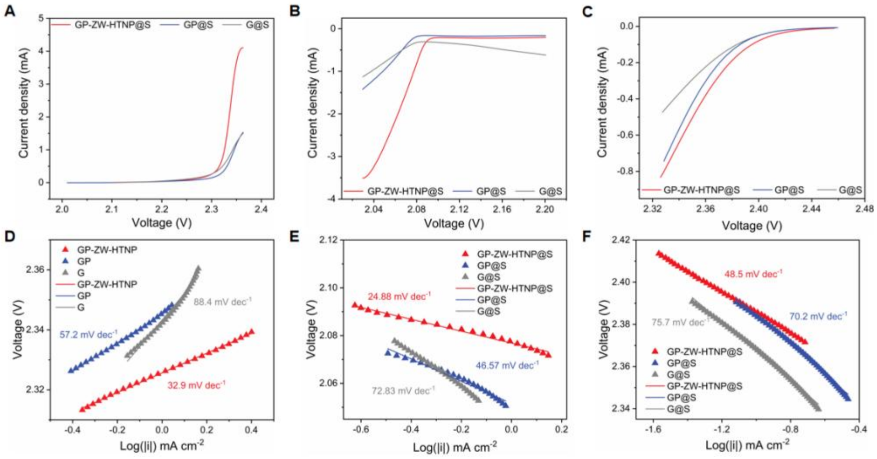

Figure S10. Comparison of Tafel curves.

(a-c) Constant potential polarisation curves of peaks A, B and C of GP-ZW-HTNP@S, GP@S and G@S.

(d-f) Tafel curves corresponding to peaks A, B and C of GP-ZW-HTNP@S, GP@S and G@S.

Supplemental Experimental Procedures.

The GP-ZW-HTNP@S shows slopes as low as 24.88, 32.9 and 48.5 mV dec^{-1}, compared to GP (46.57, 57.2 and 70.2 mV dec^{-1}) and G (72.83, 88.4 and 75.7 mV dec^{-1}), indicating more efficient sulfur species conversion on GP-ZW-HTNP surface. Peak A is the conversion of Li_2S to S_8, peak B is the conversion of Li_2S_4 to Li_2S or Li_2S_2, and peak C is the conversion of S_8 to Li_2S_4.

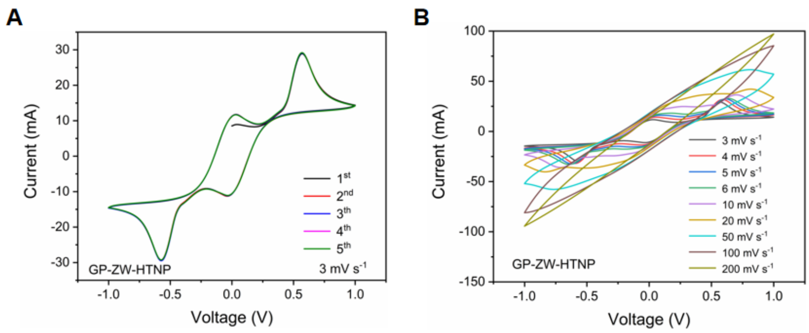

Figure S11. Symmetric CV curves.

(a) Symmetric CV curves of GP-ZW-HTNP at 3 mV s^{-1} different cycles.

(b) Symmetric CV curves of GP-ZW-HTNP at different scan rates.

Supplemental Experimental Procedures.

GP-ZW-HTNP cycles stably at 3 mV s^{-1} with almost identical curves, and even at the high rate of 200 mV s^{-1}, the curves still overlap and maintain consistent area.

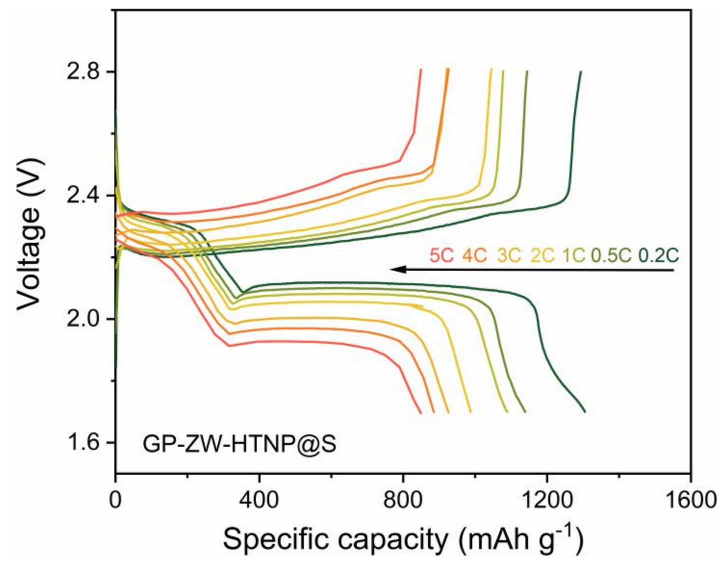

Figure S12. The charge-discharge curves at different current densities.

The charge-discharge curves of coin cells assembled by GP-ZW-HTNP@S at different current densities (room temperature).

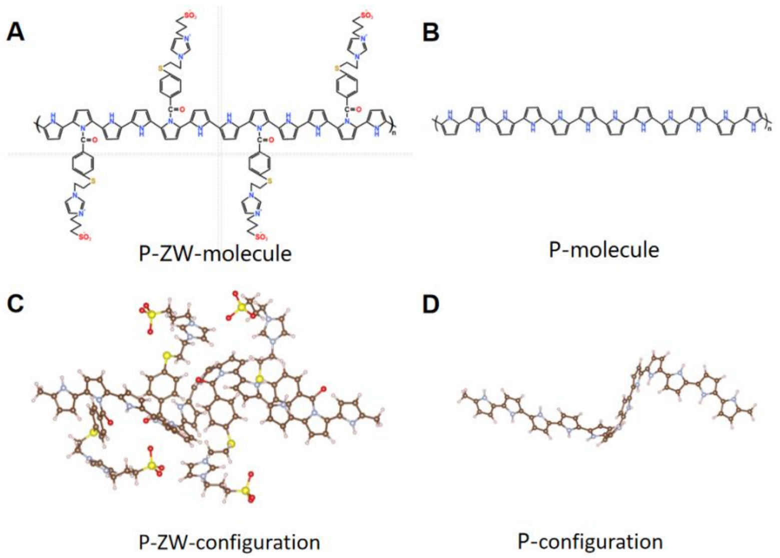

Figure S13. Molecular structure optimization model.

The (a) molecular structure and (c) configuration of P-ZW and the (b) molecular structure and (d) configuration of P. P-ZW is the structure of GP-ZW-HTNP after grafting an amphipathic ion on a conjugated chain segment. P is the conjugated chain segment alone.

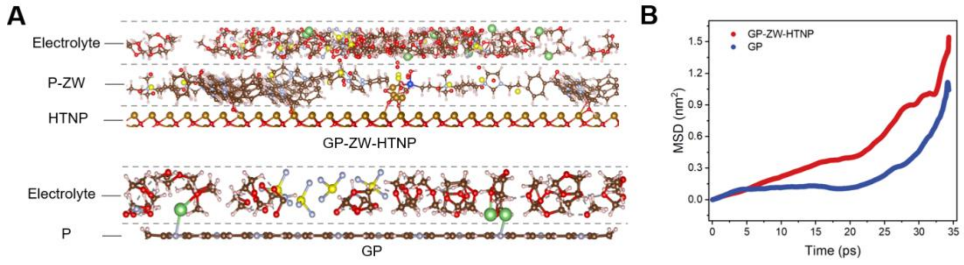

Figure 14. Simulation of interfacial Li⁺ diffusion processes for GP-ZW-HTNP and GP

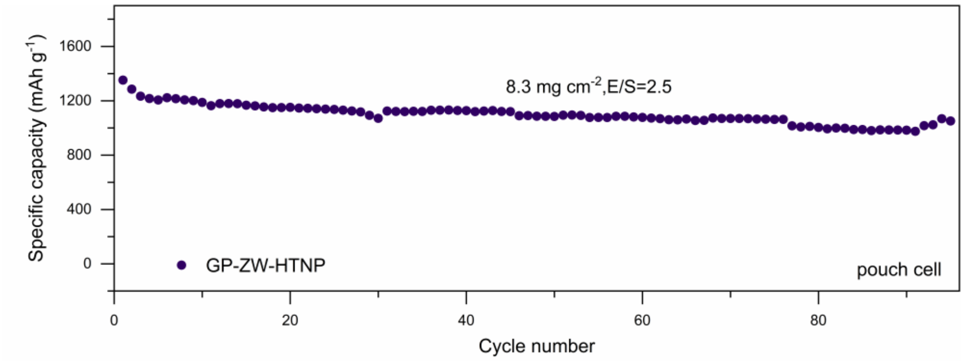

Figure S15. Cycle performance under sulfur loading and poor electrolyte conditions. Cycling performance of GP-ZW-HTNP@S at 0.2 C, 8.3 mg cm^{-2} sulfur loading and poor electrolyte with E/S=2. This is the performance of a pouch consisting of a single positive electrode and a single negative electrode.

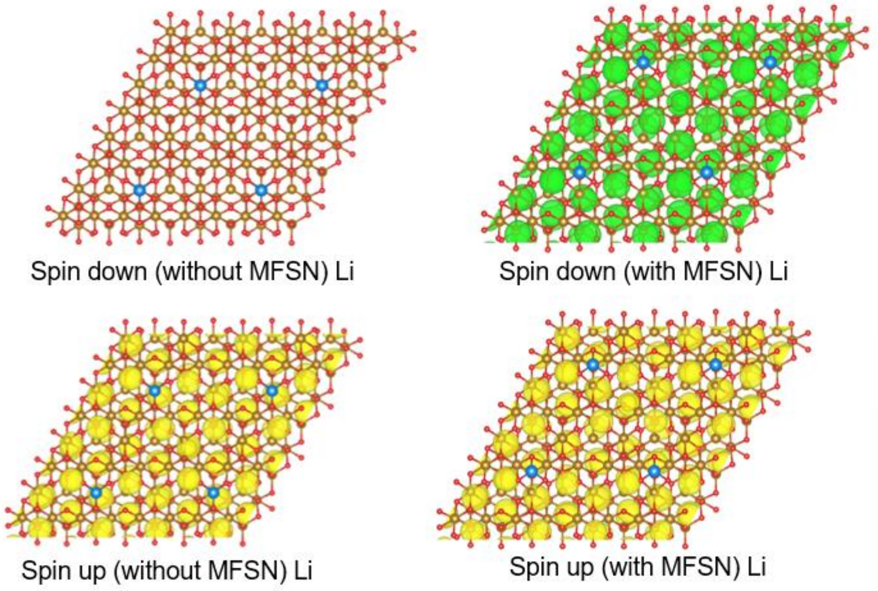

Figure S16. Spin density of Fe_3O_4 with/without a magnetic field (Li).

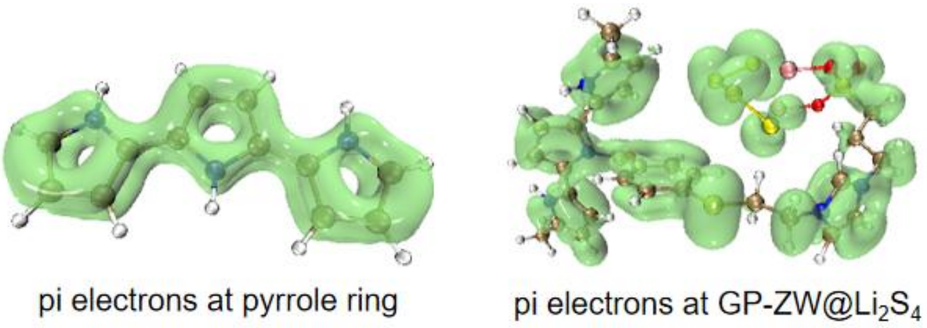

Figure S17. π -electron distribution of different molecular structure.

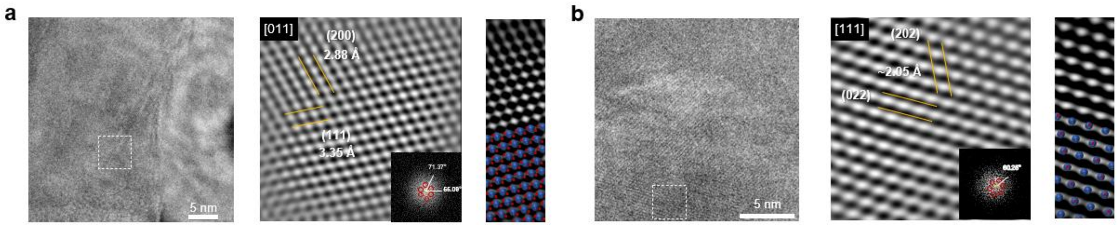

Figure S18. Radial Wiener-filtered HRTEM of Li_2S growth with MFSN at different zone axis..

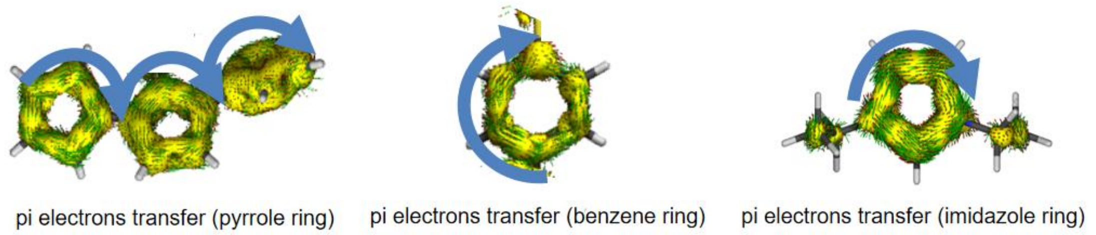

Figure S19. AICD calculation results of different molecular structure.

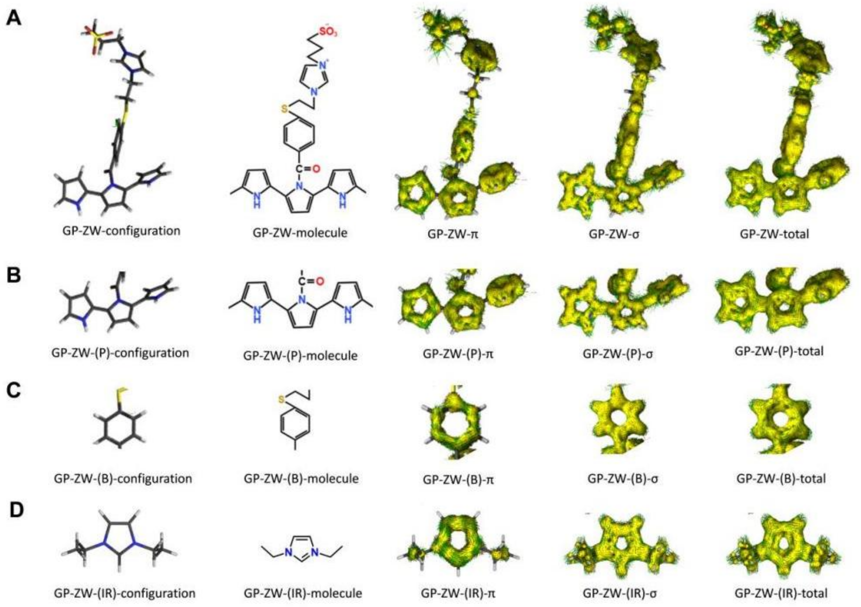

Figure S20. AICD 2.0 program under magnetic fields.

(a) GP-ZW, (b) GP-ZW-(P), (**c**) GP-ZW-(B) and (d) GP-ZW-(IR) configurations and molecular structures and the corresponding AICD 2.0 program under magnetic fields simulate the transfer of π-electrons, σ-electrons, and total-electrons in the molecular structure.

Supplemental Experimental Procedures.

GP-ZW is part of GP-ZW-HTNP, where only the effect of magnetic fields on the electrons in the structure of organic molecules is discussed. GP-ZW-(P), refers to the conjugated backbone (pyrrole) in GP-ZW alone. GP-ZW-(B) alone refers to the benzene ring in GP-ZW. GP-ZW-(IR) alone refers to the positively charged imidazole ring structure in GP-ZW. In the figure, it can be noticed that pie electrons all move away from their original position as a whole under a magnetic field, but sigma electrons do not. Magnetic fields can only enhance the off-domain nature of π-electrons, and the electron transfer formed is mainly provided by π-electrons.

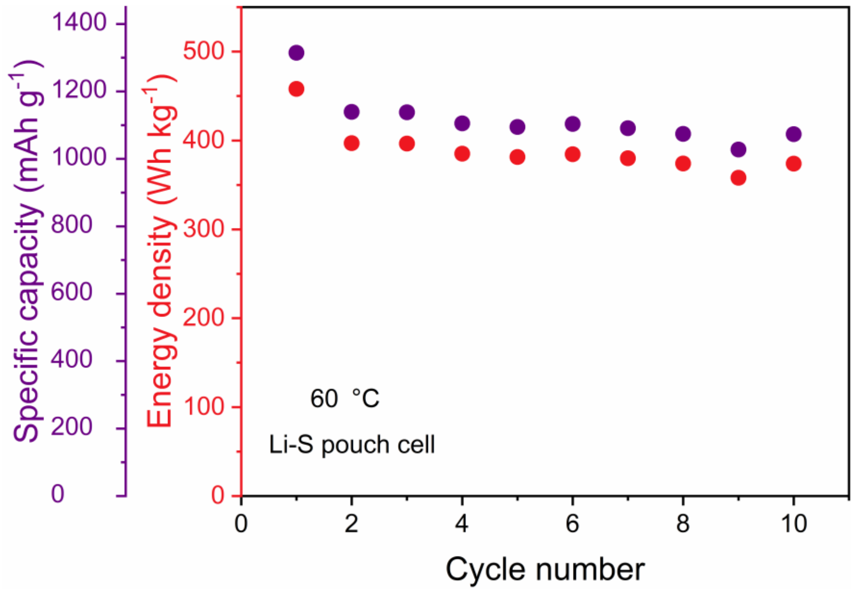

Figure S21. The cycle performance of the "smart symbiosis" pouch cell at 60 °C.

Cycling performance of ~2 Ah Li-S pouch cell assembled from GP-ZW-HTNP@S material at 0.05 C and 60 °C.

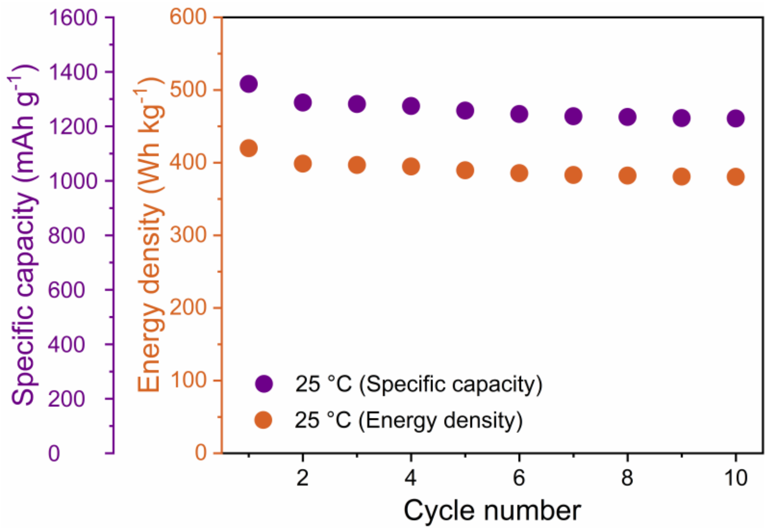

Figure S22. Discharge curve (capacity-voltage) of the pouch cell at 25 °C with E/S=2.5.

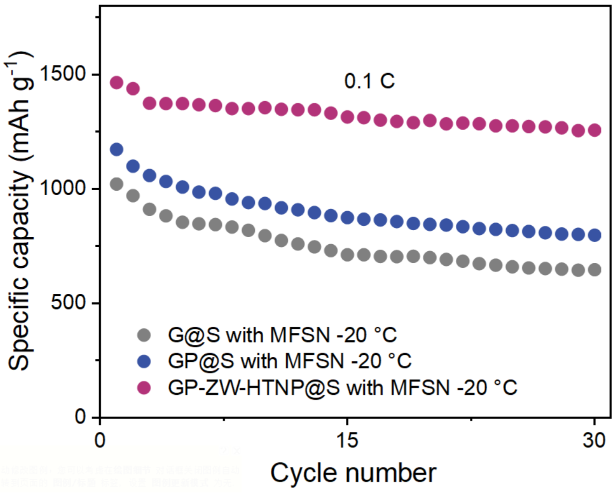

Figure S23. The cycle performance of G@S, GP@S, and GP-ZW-HTNP@S at -20 °C under the same magnetic field (large pouch cell).

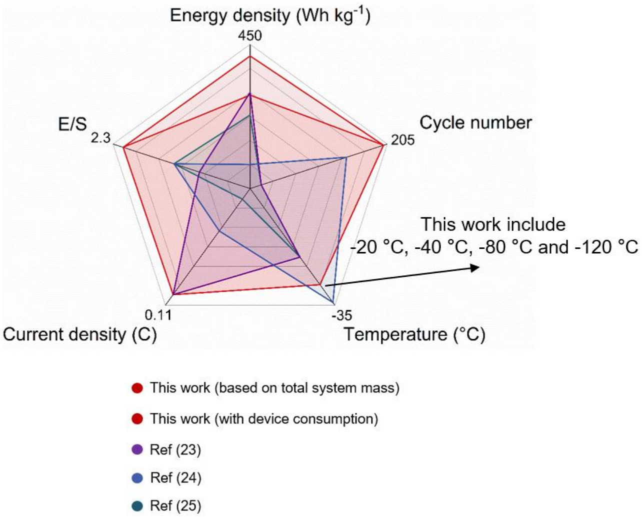

Figure S24. Electrochemical performance parameters of low-temperature pouch cells: this work vs. reported studies [23-25].

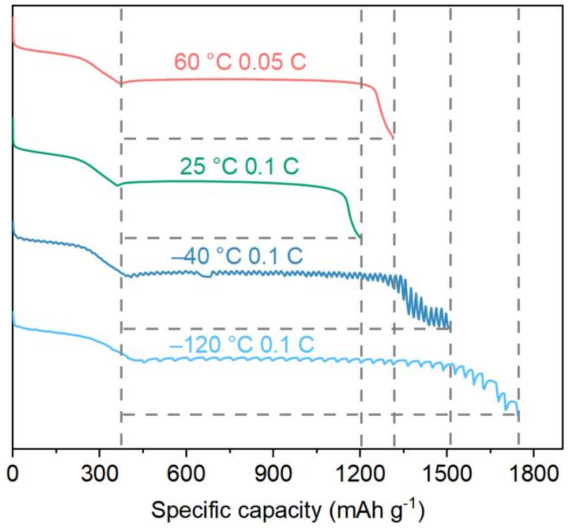

Figure S25. Discharge curves at 60 °C, 25 °C, –40 °C and –120 °C.

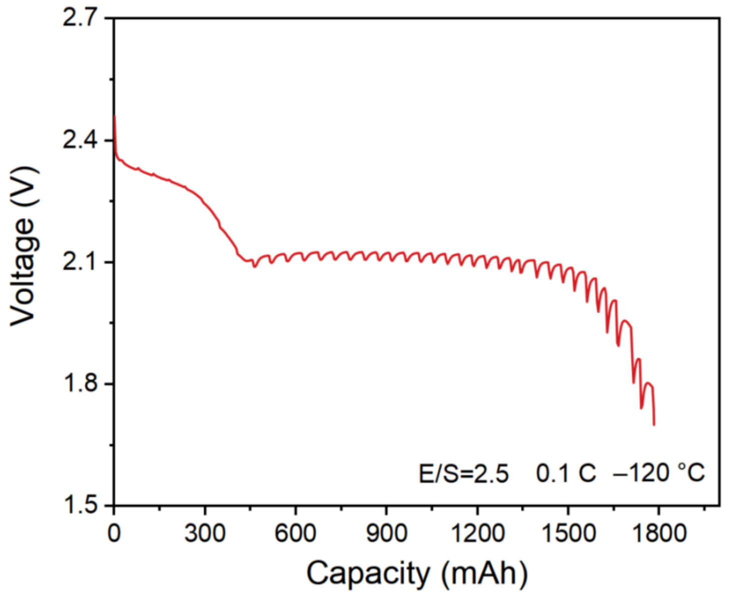

Figure S26. Discharge curve (capacity-voltage) of the pouch cell at –120 °C with E/S=2.5. Discharge curve (capacity-voltage) of the pouch cell at –120 °C, with a capacity close to ~1800 mAh.

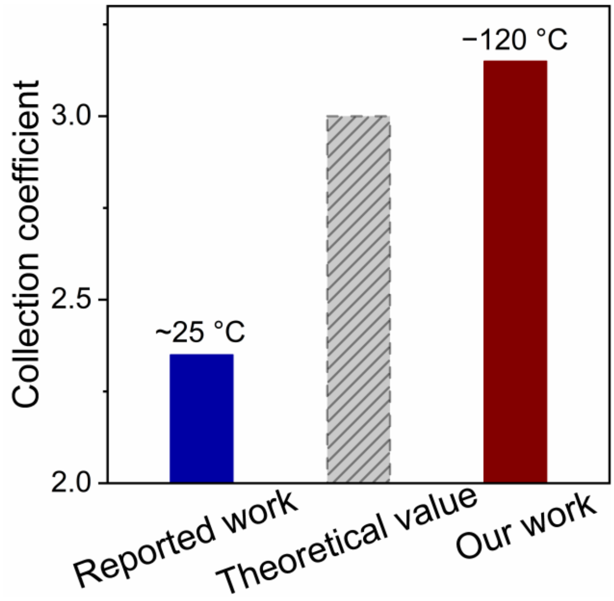

Figure S27. Theoretical collection coefficients.

Comparison between reported work, theoretical value and our work coefficients.

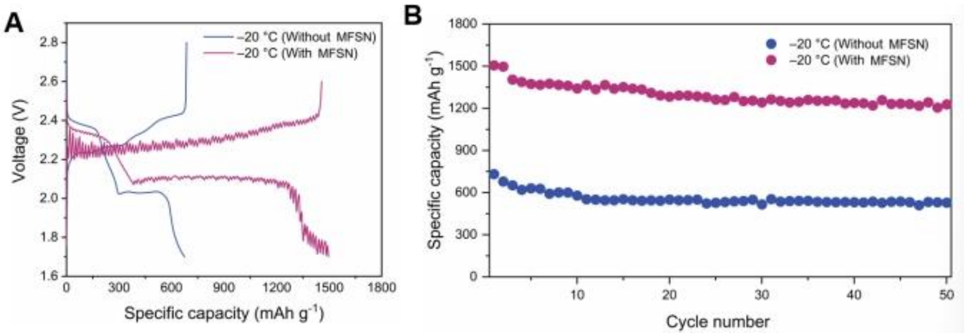

Figure S28. Comparison of battery performance with/without multi-field synergy at –20°C.

(A) Charge-discharge curves with/without multi-field synergy at –20°C.

(B) Cycling performance with/without multi-field synergy at –20°C.

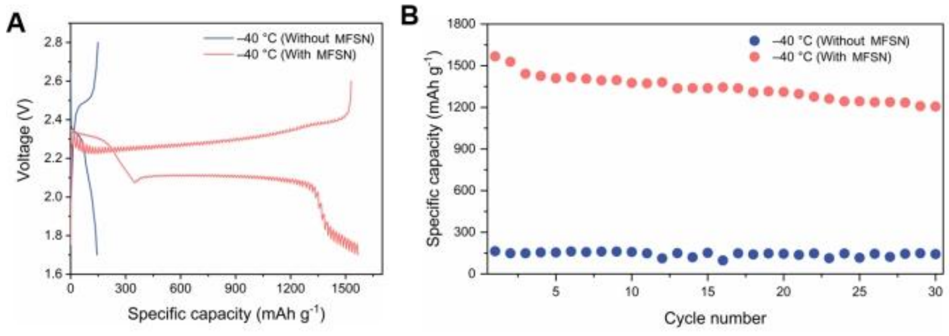

Figure S29. Comparison of battery performance with/without multi-field synergy at –40°C.

(A) Charge-discharge curves with/without multi-field synergy at –40°C.

(B) Cycling performance with/without multi-field synergy at –40°C.

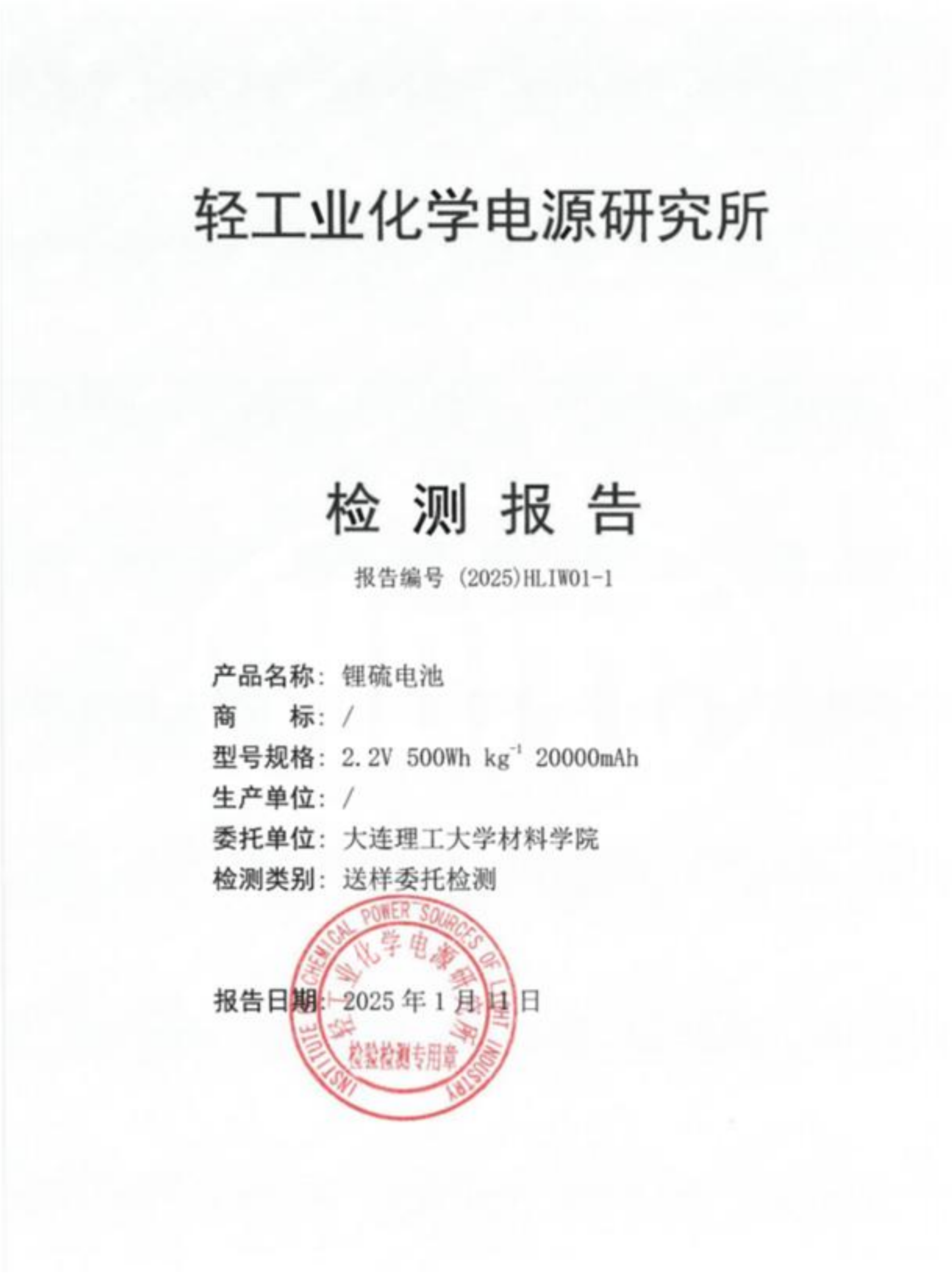

轻工业化学电源研究所

检 测 报 告

报告编号 (2025)HLIW01-1

产品名称：锂硫电池
商　　标：/
型号规格：2.2V 500Wh kg^{-1} 20000mAh
生产单位：/
委托单位：大连理工大学材料学院
检测类别：送样委托检测

报告日期：2025 年 1 月 11 日

Figure S30. Third-party test report of pouch cell (page 1).

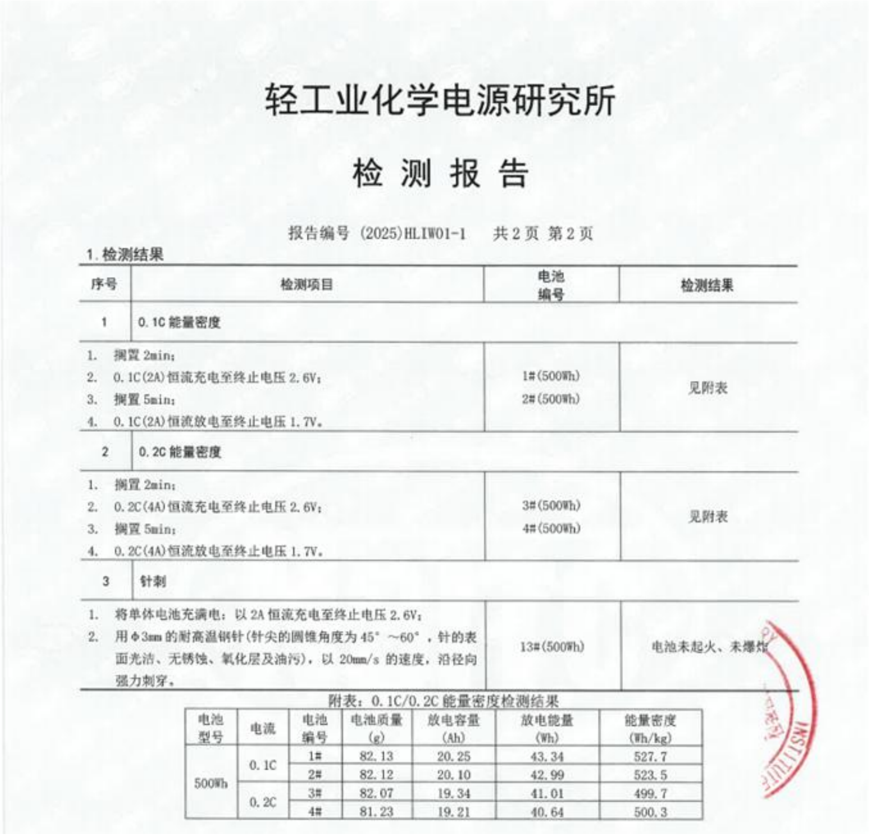

轻工业化学电源研究所

检 测 报 告

报告编号 (2025)HLIW01-1　共2页 第2页

1.检测结果

序号	检测项目	电池编号	检测结果
1	0.1C 能量密度		
1. 搁置 2min;<br>2. 0.1C(2A)恒流充电至终止电压 2.6V;<br>3. 搁置 5min;<br>4. 0.1C(2A)恒流放电至终止电压 1.7V。		1#(500Wh)<br>2#(500Wh)	见附表
2	0.2C 能量密度		
1. 搁置 2min;<br>2. 0.2C(4A)恒流充电至终止电压 2.6V;<br>3. 搁置 5min;<br>4. 0.2C(4A)恒流放电至终止电压 1.7V。		3#(500Wh)<br>4#(500Wh)	见附表
3	针刺		
1. 将单体电池充满电：以 2A 恒流充电至终止电压 2.6V;<br>2. 用Φ3mm 的耐高温钢针(针尖的圆锥角度为 45°～60°，针的表面光洁、无锈蚀、氧化层及油污)，以 20mm/s 的速度，沿径向强力刺穿。		13#(500Wh)	电池未起火、未爆炸

附表：0.1C/0.2C 能量密度检测结果

电池型号	电流	电池编号	电池质量 (g)	放电容量 (Ah)	放电能量 (Wh)	能量密度 (Wh/kg)
500Wh	0.1C	1#	82.13	20.25	43.34	527.7
		2#	82.12	20.10	42.99	523.5
	0.2C	3#	82.07	19.34	41.01	499.7
		4#	81.23	19.21	40.64	500.3

Figure S31. Third-party test report of pouch cell (page 2).

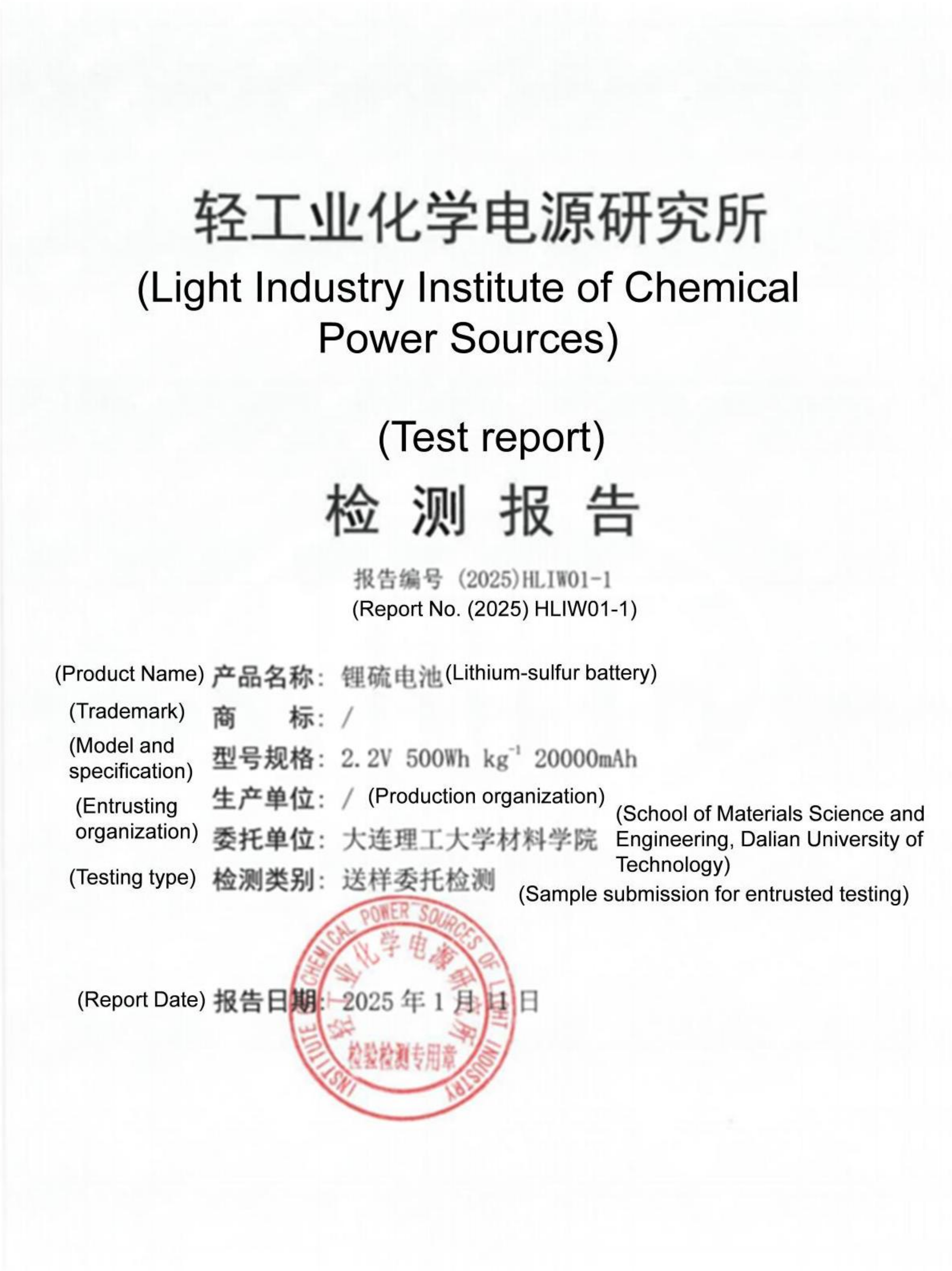

轻工业化学电源研究所

(Light Industry Institute of Chemical Power Sources)

(Test report)

检 测 报 告

报告编号 (2025)HLIW01-1

(Report No. (2025) HLIW01-1)

(Product Name) 产品名称：锂硫电池 (Lithium-sulfur battery)

(Trademark) 商　　标：/

(Model and specification) 型号规格：2.2V 500Wh kg^{-1} 20000mAh

生产单位：/ (Production organization)

(Entrusting organization) 委托单位：大连理工大学材料学院 (School of Materials Science and Engineering, Dalian University of Technology)

(Testing type) 检测类别：送样委托检测 (Sample submission for entrusted testing)

(Report Date) 报告日期：2025 年 1 月 11 日

Figure S32. Third-party test report of pouch cell (page 1)-English version.

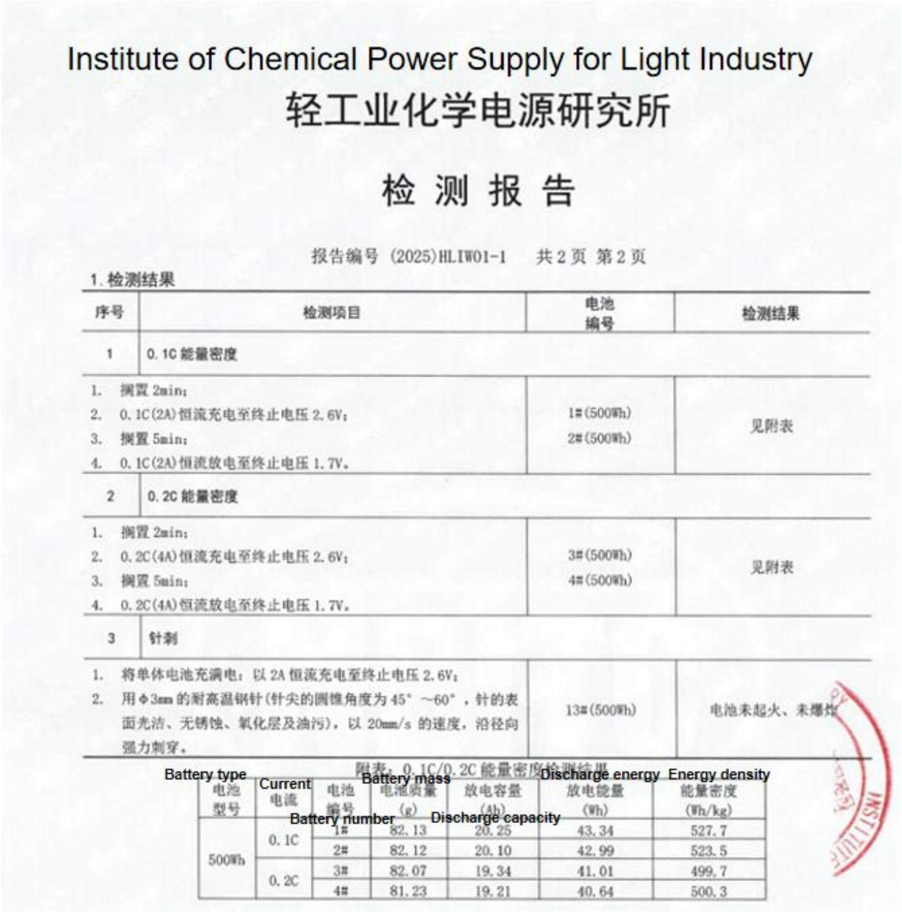

Institute of Chemical Power Supply for Light Industry

轻工业化学电源研究所

检 测 报 告

报告编号 (2025)HLIW01-1　共 2 页 第 2 页

1. 检测结果

序号	检测项目	电池编号	检测结果
1	0.1C 能量密度		
	1. 搁置 2min;<br>2. 0.1C(2A)恒流充电至终止电压 2.6V;<br>3. 搁置 5min;<br>4. 0.1C(2A)恒流放电至终止电压 1.7V。	1#(500Wh)<br>2#(500Wh)	见附表
2	0.2C 能量密度		
	1. 搁置 2min;<br>2. 0.2C(4A)恒流充电至终止电压 2.6V;<br>3. 搁置 5min;<br>4. 0.2C(4A)恒流放电至终止电压 1.7V。	3#(500Wh)<br>4#(500Wh)	见附表
3	针刺		
	1. 将单体电池充满电：以 2A 恒流充电至终止电压 2.6V;<br>2. 用Φ3mm 的耐高温钢针(针尖的圆锥角度为 45°～60°，针的表面光洁、无锈蚀、氧化层及油污)，以 20mm/s 的速度，沿径向强力刺穿。	13#(500Wh)	电池未起火、未爆炸

附表：0.1C/0.2C 能量密度检测结果

电池型号 (Battery type)	电流 (Current)	电池编号 (Battery number)	电池质量 (g) (Battery mass)	放电容量 (Ah) (Discharge capacity)	放电能量 (Wh) (Discharge energy)	能量密度 (Wh/kg) (Energy density)
500Wh	0.1C	1#	82.13	20.25	43.34	527.7
		2#	82.12	20.10	42.99	523.5
	0.2C	3#	82.07	19.34	41.01	499.7
		4#	81.23	19.21	40.64	500.3

Figure S33. Third-party test report of pouch cell (page 2)-English version.

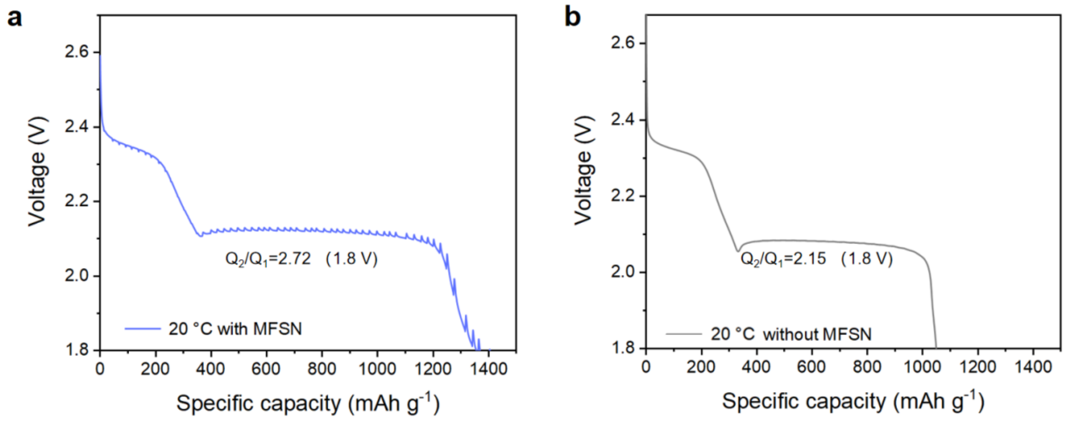

Figure S34. Comparison of discharge curves for batteries with and without MFSN at room temperature.

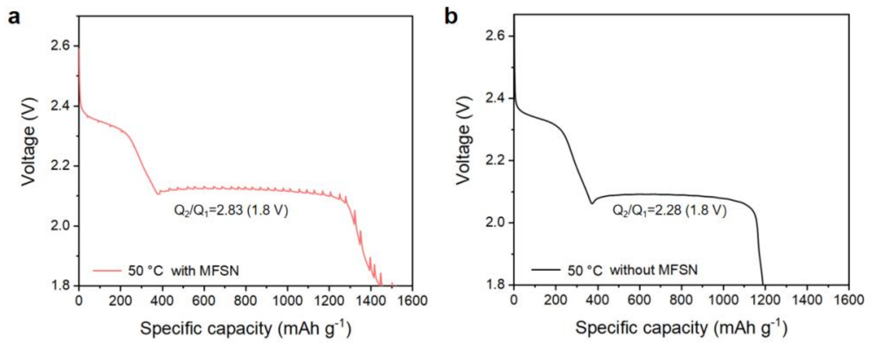

Figure S35. Comparison of discharge curves for batteries with and without MFSN at high temperature.

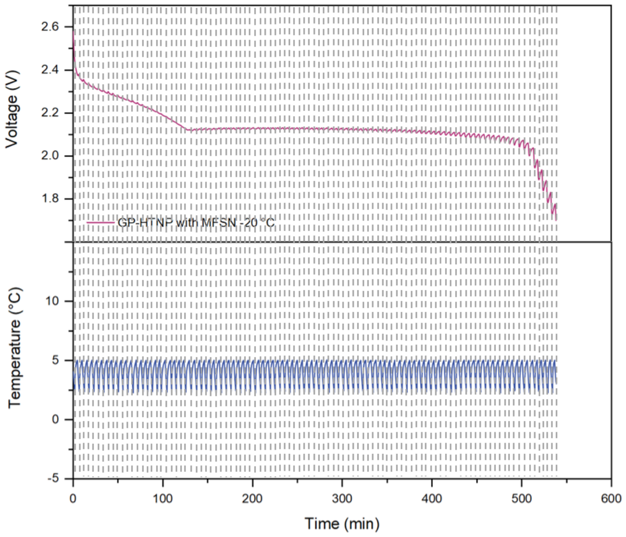

Figure S36. The time-voltage curve and time-temperature curve with MFSN at -20 °C.

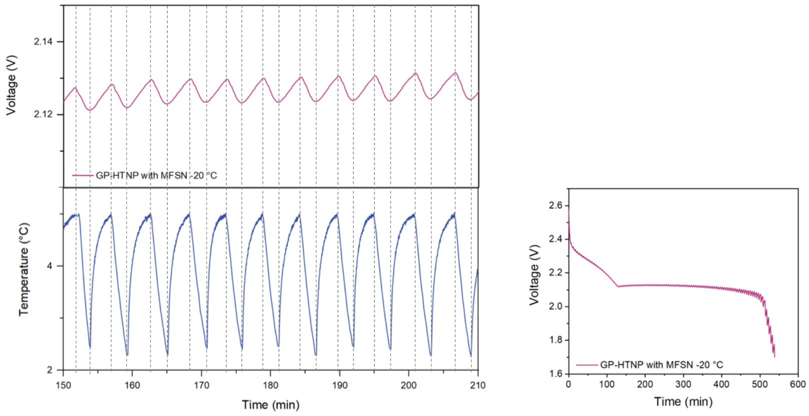

Figure S37. The time-voltage curve and time-temperature curve (partial view) with MFSN at -20 °C.

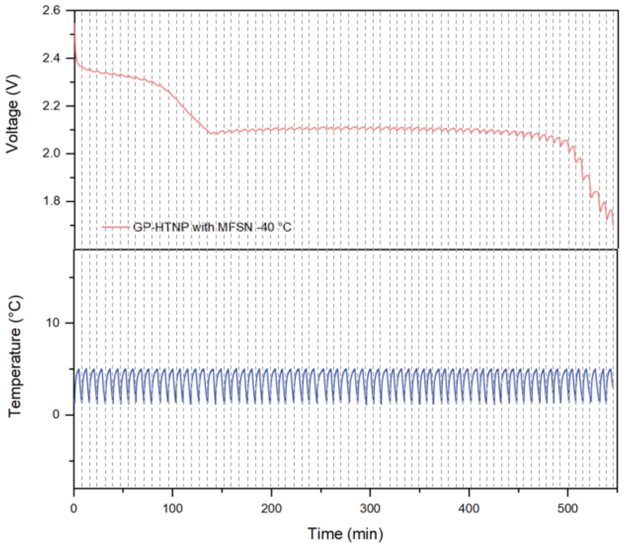

Figure S38. The time-voltage curve and time-temperature curve with MFSN at -40 °C.

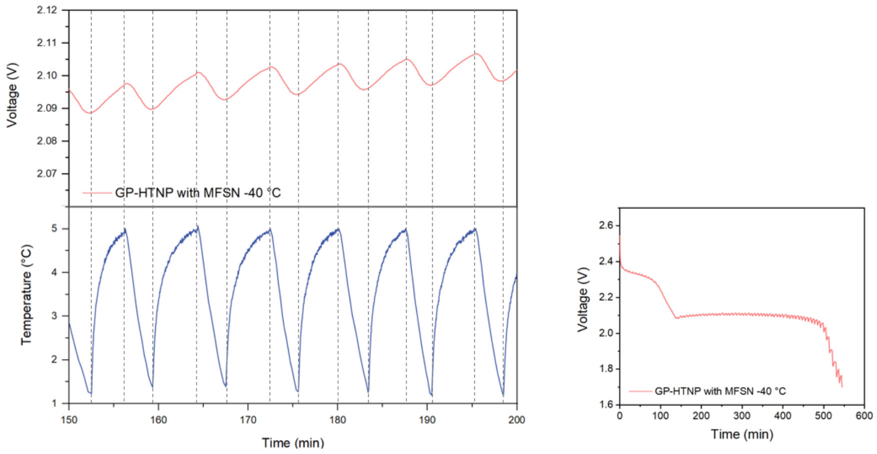

Figure S39. The time-voltage curve and time-temperature curve (partial view) with MFSN at -40 °C.

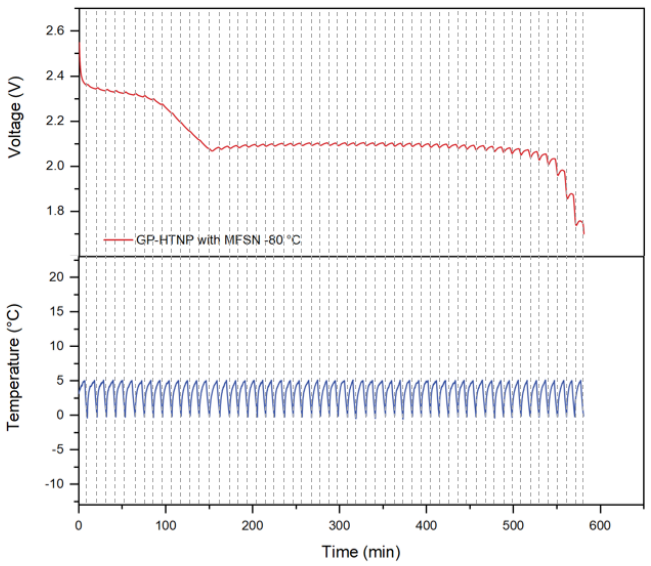

Figure S40. The time-voltage curve and time-temperature curve with MFSN at -80 °C.

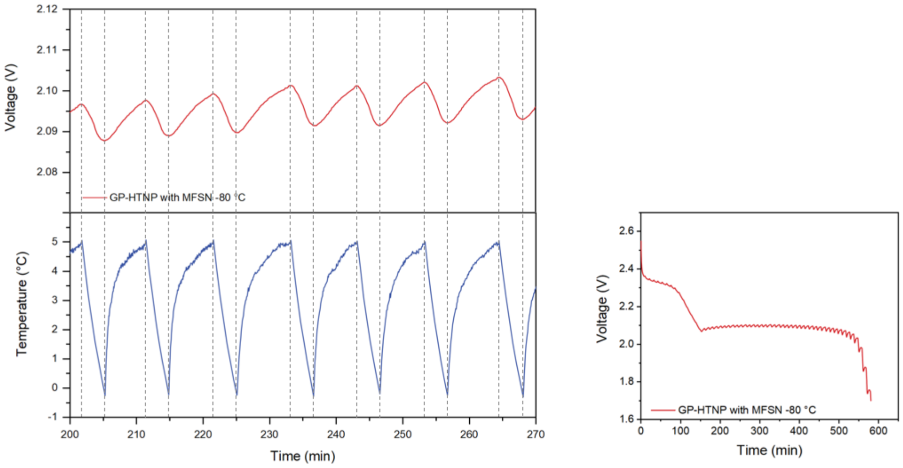

Figure S41. The time-voltage curve and time-temperature curve (partial view) with MFSN at -80 °C.

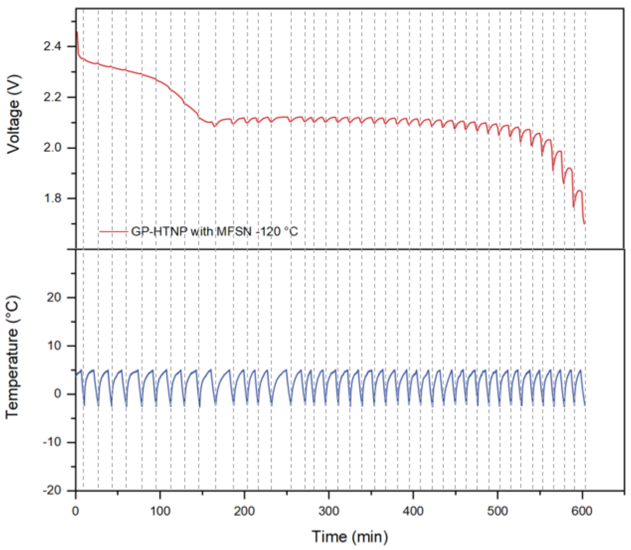

Figure S42. The time-voltage curve and time-temperature curve with MFSN at -120 °C.

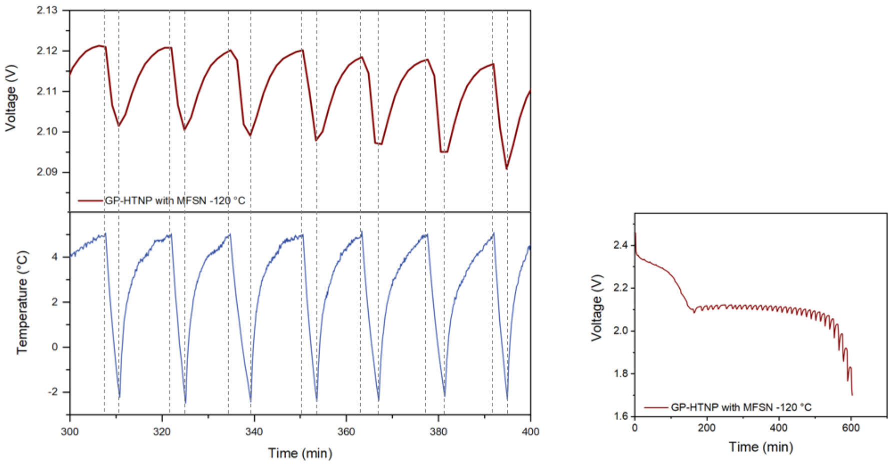

Figure S43. The time-voltage curve and time-temperature curve (partial view) with MFSN at -120 °C.

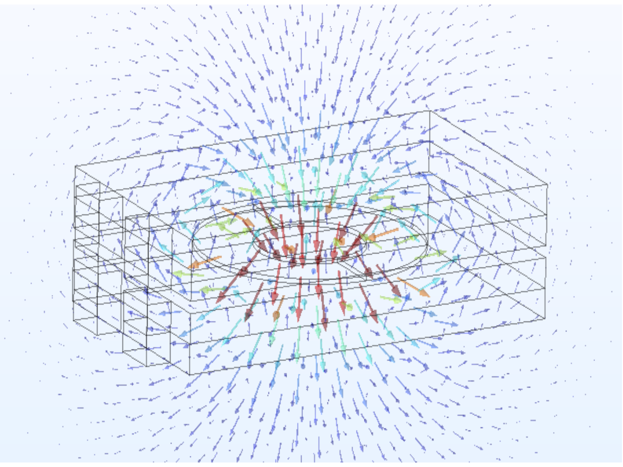

Figure S44. The spatial distribution of the magnetic field at -20 °C.

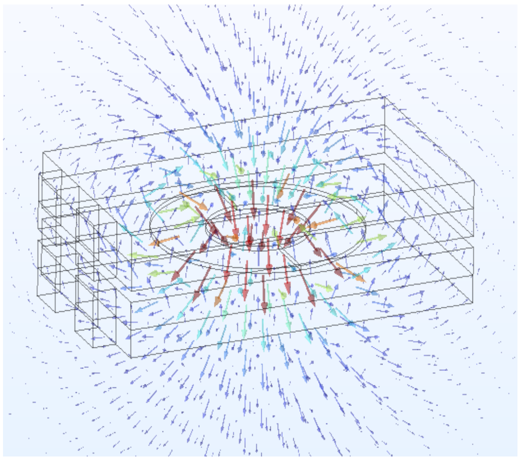

Figure S45. The spatial distribution of the magnetic field at -40 °C.

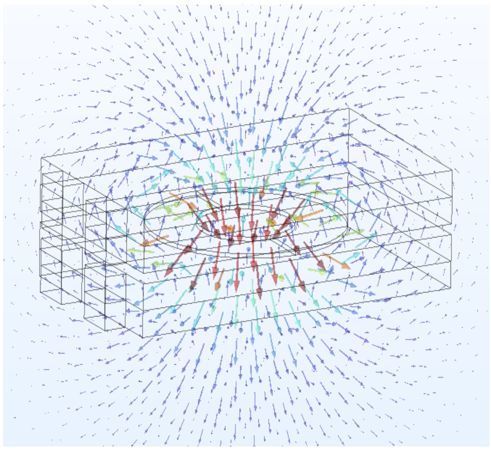

Figure S46. The spatial distribution of the magnetic field at -80 °C.

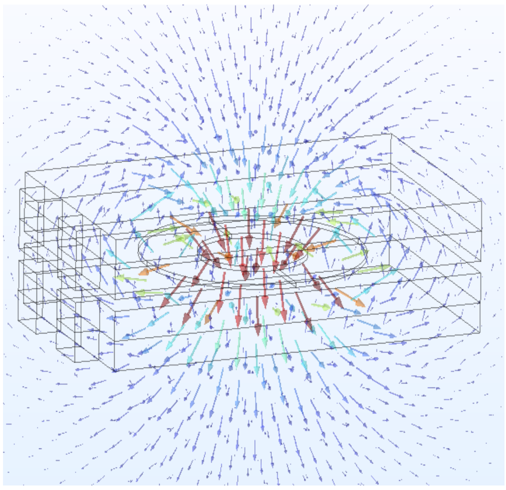

Figure S47. The spatial distribution of the magnetic field at -120 °C.

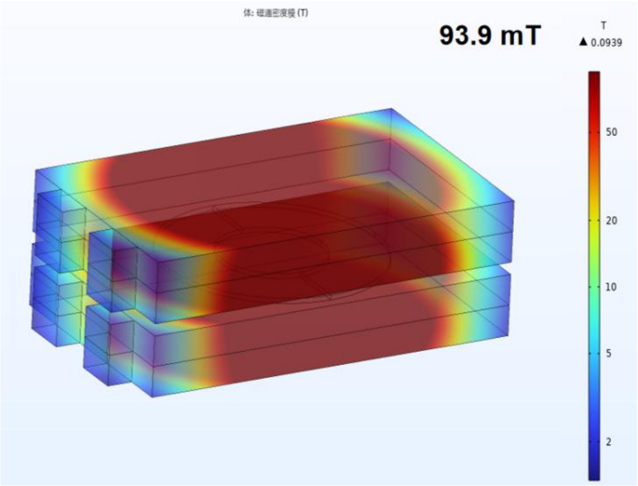

Figure S48. Magnetic field intensity at -20 °C.

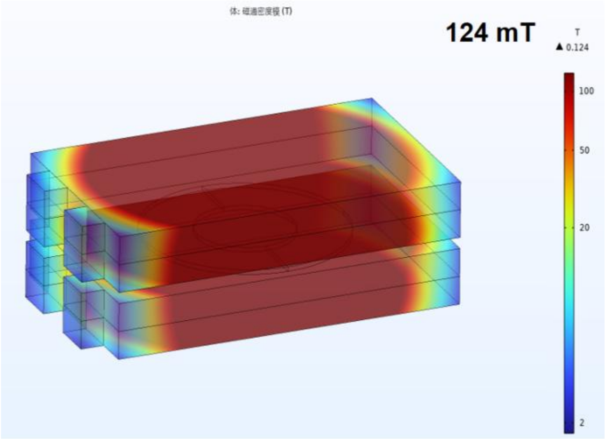

Figure S49. Magnetic field intensity at -40 °C.

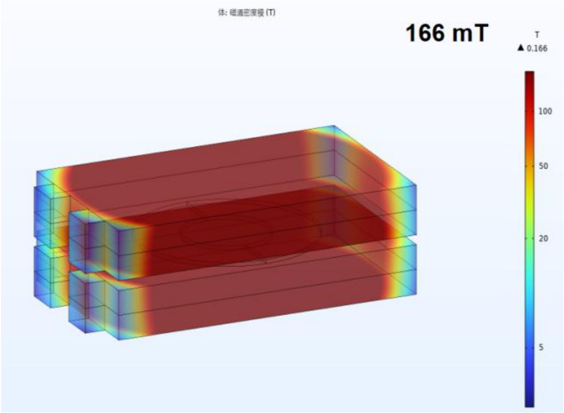

Figure S50. Magnetic field intensity at -80 °C.

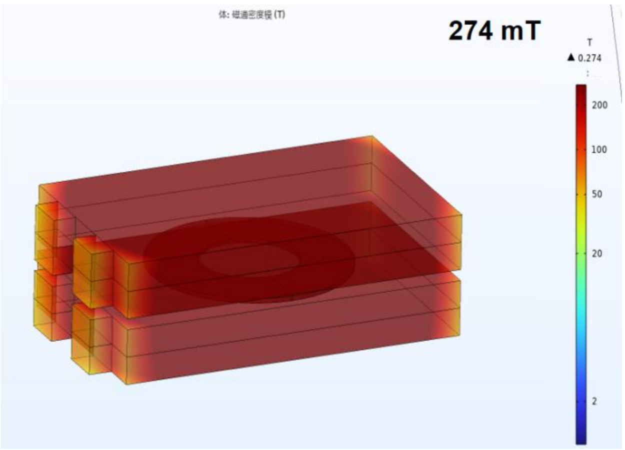

Figure S51. Magnetic field intensity at -120 °C.

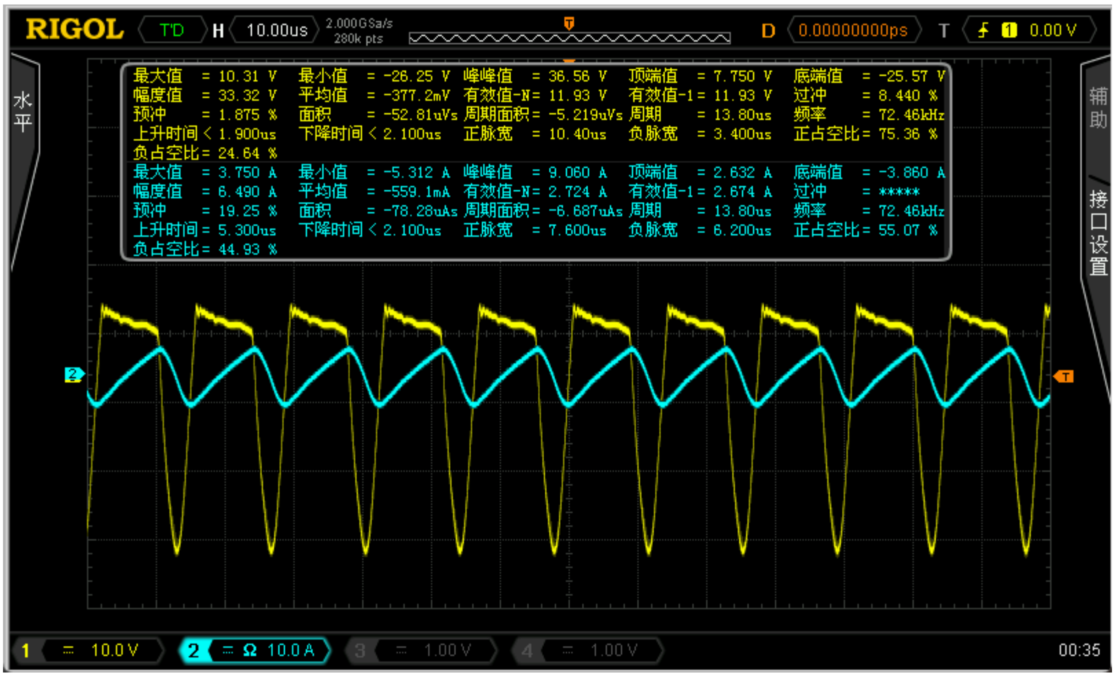

Figure S52. Current waveform, voltage waveform, frequency, and other relevant parameters of the magnetic field device at -20 °C.

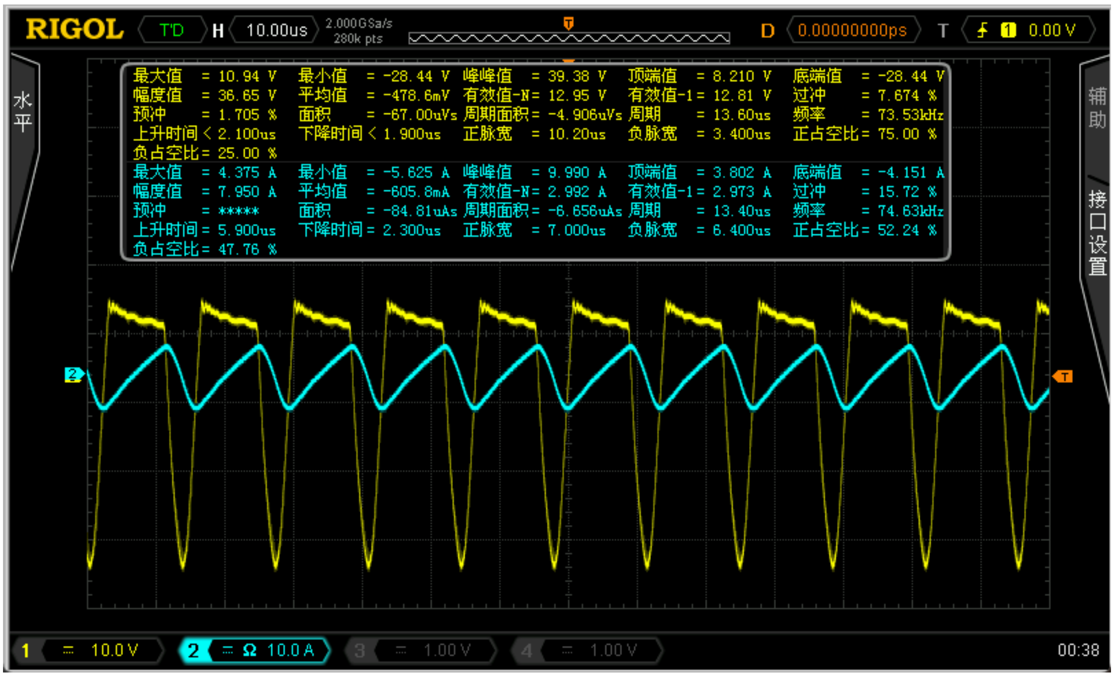

Figure S53. Current waveform, voltage waveform, frequency, and other relevant parameters of the magnetic field device at -40 °C.

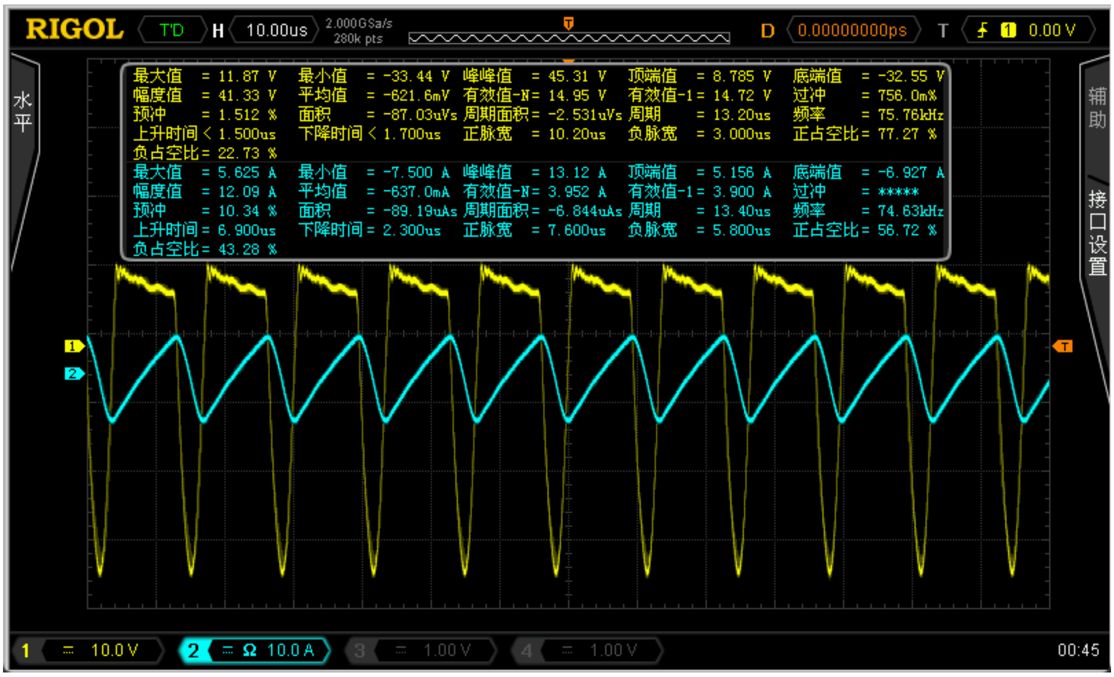

Figure S54. Current waveform, voltage waveform, frequency, and other relevant parameters of the magnetic field device at -80 °C.

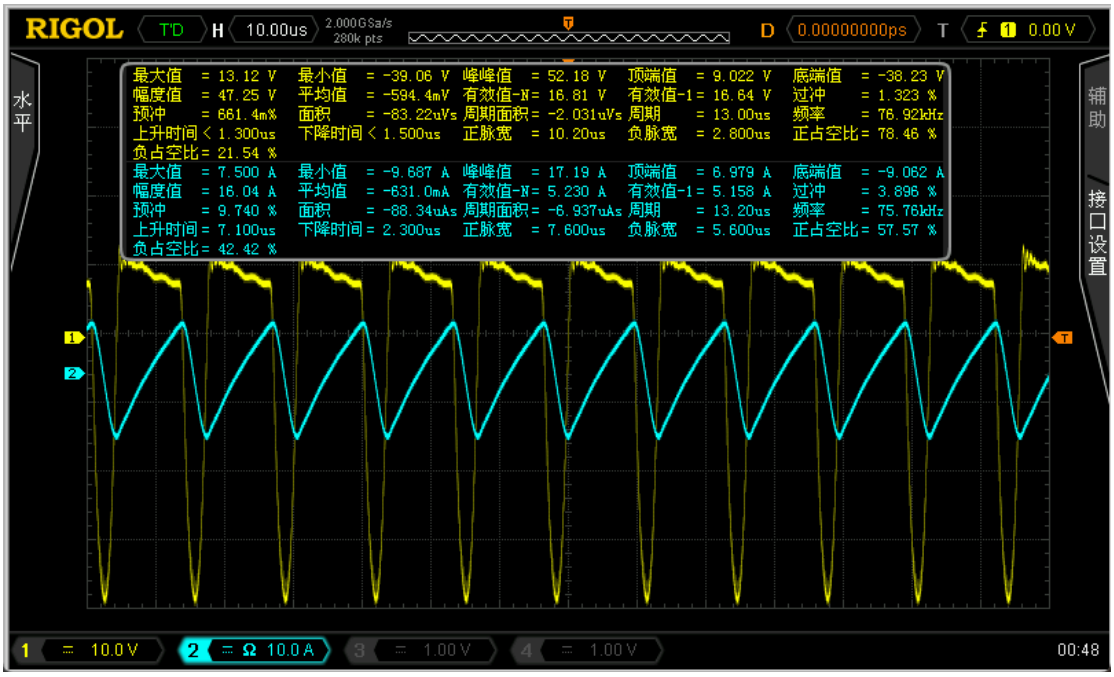

Figure S55. Current waveform, voltage waveform, frequency, and other relevant parameters of the magnetic field device at -120 °C.

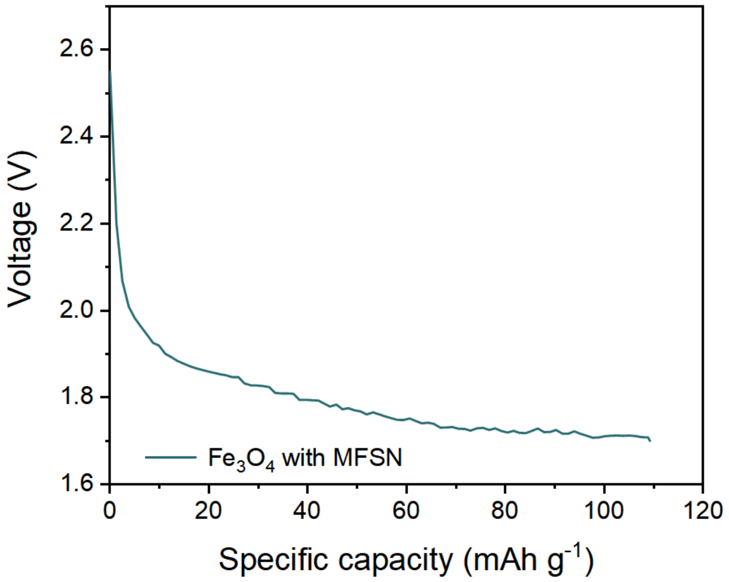

Figure S56. The discharge curve of the pure Fe_3O_4 cathode with MFSN within the voltage range of 2.8 V to 1.7 V.

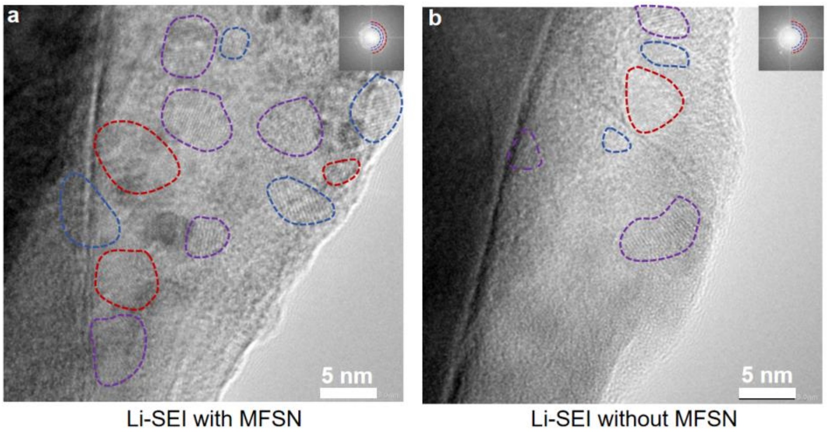

Figure S57. SEI composition of the lithium metal anode with and without MFSN.

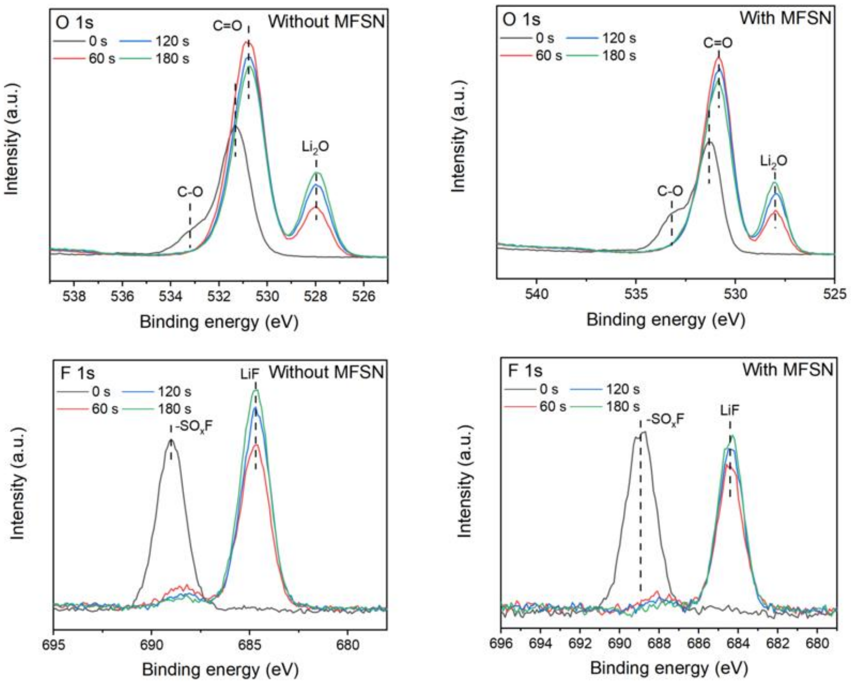

Figure S58. XPS (X-ray photoelectron spectroscopy) depth etching of SEI.

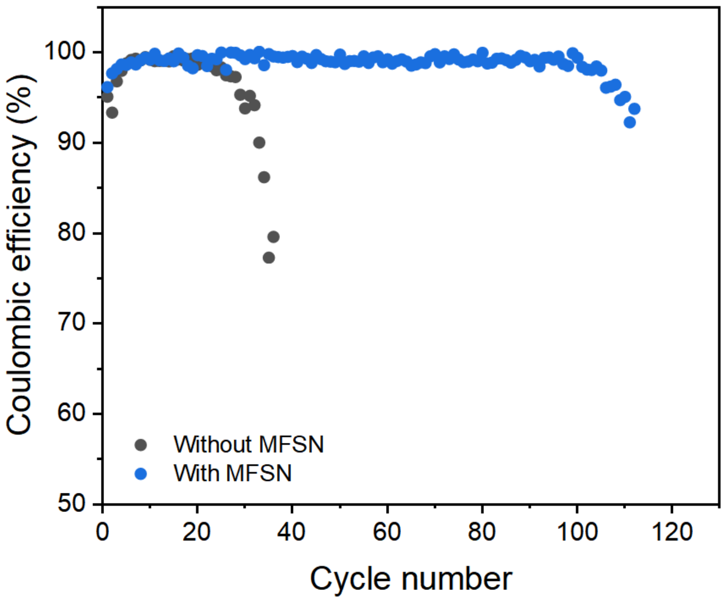

Figure S59. Li-Cu tests of the lithium anode after cycling with and without a magnetic field.

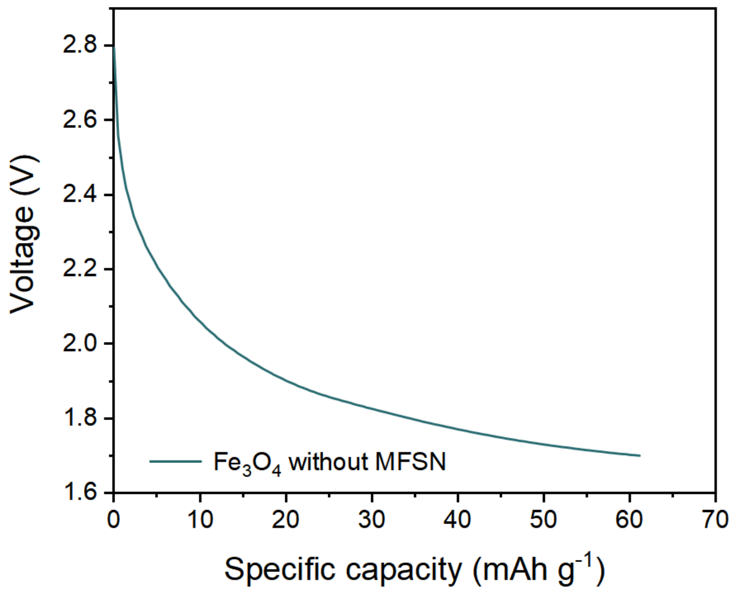

Figure S60. The discharge curve of the pure Fe_3O_4 cathode without MFSN within the voltage range of 2.8 V to 1.7 V.

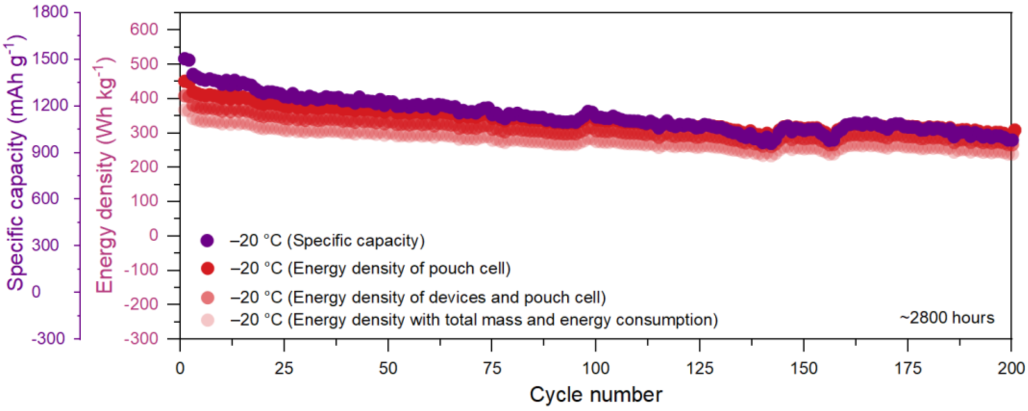

Figure S61. Cycling performance of pouch cells at –20 °C (~2800 hours).

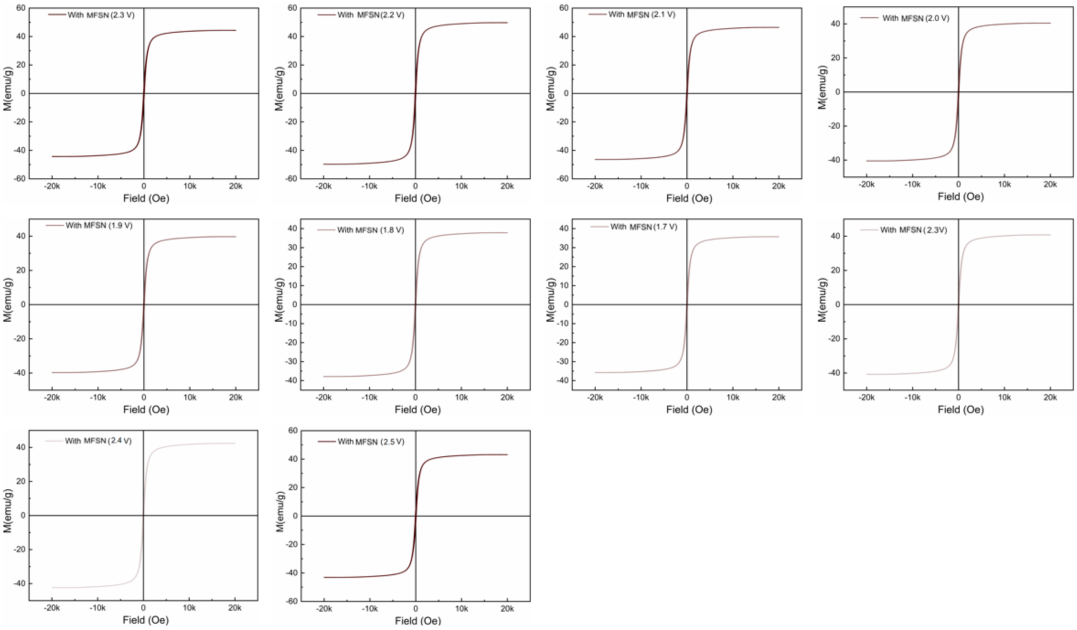

Figure S62. Magnetic hysteresis loops of cathode materials in pouch cell with MFSN (after being discharged at 0.1 C to 2.3 V, 2.2 V, 2.1 V, 2.0 V, 1.9 V, 1.8 V, 1.7 V and then charged to 2.3 V, 2.4 V, and 2.5 V respectively).

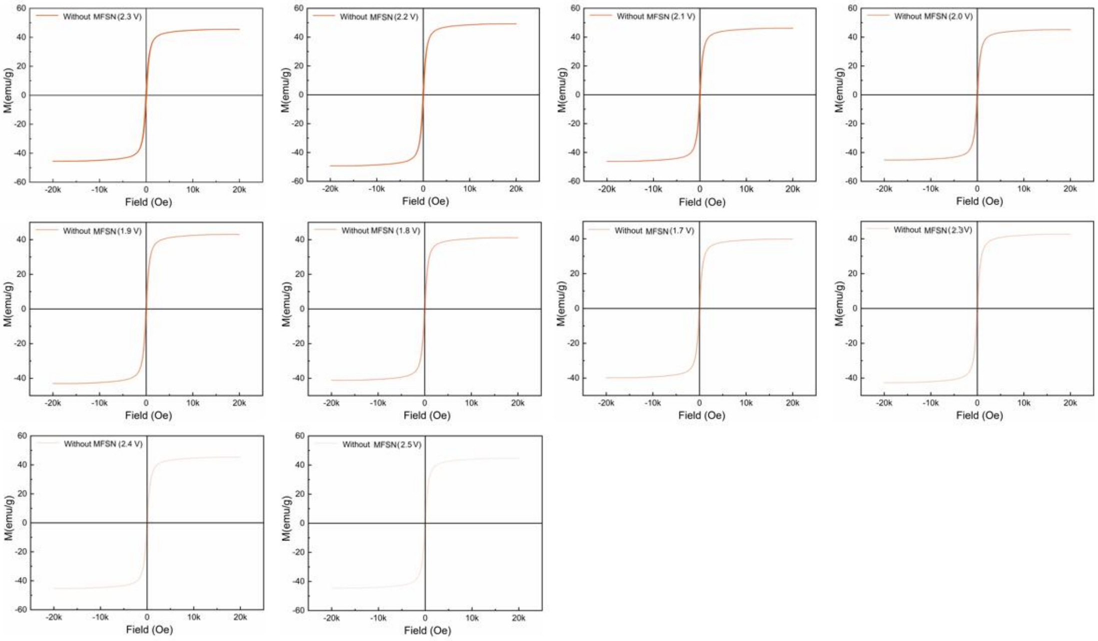

Figure S63. Magnetic hysteresis loops of cathode materials in pouch cell without MFSN (after being discharged at 0.1 C to 2.3 V, 2.2 V, 2.1 V, 2.0 V, 1.9 V, 1.8 V, 1.7 V and then charged to 2.3 V, 2.4 V, and 2.5 V respectively).

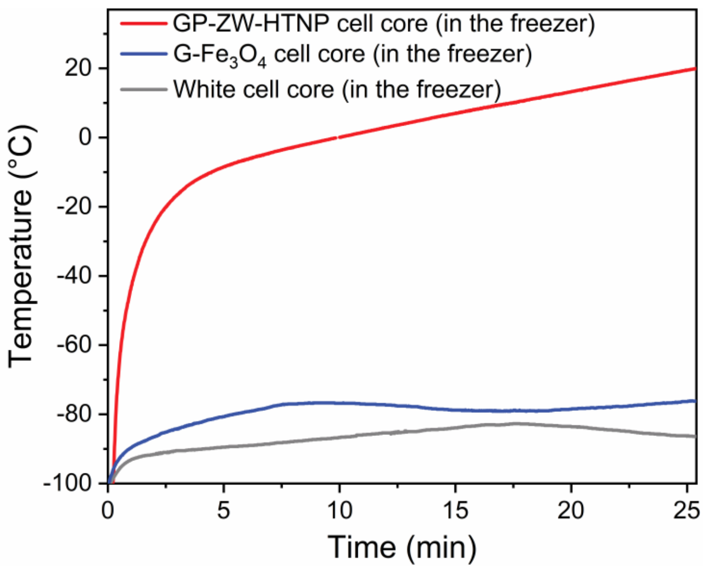

Figure S64. The internal temperature rise curves.

The internal temperature rise curves of the cells containing GP-ZW-HTNP, G-Fe_3O_4 and Al under the magnetic field.

Supplemental Experimental Procedures.

The whole process was carried out in a refrigerator set at −120 °C, which worked continuously to cool down the temperature. The aluminium foil containing GP-ZW-HTNP and G - Fe_3O_4 was coated on both sides. The resulting pole pieces are stacked one layer at a time as a simulated core. White group is aluminium foil without paste coating. The internal temperature variation of the core is recorded in real time by T-0.05. It took about 25 minutes for the GP-ZW-HTNP cell to come up to room temperature, while G-Fe_3O_4 and White were barely rose. This is mainly due to the better magnetic thermal efficiency of GP - ZW - HTNP. Also, the weak heat generated by G-Fe_3O_4 and White escaped during the continuous temperature control of the refrigerator. Notably, in “smart symbiosis” cells, the temperature is not allowed to drop to −120 °C before the magnetic field is started. The internal temperature of the pouch cell is adjusted in real time based on feedback.

Section 4. Supplementary Table

Table S1: Comparison of electrochemical performance of present work with various materials.

Reported high performance battery	Temperature[a] (°C)	Energy density[b] ($Wh\ kg^{-1}$)	Current[c]	Type[e]
$NiSe_2$ Catalyst[11]	25	402	20 mA	LSB
Lithiated MoS_2 nanosheets[22]	25	441	2 mA cm^{-2}	LSB
0.5M LiTFSI electrolyte[10]	0	350	0.1 C	LSB
AMDS modified electrolyte[7]	–60	Coin	0.05 C	LSB
Our work	–120	514.7	0.1 C	LSB
Anode to cathode of 1:1[14]	25	350	0.1 C	LMB
Self-smoothing Li-C anode[13]	25	350-380	0.2 C	LMB
Borate-pyran lean electrolyte[12]	25	~400	0.5 C	LMB
1M LiTFSI DEE electrolyte[16]	–60	Coin (76%)	0.2 C	LMB
LiFSI-NaPFO/THF electrolyte[15]	–85	122	--	LMB
Porous current collector[18]	25	276	--	LiB
$Mg_{16}Bi_{84}$ interlayer[17]	25	310	2.55 mA cm^{-2}	LiB
MDFA/MDFSA-TTE electrolyte[8]	–60	Coin	0.2 C	LiB
FAN based electrolyte[9]	–80	-- (37.5%)	6 C	LiB
AFNB anode-free[19]	25	~200	0.5 A	SiB
Dual-salt electrolyte[20]	–40	110	0.05 C	SiB
Anti-freezing electrolyte[21]	–85	12.5	0.05 C	SiB

[a] External temperature of the battery during cycle.

[b] The first cycle energy density of the battery.

[c] Current or Current density.

[d] The type of the battery.

Table S2: **The pouch cell detailed parameters at –20 °C.**

Parameter	Value
Cathode (current collector+materials)	1.035 g (220 mg+815.08 mg)
Anode (current collector+materials)	0.991 g (492 mg+498.5 mg)
Tab	0.01453 g<br>0.4% (follow 20 Ah pouch cell)
Packaging materials	0.07337 g<br>2.02% (follow 20 Ah pouch cell)
Electrolyte (E/S=2.5)	1.453 g
Separator	0.153 g
Chip	0.0563 g<br>1.55% (follow module)
Magnetic field emitter	0.0643 g<br>1.77% (follow module)
Microsensor	0.0058 g<br>0.16% (follow module)
Temperature	–20 °C
Total mass	3.846 g

Discharge capacity	779.75 mAh
Energy consumption	149.06 mAh<br>0.0395% of battery energy
Current density	0.1 C
Specific capacity	1505.31 mAh g^{-1}
Energy density	443.6 Wh kg^{-1}
Energy density (with module weight)	425.8 Wh kg^{-1}
Energy density (with energy consumption and module weight)	344.4 Wh kg^{-1}
Capacity comparison with/without MFSN	1505.31：752.44=200%

Table S3: The pouch cell detailed parameters at –40 °C.

Parameter	Value
Cathode (current collector+materials)	1.023 g (220 mg+803.14 mg)
Anode (current collector+materials)	0.9893 g (492 mg+497.3 mg)
Tab	0.01435 g<br>0.4% (follow 20 Ah pouch cell)
Packaging materials	0.0725 g<br>2.02% (follow 20 Ah pouch cell)
Electrolyte (E/S=2.5)	1.425 g
Separator	0.151 g
Chip	0.0556 g<br>1.55% (follow module)
Magnetic field emitter	0.0635 g<br>1.77% (follow module)
Microsensor	0.00573 g<br>0.16% (follow module)
Temperature	–40 °C
Total mass	3.799 g

Discharge capacity	802.65 mAh
Energy consumption	231.73 mAh<br>0.0922% of battery energy (module)
Current density	0.1 C
Specific capacity	1567.69 mAh g^{-1}
Energy density	458.6 Wh kg^{-1}
Energy density (with module weight)	443.7 Wh kg^{-1}
Energy density (with energy consumption and module weight)	315.6 Wh kg^{-1}
Capacity comparison with/without MFSN	1567.69:164.19=955%

Table S4: **The pouch cell detailed parameters at –80 °C.**

Parameter	Value
Cathode (current collector+materials)	0.956 g (220 mg+735.68 mg)
Anode (current collector+materials)	0.9145 g (492 mg+422.5 mg)
Tab	0.01332 g<br>0.4% (follow 20 Ah pouch cell)
Packaging materials	0.06728 g<br>2.02% (follow 20 Ah pouch cell)
Electrolyte (E/S=2.5)	1.313 g
Separator	0.147 g
Chip	0.0516 g<br>1.55% (follow module)
Magnetic field emitter	0.05895 g<br>1.77% (follow module)
Microsensor	0.00533 g<br>0.16% (follow module)
Temperature	–80 °C
Total mass	3.527 g

Discharge capacity	763.34 mAh
Energy consumption	367.98 mAh, 0.203% °C^{-1} of battery energy (module)
Current density	0.1 C
Specific capacity	1627.6 mAh g^{-1}
Energy density	469.9 Wh kg^{-1}
Energy density (with module weight)	454.5 Wh kg^{-1}
Energy density (with energy consumption and module weight)	219.1 Wh kg^{-1}
Capacity comparison with/without MFSN	1627.6:0

Table S5: **The pouch cell detailed parameters at –120 °C.**

Parameter	Value
Cathode (current collector+materials)	2.0353 g (440 mg+1595.29 mg)
Anode (current collector+materials)	1.9876 g (925 mg+1062.6 mg)
Tab	0.02908 g<br>0.4% (follow 20 Ah pouch cell)
Packaging materials	0.14688 g<br>2.02% (follow 20 Ah pouch cell)
Electrolyte (E/S=2.5)	2.8476 g
Separator	0.401 g
Chip	0.1127 g<br>1.55% (follow module)
Magnetic field emitter	0.1287 g<br>1.77% (follow module)
Microsensor	0.01163 g<br>0.16% (follow module)
Temperature	–120 °C
Total mass	7.7005 g

Discharge capacity	1782.22 mAh
Energy consumption	1826.9 mAh<br>0.38% °C^{-1} of battery energy
Current density	0.1 C
Specific capacity	1627.6 mAh g^{-1}
Energy density	502.5 Wh kg^{-1}
Energy density (with module weight)	486.03 Wh kg^{-1}
Energy density (with energy consumption and module weight)	Battery energy allows operation at this temperature
Capacity comparison with/without MFSN	1752.43:0

Table S6: The large pouch cell detailed parameters at –20 °C.

Parameter	Value
Cathode (current collector+materials)	26.05 g (3.54 g+22.51 g)
Anode (current collector+materials)	13.12 g (4.02 g+9.1 g)
Tab	0.34 g
Packaging materials	1.66 g
Electrolyte	38.43 g
Separator	2.65 g
Chip	5.1/4=1.275 g (1.55% follow module)
Magnetic field emitter	5.8/4=1.45 g (1.77% follow module)
Microsensor	0.13 g (0.16% follow module)
Temperature	–20 °C
Total mass	82.25 g
Total mass (with module weight)	85.105 g
Discharge capacity	22.63 Ah
Current density	0.1 C

Energy density	586.5 Wh kg^{-1}
Energy density (with module weight)	566.4 Wh kg^{-1}
Energy density (with energy consumption and module weight)	456.9 Wh kg^{-1}
Energy consumption	9.32 Wh

Table S7: The large pouch cell detailed parameters at –40 °C.

Parameter	Value
Cathode (current collector+materials)	26 g (3.54 g+22.46 g)
Anode (current collector+materials)	13.12 g (4.02 g+9.1 g)
Tab	0.34 g
Packaging materials	1.65 g
Electrolyte	38.39 g
Separator	2.63 g
Chip	5.1/4=1.275 g (1.55% follow module)
Magnetic field emitter	5.8/4=1.45 g (1.77% follow module)
Microsensor	0.13 g (0.16% follow module)
Temperature	–20 °C
Total mass	82.13 g
Total mass (with module weight)	84.985 g
Discharge capacity	24.12 Ah
Current density	0.1 C

Energy density	625.5 Wh kg^{-1}
Energy density (with module weight)	604.5 Wh kg^{-1}
Energy density (with energy consumption and module weight)	438.19 Wh kg^{-1}
Energy consumption	14.136 Wh

Table S8: The pouch cell detailed parameters at –80 °C.

Parameter	Value
Cathode (current collector+materials)	26.99 g (3.54 g+23.45 g)
Anode (current collector+materials)	13.12 g (4.02 g+9.1 g)
Tab	0.34 g
Packaging materials	1.68 g
Electrolyte	38.27 g
Separator	2.59 g
Chip	5.1/4=1.275 g (1.55% follow module)
Magnetic field emitter	5.8/4=1.45 g (1.77% follow module)
Microsensor	0.13 g (0.16% follow module)
Temperature	–80 °C
Total mass	82.99 g
Total mass (with module weight)	85.845 g
Discharge capacity	26.17 Ah
Current density	0.1 C

Energy density	671.7 Wh kg^{-1}
Energy density (with module weight)	649.3 Wh kg^{-1}
Energy density (with energy consumption and module weight)	314.9 Wh kg^{-1}
Energy consumption	28.71 Wh

Table S9: The pouch cell detailed parameters at –120 °C.

Parameter	Value
Cathode (current collector+materials)	26.83 g (3.54 g+23.29 g)
Anode (current collector+materials)	13.12 g (4.02 g+9.1 g)
Tab	0.34 g
Packaging materials	1.67 g
Electrolyte	37.96 g
Separator	2.63 g
Chip	5.1/4=1.275 g (1.55% follow module)
Magnetic field emitter	5.8/4=1.45 g (1.77% follow module)
Microsensor	0.13 g (0.16% follow module)
Temperature	–120 °C
Total mass	82.55 g
Total mass (with module weight)	85.405 g
Discharge capacity	27.12 Ah
Current density	0.1 C

Energy density	699.8 Wh kg^{-1}
Energy density (with module weight)	676.4 Wh kg^{-1}
Energy density (with energy consumption and module weight)	Battery energy allows operation at this temperature
Energy consumption	62.83 Wh

Table S10. Magnetic field activation timeline at -20 °C

Activation stage	Activation time (min)	Activation start	Activation end
1	2.64	2.01	4.65
2	3.01	7.11	10.12
3	2.86	12.72	15.58
4	3.28	17.92	21.2
5	3.27	23.26	26.53
6	3.07	29.28	32.35
7	3.29	34.47	37.76
8	3.14	39.81	42.95
9	3.29	45.14	48.43
10	3.01	50.62	53.63
11	3.43	55.78	59.71
12	3.35	61.29	64.64
13	3.28	67.03	70.31
14	3.28	72.64	75.92
15	3.15	78.11	81.26
16	3.15	83.31	86.46
17	3.42	88.51	91.93
18	2.6	93.98	96.58
19	3.3	98.9	102.2
20	3.69	104.65	108.34
21	3.38	109.89	113.27
22	3.29	115.18	118.47
23	3.01	120.52	123.53
24	3.83	125.72	129.55
25	3.42	132.01	135.43
26	3.28	137.76	141.04
27	3	143.37	146.37
28	2.74	148.7	151.44
29	2.98	153.93	156.91
30	3.15	159.37	162.52
31	2.87	165.12	167.99
32	2.87	170.59	173.46
33	2.88	175.92	178.8
34	3.01	181.26	184.27
35	2.87	186.73	189.6
36	2.87	191.93	194.8
37	3.29	197.26	200.55
38	3.15	203.01	206.16
39	2.74	208.89	211.63
40	3.01	214.09	217.1
41	2.87	219.7	222.57

42	3.01	224.9	227.91
43	2.73	230.51	233.24
44	2.87	235.84	238.71
45	2.6	241.31	243.91
46	3.01	246.37	249.38
47	3.28	251.85	255.13
48	3.14	257.46	260.6
49	2.74	263.06	265.8
50	2.87	268.4	271.27
51	2.87	273.87	276.74
52	3.28	279.21	282.49
53	2.87	285.09	287.96
54	2.6	290.42	293.02
55	2.94	295.49	298.43
56	3.31	301.07	304.38
57	2.93	306.98	309.91
58	3.03	312.35	315.38
59	2.73	317.65	320.38
60	2.97	322.88	325.85
61	3.14	328.32	331.46
62	3.03	334.04	337.07
63	2.87	339.4	342.27
64	2.73	344.6	347.33
65	3.01	349.79	352.8
66	3.01	355.13	358.14
67	3.07	360.78	363.85
68	3.01	366.35	369.36
69	2.74	371.82	374.56
70	3.42	377.02	380.44
71	2.87	382.9	385.77
72	2.76	388.31	391.07
73	3.01	393.43	396.44
74	2.95	398.83	401.78
75	3.01	404.38	407.39
76	3.28	409.85	413.13
77	3.01	415.73	418.74
78	3.01	421.2	424.21
79	3.01	426.54	429.55
80	3.01	432.15	435.16
81	2.87	437.76	440.63
82	3.28	443.09	446.37
83	2.75	448.97	451.72
84	2.84	454.17	457.01

85	3.01	459.64	462.65
86	2.74	465.25	467.99
87	3.28	470.53	473.81
88	2.9	476.31	479.21
89	2.7	481.67	484.37
90	2.73	486.87	489.6
91	3.01	492.2	495.21
92	2.88	497.67	500.55
93	2.97	503.08	506.05
94	3.14	508.76	511.9
95	2.58	514.77	517.35
96	2.31	519.03	521.34
97	2.72	523.82	526.54
98	2.31	529.01	531.32
99	2.5	533.89	536.39
Total time	297.27		

At -20 °C, the real-time total power consumption of the entire system is 2.92 W. In the calculation process, the no-load power consumption of 1.384 W is first subtracted to obtain the magnetic field power consumption of 1.536 W. Then, by adding the component power consumption of a pouch cell (1.384/4=0.346 W), the actual energy consumption required for a pouch cell is 1.882 W. The total duration of magnetic field activation is calculated to be ~297.27 min (4.9545 h). The actual total energy consumption is approximately 9.32 Wh (power × time). The energy density of the pouch cell (with module) with MFSN is 566.4 Wh kg^{-1}. Based on this energy consumption calculation, the system-level energy density of the pouch cell at -20 °C after deducting the energy consumption is 456.9 Wh kg^{-1}.

Table S11. Magnetic field activation timeline at -40 °C.

Activation stage	Activation time (min)	Activation start	Activation end
1	4.21	0.91	5.12
2	4.38	8.27	12.65
3	4.58	15.75	20.33
4	4.61	23.59	28.2
5	4.66	31.94	36.6
6	4.62	39.76	44.38
7	4.82	47.5	52.32
8	4.63	55.35	59.98
9	5.34	62.85	68.19
10	4.65	71.06	75.71
11	5.59	78.72	84.31
12	4.7	87.5	92.2
13	4.53	95.3	99.83
14	4.2	103.1	107.3
15	4.88	110.31	115.19
16	4.89	118.33	123.22
17	4.76	126.63	131.39
18	5.48	273	278.48
19	5.6	143.71	149.31
20	3.69	152.6	156.29
21	4.93	159.57	164.5
22	4.38	167.78	172.16
23	4.38	175.85	180.23
24	4.19	183.32	187.51
25	4.33	190.83	195.16
26	4.93	198.8	203.73
27	3.69	207.18	210.87
28	5.267	214.09	219.357
29	4.86	222.64	227.5
30	3.91	230.98	234.89
31	4.37	238.23	242.6
32	6.25	245.8	252.05
33	3.97	255.33	259.3
34	3.98	262.17	266.15
35	5.31	269.39	274.7
36	5.61	278.04	283.65
37	4.82	286.98	291.8
38	4.07	295.55	299.62
39	4.8	302.7	307.5
40	5.4	310.6	316
41	3.42	319.49	322.91

42	4.74	326.06	330.8
43	4.4	333.9	338.3
44	4.869	341.3	346.169
45	4.24	349.31	353.55
46	3.61	356.51	360.12
47	5.34	363.26	368.6
48	3.43	371.6	375.03
49	4.96	378.04	383
50	3.96	386.25	390.21
51	4.66	393.39	398.05
52	5.47	401.43	406.9
53	3.75	410.05	413.8
54	4.8	416.75	421.55
55	3.79	424.71	428.5
56	5.4	431.3	436.7
57	4.1	440.1	444.2
58	4.06	447.54	451.6
59	4.81	454.9	459.71
60	4.6	462.7	467.3
61	3.7	470.9	474.6
62	4.24	478.04	482.28
63	3.83	485.15	488.98
64	5.74	492.13	497.87
65	3.1	500.7	503.8
66	3.1	507.5	510.6
67	3.4	514.7	518.1
68	4.38	522.77	527.15
69	3.69	531.67	535.36
70	4.03	538.37	542.4
Total time	315.89		

The real-time power consumption of the magnetic field device, chip, and sensor after the magnetic field is activated at -40 °C is 3.723 W. In the calculation process, the no-load power consumption of 1.384 W is first subtracted to obtain the magnetic field power consumption of 2.339 W. Then, by adding the component power consumption of a pouch cell (0.346 W), the actual energy consumption required for a pouch cell is 2.685 W. The total duration of magnetic field activation is calculated to be ~315.89 min (5.264833 h). The actual total energy consumption is approximately 14.136 Wh (power × time). The energy density of the pouch cell (with module) with MFSN is 604.5 Wh kg^{-1}. Based on this energy consumption calculation, the system-level energy density of the

pouch cell at -40 °C after deducting the energy consumption is 438.19 Wh kg^{-1}.

Table S12. Magnetic field activation timeline at -80 °C

Activation stage	Activation time (min)	Activation start	Activation end
1	6.82	0	6.82
2	8.34	9.78	18.12
3	7.47	21.1	28.57
4	7.71	31.86	39.57
5	7.75	42.52	50.27
6	7.68	53.35	61.03
7	7.65	64.76	72.41
8	7.42	75.57	82.99
9	7.43	85.92	93.35
10	7.45	96.38	103.83
11	7.57	107.13	114.7
12	7.4	117.67	125.07
13	7.58	128.01	135.59
14	8.04	138.76	146.8
15	8.61	150.51	159.11
16	7.72	162.53	170.25
17	7.04	173.72	180.76
18	7.78	183.75	191.53
19	7.02	194.73	201.75
20	7.06	205.12	212.18
21	6.53	215.38	221.91
22	7.91	225.06	232.97
23	6.77	236.32	243.09
24	7.03	246.3	253.33
25	7.61	256.62	264.23
26	7.02	267.59	274.61
27	7.01	277.87	284.88
28	6.75	288.16	294.91
29	7.12	298.17	305.29
30	6.52	308.85	315.37
31	7.97	318.76	326.73
32	7.34	330.25	337.59
33	7.98	340.85	348.83
34	7.12	352.41	359.53
35	6.41	363.05	369.46
36	7.55	372.72	380.27
37	7.43	383.39	390.82
38	7.56	393.91	401.47
39	6.65	404.58	411.23
40	7.41	414.36	421.77
41	7.68	425.35	433.03

42	6.82	436.15	442.97
43	7.11	446.08	453.19
44	7.64	456.61	464.25
45	6.96	467.86	474.82
46	7.13	478.09	485.22
47	6.51	488.32	494.83
48	7.12	498.09	505.21
49	7.28	508.37	515.65
50	7.07	519.18	526.25
51	7.11	529.22	536.33
52	7.1	539.75	546.85
53	7.11	550.41	557.52
54	6.02	560.85	566.87
55	6.93	570.15	577.08
Total time	400.82		

The real-time power consumption of the magnetic field device, chip, and sensor after the magnetic field is activated at -80 °C is 5.336 W. In the calculation process, the no-load power consumption of 1.384 W is first subtracted to obtain the magnetic field power consumption of 3.952 W. Then, by adding the component power consumption of a pouch cell (0.346 W), the actual energy consumption required for a pouch cell is 4.298 W. The total duration of magnetic field activation is calculated to be ~400.82 min (6.142333 h). The actual total energy consumption is approximately 28.71 Wh (power × time). The energy density of the pouch cell (with module) with MFSN is 649.3 Wh kg^{-1}. Based on this energy consumption calculation, the system-level energy density of the pouch cell at -80 °C after deducting the energy consumption is 314.9 Wh kg^{-1}.

Table S13. Magnetic field activation timeline at -120 °C

Activation stage	Activation time (min)	Activation start	Activation end
1	6.62	0	6.62
2	11.1	10.21	21.31
3	11.33	26.97	38.3
4	11.81	42.61	54.42
5	13.58	60.17	73.75
6	12.83	78.52	91.35
7	12.24	95.28	107.52
8	11.96	112.57	124.53
9	13.77	128.91	142.68
10	13.31	146.83	160.14
11	16.44	165.66	182.1
12	11.81	187.05	198.86
13	8.94	203.17	212.11
14	10.85	216.09	226.94
15	17.4	231.89	249.29
16	12.13	254.72	266.85
17	6.7	271.79	278.49
18	10.28	282.28	292.56
19	11.83	295.89	307.72
20	9.89	311.22	321.11
21	10.88	325.1	335.98
22	10.21	339.31	349.52
23	9.26	354.15	363.41
24	9.57	367.72	377.29
25	9.08	381.6	390.68
26	8.62	395.01	403.63
27	9.42	408.41	417.83
28	8.62	422.46	431.08
29	9.74	435.54	445.28
30	9.45	449.27	458.72
31	9.89	462.52	472.41
32	9.1	475.92	485.02
33	8.62	489.33	497.95
34	9.73	501.94	511.67
35	8.3	515.34	523.64
36	9.42	527.95	537.37
37	7.98	541.04	549.02
38	8.95	553.32	562.27
39	7.66	566.26	573.92
40	7.98	578.54	586.52
41	7.18	590.51	597.69

Total time	424.48		

The real-time power consumption of the magnetic field device, chip, and sensor after the magnetic field is activated at -120 °C is 9.919 W. In the calculation process, the no-load power consumption of 1.384 W is first subtracted to obtain the magnetic field power consumption of 8.535 W. Then, by adding the component power consumption of a pouch cell (0.346 W), the actual energy consumption required for a pouch cell is 8.881 W. The total duration of magnetic field activation is calculated to be ~424.48 min (7.075 h). The actual total energy consumption is approximately 62.83 Wh (power × time). The energy density of the pouch cell (with module) with MFSN is 676.4 Wh kg^{-1}. The energy of pouch cell is essentially sufficient to sustain its operation at -120 °C.

Table 14. The detailed parameters of the magnetic field device and temperature detectors.

Parameter	Value
Chip（Coil drive module + temperature sampling module + control module）	5.1 g
Magnetic field emitter	5.8 g
Microsensor	0.13 g

Section 5. Supplementary Movie (separate file)

Movie S1: Real-time driving of the UAV under dry ice conditions.

Drive the UAV in real time through the pouch cell with multi-field coupling intelligent structure in the dry ice pile.

Supplementary References:

1. Lu, T., and Chen, F. Multiwfn: A multifunctional wavefunction analyzer. *J Comput Chem* 2012; **33**: 580−592.
2. Humphrey, W. *et al*. VMD: Visual molecular dynamics. *J. Molec. Graphics* 1996; **14**: 33-38.
3. Stephens, P. J., Devlin, F. J., Chabalowski, C. F., and Frisch, M. J. Ab Initio Calculation of Vibrational Absorption and Circular Dichroism Spectra Using Density Functional Force Fields. *J. Phys. Chem* 1994; **98**: 11623-11627.
4. (a) Grimme, S., Ehrlich, S., and Goerigk, L. Effect of the damping function in dispersion corrected density functional theory. *J Comput Chem* 2011; **32**: 1456–1465. (b) Grimme, S., Antony, J., Ehrlich, S. and Krieg, H. A consistent and accurate ab initio parametrization of density functional dispersion correction (DFT-D) for the 94 elements H-Pu. *J. Chem. Phys* 2010; **132**: 154104.
5. (a) Hariharan, P. C., Pople, J. A. *Theor. Chim. Acta.* **28**, 213-222 (1973). (b) Hehre, W. J., Ditchfield, R., Pople, J. A. Self-Consistent Molecular Orbital Methods. XII. Further Extensions of Gaussian—Type Basis Sets for Use in Molecular Orbital Studies of Organic Molecules. *J. Chem. Phys* 1972; **56**: 2257-2261.
6. Geuenich, D., Hess, K., Köhler, F., and Herges, R. Anisotropy of the Induced Current Density (ACID), a General Method To Quantify and Visualize Electronic Delocalization. *Chemical Reviews* 2005; **105**: 3758-3772.
7. Lu, D., Li, R., Rahman, M. M., Yu, P. Y., Lv, L., Yang, S., Huang, Y. Q., Sun, C. C., Hu, C. S., Wang, C. S., Fan, X. L. Ligand-channel-enabled ultrafast Li-ion conduction. *Nature* 2024; **627**: 101−107.
8. Xu, J. J., Zhang, J. X., Pollard, T. P., Li, Q. D., Tan, S., Hou, S. Y., Wan, H. L., Chen, F., Borodin. O., Wang, C. S., et al. Electrolyte design for Li-ion batteries under extreme operating conditions. *Nature* 2023; **614**: 694-700.
9. Jiang, L. W., Hu, Y. C., Ai, F., Liang, Z. J., and Lu, Y. C. Rational design of anti-freezing electrolyte concentrations via freeze concentration process. *Energy Environ. Sci* 2024; **17**: 2815–2824.

10. Ji, H. Q., Wang, Z. K., Sun, Y. W., Zhou, Y., Li, S. J., Zhou, J. Q., Qian, T., and Yan, C. L. Weakening Li+ De-solvation Barrier for Cryogenic Li-S Pouch Cells. *Adv. Mater* 2023; **35**: 2208590.
11. Han, Z. Y., Gao, R. H., Wang, T. S., Tao, S. Y., Jia, Y. Y., Lao, Z. J., Zhang, M. T., Zhou, J. Q., Li, C., Piao, Z. H., Zhang, X., and Zhou, G. M. Machine-learning-assisted design of a binary descriptor to decipher electronic and structural effects on sulfur reduction kinetics. *Nat. Catal* 2023; **6**: 1073-1086.
12. Kwon, H., Kim, H. S., Hwang, J., Oh, W., Roh, Y., Shin, D., and Kim, H. -T. Borate-pyran lean electrolyte-based Li-metal batteries with minimal Li corrosion. *Nat. Energy* 2023; **9**: 57-69.
13. Niu, C. J., Pan, H. L., Xu, W., Xiao, J., Zhang, J. G., Luo, L. L., Wang, C. M., Mei, D. H., Meng, J. S., Wang, X. P., Liu, Z., Mai, L. Q., and Liu, J. Self-smoothing anode for achieving high-energy lithium metal batteries under realistic conditions. *Nat. Nanotechnol* 2021; **14**: 166-173.
14. Niu, C. J., Liu, D. Y., Lochala, J. A., Anderson, C. S., Cao, X., Gross, M. E., Xu, W., Zhang, J. G., Whittingham, M. S., Xiao, J., and Liu, J. Balancing interfacial reactions to achieve long cycle life in high-energy lithium metal batteries. *Nat. Energy* 2021; **6**: 723-732.
15. Zhang, W. L., Lu, Y., Cao, Q. B., Liu, H., Feng, Q. Q., Zhou, P., Xia, Y. C., Hou, W. H., Yan, S. S., and Liu, K. A reversible self-assembled molecular layer for lithium metal batteries with high energy/power densities at ultra-low temperatures. *Energy Environ. Sci* 2024; **17**: 4531-4543.
16. Holoubek, J., Liu, H. D., Wu, Z. H., Yin, Y. J., Xing, X., Cai, G. R., Yu, S. C., Zhou, H. Y., Pascal, T. A., Chen, Z., and Liu, P. Tailoring electrolyte solvation for Li metal batteries cycled at ultra-low temperature. *Nat. Energy* 2021; **6**:303-313.
17. Wan, H. L., Wang, Z. Y., Zhang, W. R., He, X. Z., and Wang, C. S. Interface design for all-solid-state lithium batteries. *Nature* 2023; **623**: 739–744.

18. Ye, Y. S., Xu, R., Huang, W. X., Ai, H. Y., Zhang, W. B., Affeld, J. O., Cui, A., Liu, F., Nakayama, Y. R., and Cui, Y. Quadruple the rate capability of high-energy batteries through a porous current collector design. *Nat. Energy* 2024; **9**: 643-653.
19. Li, Y. Q., Zhou, Q., Weng, S. T., Ding, F. X., Qi, X. G., Lu, J. Z., Li, Y., Zhang, X., Chen, L. Q., and Hu, Y. S. Interfacial engineering to achieve an energy density of over 200 Wh kg−1 in sodium batteries. *Nat. Energy* 2022; **7**: 511-519.
20. Li, Y. Q., Zhou, Q., Weng, S. T., Ding, F. X., Qi, X. G., Lu, J. Z., Li, Y., Zhang, X., Chen, L. Q., and Hu, Y. S. Interfacial engineering to achieve an energy density of over 200 Wh kg−1 in sodium batteries. *Nat. Energy* 2022; **7**: 511-519.
21. Jiang, L. W., Han, S., Hu, Y. C., Yang, Y., Lu, Y. X., Lu, Y. C., Zhao, J. M., Chen, L. Q., and Hu, Y. S. Rational design of anti-freezing electrolytes for extremely low-temperature aqueous batteries. *Nat. Energy* 2024; **9**: 839-848.
22. Li, Z. N., Sami, I., Yang, J., Li, J. T., Kumar, R. V., and Chhowalla, M. Lithiated metallic molybdenum disulfide nanosheets for high-performance lithium–sulfur batteries. *Nat. Energy* 2023; **8**: 84-93.
23. Jin, T., Zhao, M., Li, X. Y., Chen, Z. X., Li, B. Q., Huang, J. Q., Zhang, Q. Reducing polysulfide hydrodynamic radius toward low-temperature lithium-sulfur batteries. *Chem* 2026; **12**; 102881.
24. Ji, H. Q., Wang, Z. K., Sun, Y. W., Zhou, Y., Li, S. J., Zhou, J. Q., Qian, T., Yan, C. L. Weakening Li^+ De-solvation Barrier for Cryogenic Li-S Pouch Cells. *Adv. Mater.* 2023; **35**; 2208590.
25. Wang, P., Xu, T. Y., Xiong, S. L., Wang, Y., Sun, D., Song, N., Feng, J. K., Xi, B. Q. Ligand Engineering-Enhanced Catalytic Activity of Octanuclear Zn (II)-Siloxane Clusters for Advanced Lithium-Sulfur Batteries. *Angew. Chem. Int. Ed.* 2025; **64**; e202516197.
